# BIG enhances Arg/N-degron pathway-mediated protein degradation to regulate Arabidopsis hypoxia responses and suberin deposition

**DOI:** 10.1093/plcell/koae117

**Published:** 2024-04-12

**Authors:** Hongtao Zhang, Chelsea Rundle, Nikola Winter, Alexandra Miricescu, Brian C Mooney, Andreas Bachmair, Emmanuelle Graciet, Frederica L Theodoulou

**Affiliations:** Plant Sciences and the Bioeconomy, Rothamsted Research, Harpenden, AL5 2JQ, UK; Plant Sciences and the Bioeconomy, Rothamsted Research, Harpenden, AL5 2JQ, UK; Department of Biochemistry and Cell Biology, Max Perutz Labs, University of Vienna, Vienna, Austria; Department of Biology, Maynooth University, Maynooth, Ireland; Department of Biology, Maynooth University, Maynooth, Ireland; Department of Biochemistry and Cell Biology, Max Perutz Labs, University of Vienna, Vienna, Austria; Department of Biology, Maynooth University, Maynooth, Ireland; Plant Sciences and the Bioeconomy, Rothamsted Research, Harpenden, AL5 2JQ, UK

## Abstract

BIG/DARK OVEREXPRESSION OF CAB1/TRANSPORT INHIBITOR RESPONSE3 is a 0.5 MDa protein associated with multiple functions in Arabidopsis (*Arabidopsis thaliana*) signaling and development. However, the biochemical functions of BIG are unknown. We investigated a role for BIG in the Arg/N-degron pathways, in which substrate protein fate is influenced by the N-terminal residue. We crossed a *big* loss-of-function allele to 2 N-degron pathway E3 ligase mutants, *proteolysis6* (*prt6*) and *prt1*, and examined the stability of protein substrates. Stability of model substrates was enhanced in *prt6-1 big-2* and *prt1-1 big-2* relative to the respective single mutants, and the abundance of the PRT6 physiological substrates, HYPOXIA-RESPONSIVE ERF2 (HRE2) and VERNALIZATION2 (VRN2), was similarly increased in *prt6 big* double mutants. Hypoxia marker expression was enhanced in *prt6 big* double mutants; this constitutive response required arginyl transferase activity and RAP-type Group VII ethylene response factor (ERFVII) transcription factors. Transcriptomic analysis of roots not only demonstrated increased expression of multiple hypoxia-responsive genes in the double mutant relative to *prt6*, but also revealed other roles for PRT6 and BIG, including regulation of suberin deposition through both ERFVII-dependent and independent mechanisms, respectively. Our results show that BIG acts together with PRT6 to regulate the hypoxia-response and broader processes in Arabidopsis.

## Teaser

The large protein BIG participates in the Arg/N-degron pathways to regulate hypoxia responses and broader functions in *Arabidopsis thaliana*.

**IN A NUTSHELL**

**Background:** BIG—as the name suggests—is an enormous protein found in plants. When mutated, it has a dramatic effect on plants, but almost nothing is known about what it does at a molecular level. Knocking out the corresponding gene (*UBR4*) in animals also affects multiple processes. Several studies have shown that UBR4 is a ubiquitin E3 ligase involved in different protein degradation pathways. These include the N-degron pathways, in which proteins are selectively cleaved, and the identity of the new amino (N-) terminal amino acid influences their fate.

**Question:** We set out to test whether BIG is involved in the N-degron pathways. Because the BIG protein is difficult to work with, we tested whether model and physiological substrates were stabilized in *big* and other mutants. We also searched for proteins that could bind model substrates using proximity labeling.

**Findings:** Using model X-GUS substrates, we showed that BIG works together with 2 known components of the N-degron pathway, PRT6 and PRT1, to mediate the degradation of substrates with different classes of N-termini. Physiological substrates, ETHYLENE RESPONSE FACTOR (ERFVII) transcription factors and VERNALIZATION2, accumulated in *prt6 big* mutants. ERFVIIs control plant hypoxia responses, and the expression of hypoxia response genes was enhanced in *prt6 big* mutants. Interestingly, genes involved in the synthesis and deposition of suberin, a complex polymer that can act as a barrier to nutrients and gases, were downregulated in *prt6 big* mutant roots. The stabilization of ERFVIIs partly explained this effect. Proximity labeling suggests that BIG, PRT6, and HECT-type E3 ligases may be present in a complex at the proteasome.

**Next Steps:** We would like to identify other proteins that interact with BIG, pinpoint the specific roles of different protein domains, figure out what other roles BIG plays in protein homeostasis, and connect these to the dramatic phenotypes of the *big* mutant.

## Introduction

**Figure 1.**
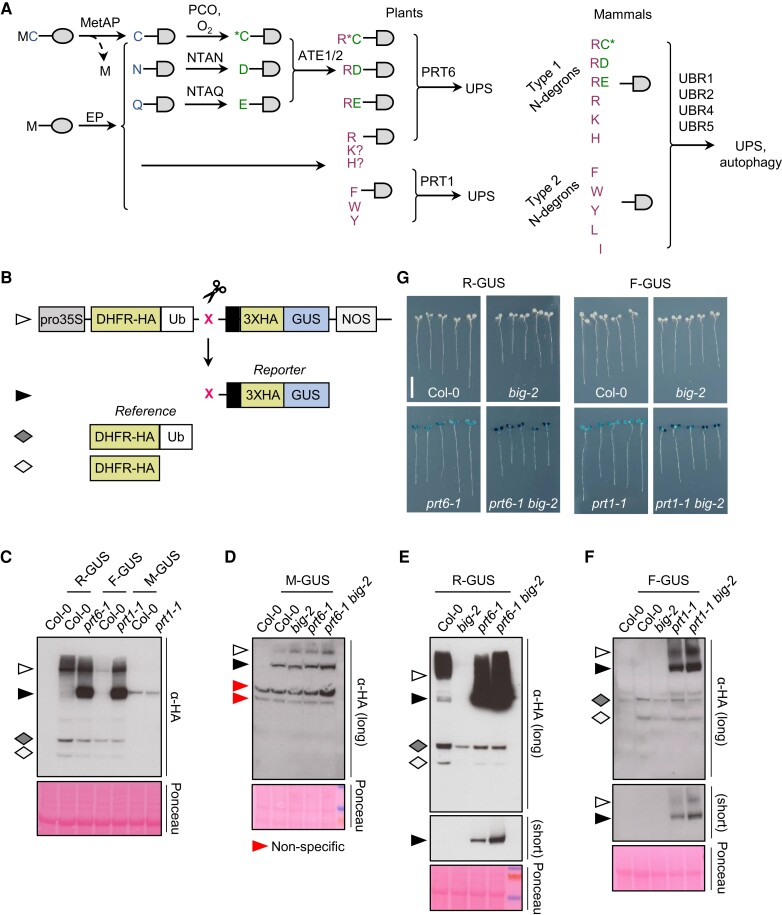
BIG influences the stability of model Types 1 and 2 Arg/N-degron pathway substrates. **A)** Schematic showing the architecture of the Arg/N-degron pathway and the specificity of N-recognins in plants and mammals. Single letter codes for amino acid residues are used; *C indicates oxidized cysteine. Proteins (represented by shaded ovals) may become N-degron pathway substrates via cleavage by nonprocessive endopeptidases (EP), or by methionine aminopeptidase (MetAP), where the second residue is small. Substrates may also be generated by enzymatic modification of N-termini by PCO, Asn-specific N-terminal amidase (NTAN), Gln-specific N-terminal amidase (NTAQ), and ATE. In plants, destabilizing residues thus generated are targeted for degradation by the Ubiquitin Proteasome System (UPS) via N-recognin E3 ligases PROTEOLYSIS6 (PRT6; specific for basic N-termini) and PROTEOLYSIS1 (PRT1; specific for aromatic N-termini). In mammals, 4 N-recognins act semi-redundantly to mediate the degradation of both Types 1 and 2 substrates via the UPS or by autophagy. **B)** Generation of N-degron pathway X-GUS substrates. Constructs driven by the constitutive *CaMV35S* promoter (Pro35S) encode a fusion of mouse DHFR to ubiquitin (variant K48R; Ub), followed by *E. coli* beta-GUS. Ubiquitin-specific proteases (indicated by the scissors icon) remove ubiquitin co-translationally to release the GUS reporter protein and reveal a new N terminus (residue of choice, X). The GUS ORF is extended by unstructured amino acids to enhance the effect of destabilizing amino-terminal residues. The cleavage also creates a stable DHFR reference protein and HA epitopes enable immunological detection of both products (Garzón et al. 2007). NOS, nopaline synthase terminator. Note that the stable reference carries 1 copy of the HA epitope, whereas the reporter has 3 copies. **C–F)** The detection of N-degron pathway substrates by immunoblotting of crude protein extracts from 6-d-old seedlings of different genotypes expressing X-GUS reporters. The symbols to the left indicate the protein products shown in (**B**). Blots were developed until the stable reference protein could be detected (α-HA long); where the stabilized reporter band signal is saturated, a shorter exposure is shown in the lower panel (short) for clarity. Ponceau S staining was used to confirm equal loading. **G)** Histochemical staining of GUS reporter activity in 6-d-old seedlings expressing R-GUS and F-GUS test substrates. Representative seedlings were rearranged on an agar plate, prior to photography. Bar: 1 cm (images are scaled identically).

Targeted protein degradation is an important proteostatic mechanism that influences a multitude of agronomically important traits in plants (Linden and Callis 2020; Theodoulou et al. 2022) and represents a major target for drug development in humans (Ciechanover 2013; Kannt and Đikić 2021). The Arg/N-degron pathways (formerly known as the Arg/N-end rule pathways) constitute a specialized form of proteostasis in which the N-terminal (Nt) residue of a given protein is the key determinant of a degradation signal, known as an N-degron (Bachmair et al. 1986; Varshavsky 2019). N-degrons are revealed by protein cleavage by nonprocessive endopeptidases and/or created by subsequent enzymatic modification of the neo-N-terminus by amidases and arginyl-tRNA transferase enzymes (ATEs; Fig. 1A). In mammals and yeast, N-degrons include Type 1 positively charged residues (Arg, Lys, and His) and Type 2 bulky hydrophobic residues (Trp, Phe, Tyr, Leu, and Ile), which are recognized by proteins known as N-recognins that facilitate substrate degradation.

The prototypical N-recognin, ubiquitin amino-end recognizing protein 1 (Ubr1) of Baker's yeast (*Saccharomyces cerevisiae*) accepts both Types 1 and 2 substrates, whereas in mammals, 4 N-recognins, UBR1, UBR2, UBR4, and UBR5, which share a conserved UBR box domain, act semi-redundantly to mediate proteasomal and autophagic degradation of Arg/N-degron pathway substrates (Tasaki et al. 2005, 2009, 2013). In contrast, plants contain N-recognins with discrete substrate specificities (Garzón et al. 2007). PROTEOLYSIS6 (PRT6), the Arabidopsis (*Arabidopsis thaliana*) homolog of yeast Ubr1 and mammalian UBR1/2, is a candidate E3 ligase with specificity for basic N-termini (Arg, Lys, and His), and PROTEOLYSIS1 (PRT1) is an unrelated ZZ domain protein with E3 ligase activity toward protein substrates bearing aromatic N-termini (Phe, Tyr, and Trp) (Potuschak et al. 1998; Stary et al. 2003; Garzón et al. 2007; Graciet et al. 2010; Mot et al. 2018). Experimental evidence indicates the existence of a further (still unknown) N-recognin class that targets bulky/hydrophobic-N-termini (Leu and Ile; Garzón et al. 2007; Graciet et al. 2010).

The availability of mutants and transgenics in which N-recognin function is disrupted has revealed diverse functions for the Arg/N-degron pathway in plants (Holdsworth et al. 2020). Relatively little is known regarding the PRT1/N-degron pathway, although it has been shown to influence defense responses (de Marchi et al. 2016; Till et al. 2019) and the turnover of the E3 ligase BIG BROTHER (Dong et al. 2017). In contrast, the PRT6/N-degron pathway plays multiple roles in development (Yoshida et al. 2002; Choy et al. 2008; Graciet et al. 2009; Holman et al. 2009; Gibbs et al. 2014, 2018; Abbas et al. 2015; Zhang et al. 2018a, b; Weits et al. 2019; Labandera et al. 2021), plant–pathogen interactions (de Marchi et al. 2016; Gravot et al. 2016; Vicente et al. 2019), and responses to the abiotic environment (Gibbs et al. 2011; Licausi et al. 2011; Abbas et al. 2015, 2022; Weits et al. 2014; Mendiondo et al. 2016; Vicente et al. 2017; Hartman et al. 2019; Lamichhane et al. 2020; Lou et al. 2022).

The first substrates of the PRT6/N-degron pathway were identified in the context of oxygen sensing (Gibbs et al. 2011; Licausi et al. 2011). Arabidopsis has 5 Group VII ethylene response factor transcription factors (ERFVIIs) bearing a conserved cysteine residue at Position 2, of which *RELATED TO APETALA (RAP) 2.12*, *RAP2.2*, and *RAP2.3* are constitutively expressed, whereas *HYPOXIA-RESPONSIVE ERF* (*HRE*) *1* and *HRE2* are induced by low oxygen (Licausi et al. 2010). All 5 Met-Cys-ERFVII proteins undergo co-translational Nt Met excision to reveal Nt Cys, which under normoxia is susceptible to oxidation by plant cysteine oxidase (PCO) enzymes and subsequent arginylation by ATEs (Weits et al. 2014; White et al. 2017). N-terminally arginylated ERFVIIs are then thought to be recognized by PRT6, which targets the proteins for proteasomal degradation (Gibbs et al. 2011; Licausi et al. 2011). However, when oxygen is limiting, ERFVIIs are stabilized and coordinate the transcriptional response to hypoxia. Consequently, hypoxia-responsive genes, such as *ALCOHOL DEHYDROGENASE* (*ADH*) and *PHYTOGLOBIN1* (*PGB1*) (as well as *HRE1* and *HRE2*), are ectopically expressed in *prt6* alleles (Choy et al. 2008; Gibbs et al. 2011; Riber et al. 2015). The Arabidopsis genome encodes 248 Met-Cys initiating proteins, of which the polycomb repressive complex 2 subunit, VERNALIZATION 2 (VRN2) and the transcription factor LITTLE ZIPPER 2 (ZPR2) have also been confirmed as oxygen-sensitive physiological PRT6/N-degron pathway substrates with roles in development (Gibbs et al. 2018; Weits et al. 2019; Labandera et al. 2021).

Experimental evidence and sequence database searches indicate that the full suite of N-recognins has not yet been identified in plants (Garzón et al. 2007; Graciet et al. 2010). Arabidopsis has 3 UBR box proteins: PRT6, BIG (also known as DARK OVEREXPRESSION OF CAB1; DOC1 and TRANSPORT INHIBITOR RESPONSE3; TIR3), and AT4G23860 (Tasaki et al. 2005). In this study, we investigated a potential role for BIG in the Arg/N-degron pathways, since its mammalian and Drosophila (*Drosophila melanogaster*) homologs (UBR4 and Calossin/Pushover, respectively) are known N-recognins (Tasaki et al. 2005, 2009; Ashton-Beaucage et al. 2016; Yoo et al. 2018; Hunt et al. 2019). Beyond the N-degron pathway, UBR4 has been implicated in proteasomal, autophagosomal, and lysosomal degradation of cytoplasmic and membrane proteins (Lin et al. 2013; Tasaki et al. 2013; Hong et al. 2015; Kim et al. 2018; Hunt et al. 2019) and contributes to protein quality control (Yau et al. 2017; Tang et al. 2020; Hunt et al. 2021). UBR4 interacts with E3 ligases of different classes (Ashton-Beaucage et al. 2016; Yau et al. 2017; Hunt et al. 2019) and is also proposed to be an E3 ligase, largely based on genetic evidence. However, only relatively recently has its E3 ligase activity been characterized biochemically (Yau et al. 2017; Hunt et al. 2019; Tang et al. 2020) and shown to require a noncanonical hemi-Really Interesting New Gene (hemi-RING) domain, that is conserved in BIG (Barnsby-Greer et al. 2024).

*BIG* has been identified in around 20 different forward genetic screens and associated with diverse physiological functions via reverse genetics in Arabidopsis. The first *big* allele, *dark overexpression of CAB* (*doc1-1*), was isolated in a screen for mutants with misregulated photosynthetic gene expression (Li et al. 1994). *doc1-1*, which displays a striking morphological phenotype of reduced apical dominance and small stature, was subsequently found to be allelic to *transport inhibitor response3* (*tir3-1*), a mutant compromised in auxin transport (Ruegger et al. 1997; Gil et al. 2001). The affected gene was identified via map-based cloning and renamed in recognition of its exceptional size: *BIG,* which is expressed throughout the plant, encodes a 5,077 amino acid protein with a predicted molecular weight of 565,597 Da (Gil et al. 2001; He et al. 2018). *BIG* was later shown to influence multiple hormone signaling pathways and different aspects of plant development (Kanyuka et al. 2003; Desgagné-Penix et al. 2005; Yamaguchi et al. 2007; Guo et al. 2013; Shinohara et al. 2013; Yamaguchi and Komeda 2013; Zhang et al. 2020; Liu et al. 2022). Recent studies indicate further, apparently disparate functions for *BIG* in the circadian clock, guard cell signaling, calcium homeostasis, regulation of C/N balance, response to pathogens, cell death, and wound-induced rooting (Üstün et al. 2016; Meteignier et al. 2017; He et al. 2018; Hearn et al. 2018; Zhang et al. 2019a, b; Bruggeman et al. 2020; Modrego et al. 2023). Although many *big* mutant phenotypes can be ascribed to dysregulation of auxin transport (Li et al. 1994; Ruegger et al. 1997; Gil et al. 2001; López-Bucio et al. 2005; Kasajima et al. 2007; Yamaguchi et al. 2007; Guo et al. 2013; Yamaguchi and Komeda 2013; Ivanova et al. 2014; Wu et al. 2015; Zhang et al. 2020), this is not the case for all processes influenced by BIG and to date its precise biochemical functions have remained unclear.

In this study, we demonstrate that BIG participates in the Arg/N-degron pathways, acting semi-redundantly with PRT6 and PRT1. PRT6/N-degron pathway substrates hyperaccumulate in *prt6 big* double mutants, enhancing the molecular response to hypoxia in an ERFVII-dependent fashion. This was confirmed by RNA-seq analysis which also indicated a range of different genetic interactions between *big-2* and *prt6-5* that influence transcription of additional groups of genes, pointing to broader functions for BIG and PRT6, including the regulation of suberin deposition.

## Results

### BIG influences the stability of model Arg/N-degron pathway substrates

To test whether BIG plays a role in the Arg/N-degron pathways, we used the ubiquitin fusion technique to produce pathway substrates in planta (Varshavsky 2000). We took advantage of the DHFR-Ub-X-GUS system (Garzón et al. 2007), in which a genetically encoded ubiquitin domain is cleaved in vivo by deubiquitinating enzymes to produce a reporter protein, β-glucuronidase (GUS) bearing a residue of choice (X) at the N-terminus, and a stable reference protein, dihydrofolate reductase (DHFR; Fig. 1B). Lines expressing constructs designed to release a Type 1, basic Nt substrate (R-GUS), a Type 2, aromatic Nt substrate (F-GUS), and a stable control (M-GUS) were generated in the wild-type Arabidopsis accession, Columbia-0 (Col-0), and in different mutant backgrounds lacking known N-recognins and *BIG*. Details of the *big-2* allele used in this study and originally described in (Kasajima et al. 2007) are provided in Supplementary Fig. S1 and Table S1.

The stability of X-GUS was assessed by immunoblotting and histochemical staining. Immunoblotting revealed that the fusion proteins were cleaved as predicted and that R-GUS and F-GUS were unstable in Col-0 wild-type seedlings, relative to the DHFR control (Fig. 1C). R-GUS and F-GUS were stabilized in *prt6-1* and *prt1-1* mutants, respectively, as previously reported (Garzón et al. 2007; Zhang et al. 2018a). In contrast, M-GUS was stable in all backgrounds tested (Fig. 1, C and D). R-GUS and F-GUS reporters were not stabilized in the *big-2* single mutant, but stability of R-GUS was enhanced in the *prt6-1 big-2* double mutant compared with *prt6-1* (Fig. 1E). Similarly, F-GUS was more stable in *prt1-1 big-2* than in *prt1-1* (Fig. 1F). Histochemical staining for GUS activity was consistent with these results (Fig. 1G). Together, these data indicate that BIG acts together with known N-recognins to mediate the degradation of substrates initiating with R and F. As an independent test, a cleavable R-luciferase (R-LUC) reporter (Worley et al. 1998; Graciet et al. 2010) was also introduced into *prt6-5 big-2* (Supplementary Fig. S2A). R-LUC was unstable in Col-0 and *big-2* but detected in both *prt6-5* and *prt6-5 big-2*, with higher luciferase activity in the double mutant, consistent with enhanced stabilization of the R-LUC protein in *prt6-5 big-2* (Supplementary Fig. S2B).

**Figure 2.**
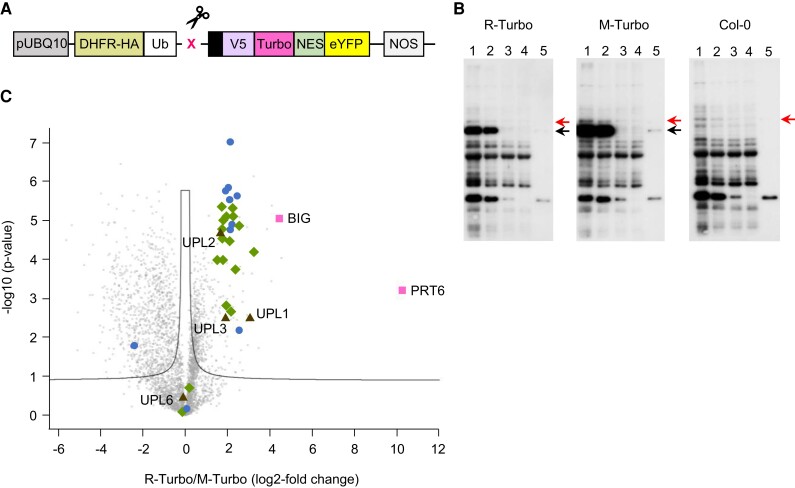
Identification of N-degron proximal proteins. **A)** Schematic of construct used for proximity labeling assay. Turbo-NES-eYFP (Mair et al. 2019) was modified to harbor an N-terminal ubiquitin (Ub) fusion construct, comprising dihydrofolate reductase-HA epitope-Ub (DHFR-HA-Ub) followed by the N-terminal amino acid of choice (X) in front of a fusion comprising a linker sequence, the V5 epitope, modified *E. coli* biotin ligase (Turbo), a nuclear export signal (NES), and enhanced yellow fluorescent protein (eYFP). The construct was expressed from the *POLYUBIQUITIN10* promoter (ProUBQ10). The fusion protein is cleaved in planta by ubiquitin-specific proteases, indicated by the scissors icon. **B)** Anti-Biotin immunoblots to follow the enrichment of biotinylated protein in extracts from 7-d-old Col-0 and plants expressing R-Turbo and M-Turbo. Lane 1, crude extract; Lane 2, extract after desalting; Lanes 3 and 4, supernatant after 2 subsequent incubations with streptavidin beads (unbound fractions); Lane 5, streptavidin beads after first incubation. Black arrows indicate the bait proteins R-Turbo and M-Turbo (70.2 kDa), and the red arrow indicates a nonspecific background band. **C)** Volcano plot visualizing enrichment of biotinylated proteins when comparing transgenic lines expressing R-Turbo versus M-Turbo, after proteasome inhibition to ensure equal presence of either protein. PRT6 and BIG (squares) are the 2 most enriched proteins. Diamonds, non-ATPase subunits of the proteasome; circles, ATPase subunits; triangles, HECT E3 ubiquitin ligases.

To complement the genetic approach, potential protein interactions with N-degrons were investigated using proximity labeling (Mair et al. 2019). Transgenic lines expressing a modified *Escherichia coli* biotin ligase (TurboID)-YFP fusion designed to reveal either an Nt M- or R- residue (Fig. 2A) were generated in the Col-0 background. Proteins biotinylated by the TurboID fusions were enriched on streptavidin beads (Fig. 2B) and analyzed by MS. Both PRT6 and BIG were enriched in the R-TurboID sample relative to the M-TurboID sample, indicating their proximity to R-TurboID in planta, and suggesting a potential physical interaction of Nt Arg residues with PRT6 and BIG (Fig. 2C). Interestingly, regulatory proteasomal subunits and homologous to the E6AP carboxyl C-terminus (HECT) ubiquitin E3 ligases known to be associated with the proteasome (Wang and Spoel 2022) were also highly enriched in the R-TurboID sample (Fig. 2C; Supplementary Data Set 1).

### BIG influences the abundance of physiological PRT6/N-degron pathway substrates

**Figure 3.**
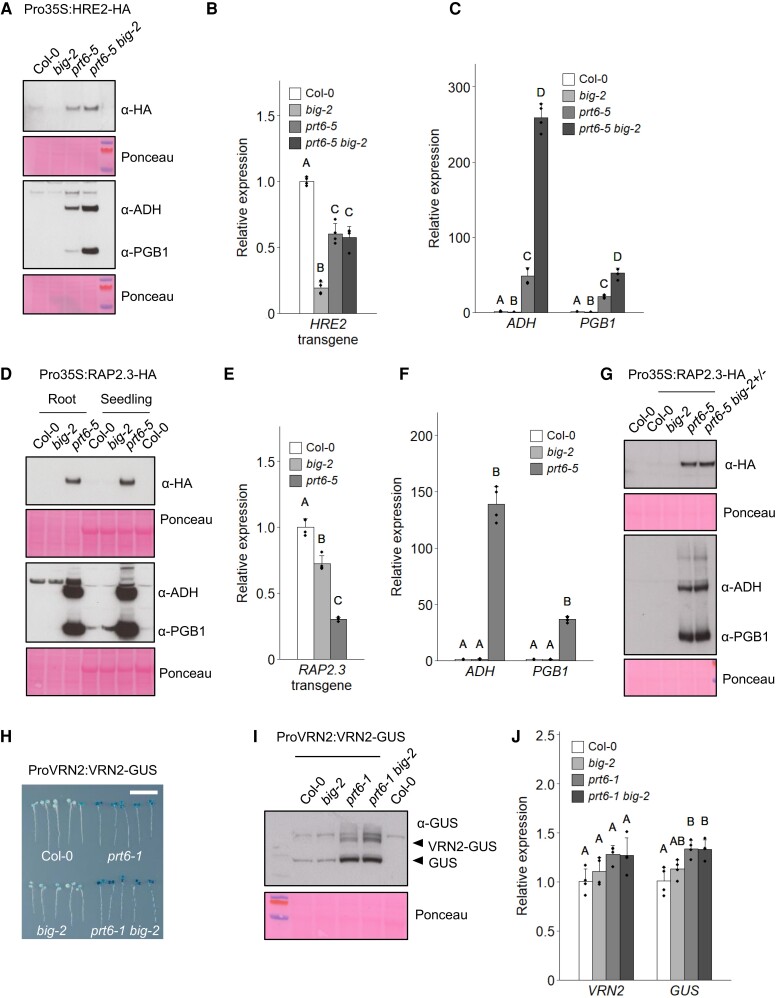
BIG influences the abundance of physiological PRT6/N-degron pathway substrates. **A–C)** Molecular analysis of seedlings expressing Pro35S:HRE2-HA. **A)** Immunoblots of crude protein extracts from 6-d-old roots of the indicated genotypes, probed with anti-HA (α-HA) antiserum or antibodies specific for the hypoxia markers, ADH and PGB1 (the hypoxia marker antibodies were applied simultaneously to a single membrane). Ponceau S staining was used to confirm equal loading. **B)** Expression of *HRE2* transgene relative to Col-0. Values are means ± Sd (*n* = 4). **C)** Expression of *ADH* and *PGB1* relative to Col-0. Values are means ± Sd (*n* = 4). **D–G)** Molecular analysis of seedlings expressing Pro35S:RAP2.3-HA. **D, G)** Immunoblots of crude protein extracts from 6-d-old seedlings of the indicated genotypes, probed with anti-HA (α-HA) antiserum or antibodies specific for the hypoxia markers, ADH and PGB1. **E)** Expression of *RAP2.3* transgene relative to Col-0. Values are means ± Sd (*n* = 4). **F)** Expression of *ADH* and *PGB1* relative to Col-0. Values are means ± Sd (*n* = 4). **H–J)** Molecular analysis of seedlings expressing ProVRN2:VRN2-GUS. **H)** Histochemical staining of GUS reporter activity in 6-d-old seedlings. Seedlings were rearranged on an agar plate prior to photography. Bar: 1 cm. **I)** Immunoblot of 6-d-old seedlings probed with anti-GUS antibody. **J)** Expression of *VRN2* and *GUS* relative to Col-0. Values are means ± Sd (*n* = 4). For all plots, different letters indicate significant differences between conditions (*P* < 0.05; ANOVA with Tukey multiple comparison test).

To explore whether BIG influences the stability of physiological substrates, we focused on the PRT6/N-degron pathway, for which several targets have been identified. We first tested 2 representative ERFVII transcription factors by crossing plants expressing hemagglutinin (HA)-tagged HRE2 and RAP2.3 (Pro35S:HRE2-HA, Gibbs et al. 2011; Pro35S:RAP2.3, Gibbs et al. 2014) to N-degron pathway mutants and *big-2*. The protein abundance of HRE2-HA was increased in *prt6-5 big-2* roots relative to the single *prt6-5* mutant (Fig. 3A). Transgene-specific RT-qPCR showed that the increased levels of HRE2-HA protein were not driven by an increase in transcript abundance (Fig. 3B).

Consistent with the known role of ERFVIIs in inducing hypoxia-responsive gene expression, the enhanced HRE2-HA protein abundance in *prt6-5 big-2* relative to *prt6-5* was accompanied by a much stronger induction of the core hypoxia genes *ADH* and *PGB1* and the respective proteins (Fig. 3, A and C). RAP2.3-HA protein was stable in *prt6-5* seedlings, but not detectable in Col-0 and *big-2* (Fig. 3D), and hypoxia markers were strongly enhanced in the *prt6-5* line (Fig. 3, D and F). Transgene transcript levels of *RAP2.3* were lower in the *prt6-5* background compared with Col-0, indicating that the increased abundance of RAP2.3-HA is due to posttranscriptional regulation (Fig. 3E).

*prt6-5* seedlings expressing Pro35S:RAP2.3-HA had curled cotyledons with a defective cuticle and the true leaves developed more slowly (Supplementary Fig. S3, A and B); rosette development and flowering were also delayed (Supplementary Fig. S4, A and B). *prt6-5 big-2* double mutant lines expressing Pro35S:RAP2.3-HA exhibited curled leaves and generally stunted growth, and flowering was extremely delayed with only a short primary bolt produced (Supplementary Fig. S4B). We were unable to recover seeds from these plants; dissection of flowers revealed incompletely elongated stamens that did not mature or release pollen (Supplementary Fig. S4C). Therefore, we analyzed pooled seedlings homozygous for *prt6-5* but segregating for *big-2*. Accordingly, we observed a modest increase in RAP2.3-HA abundance and hypoxia marker expression, despite only 1 quarter of these seedlings being homozygous for both mutations (Fig. 3G).

We next tested whether the abundance of a functionally distinct endogenous N-degron pathway substrate, VRN2, was influenced by BIG, using a VRN2-GUS fusion driven by the native *VRN2* promoter (ProVRN2:VRN2-GUS; Gibbs et al. 2018). Histochemical staining confirmed previous results (Gibbs et al. 2018) with GUS present throughout the seedling in the *prt6-1* background, and additionally revealed increased intensity in *prt6-1 big-2* relative to *prt6-1* (Fig. 3H). Immunoblotting showed specifically that VRN2-GUS was increased in abundance in *prt6-1 big-2* compared with *prt6-1* and unstable in Col-0 and *big-2* (Fig. 3I). RT-qPCR demonstrated that there were no significant differences in *VRN2* or *GUS* expression between *prt6-1* and *prt6-1 big-2*, indicating that changes in VRN2-GUS abundance relate to posttranslational control by PRT6 and BIG (Fig. 3J).

### BIG works in parallel with PRT6 to regulate the hypoxia response

**Figure 4.**
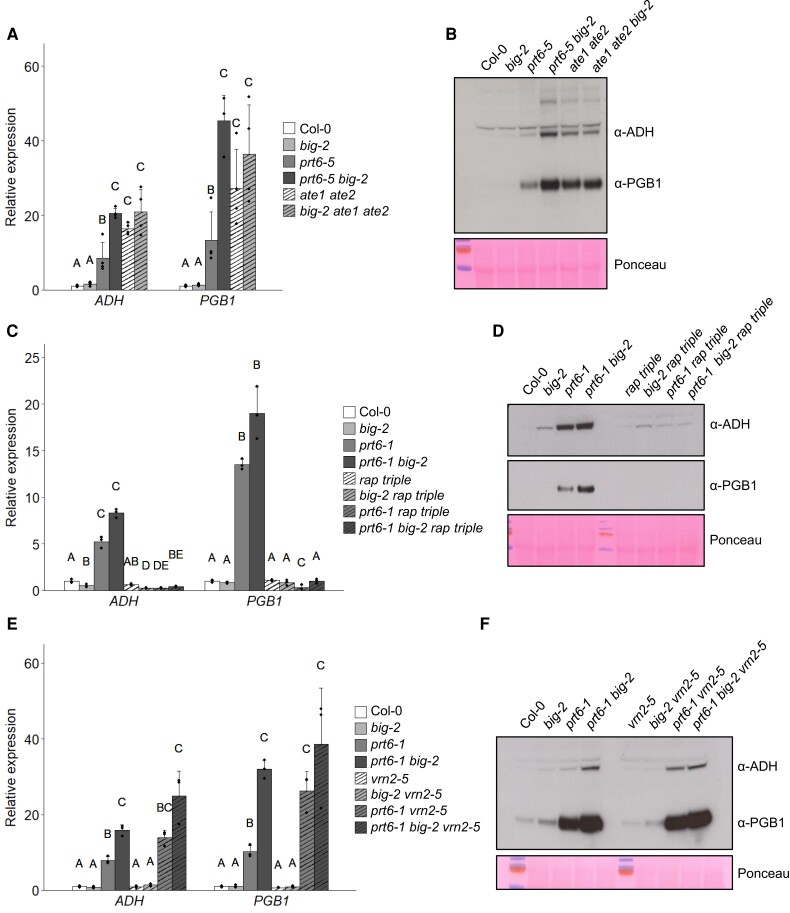
Regulation of the hypoxia response by BIG requires ATE1/2 and ERFVIIs. **A, B)** Molecular analysis of PRT6/N-degron pathway mutants. **A)** Expression of *ADH* and *PGB1* relative to Col-0 in 6-d-old seedlings. Values are means ± Sd (*n* = 4). **B)** Immunoblots of crude protein extracts from 6-d-old seedlings of the indicated genotypes, probed with antibodies specific for the hypoxia markers, ADH and PGB1 (which were applied to the same membrane). **C–F)** Molecular analysis of PRT6/N-degron pathway mutants combined with *rap2.12 rap2.2 rap2.3* mutant alleles (*rap triple*) **(C, D)** or with *vrn2-5***(E, F)**. **C**, **E)** Expression of *ADH* and *PGB1* relative to Col-0 in 6-d-old seedlings. Values are means ± Sd (*n* = 3). **D, F)** Immunoblot of crude protein extracts from 6-d-old seedlings of the indicated genotypes, probed with anti-HA (α-HA) antiserum or antibodies specific for the hypoxia markers, ADH and PGB1. Ponceau S staining was used to confirm equal loading. For all plots, different letters indicate significant differences between conditions (*P* < 0.05; ANOVA with Tukey multiple comparison test).

To further understand how BIG regulates the hypoxia response, we constructed a series of combination mutants using alleles lacking pathway substrates and enzymes, and then quantified hypoxia markers. Firstly, to observe whether arginylation is necessary for BIG to participate in the N-degron pathway, a mutant lacking arginyl transferase activity was crossed to *big-2*. Expression of *ADH* and *PGB1* and accumulation of the respective proteins were comparable in *ate1 ate2* and *ate1 ate2 big-2*, indicating that regulation of the hypoxia response by *BIG* is dependent on arginylation (Fig. 4, A and B). Genetic removal of *RAP2.12*, *RAP2.2*, and *RAP2.3* was sufficient to prevent constitutive expression of hypoxia markers in *prt6-1* seedlings, as shown previously (Zhang et al. 2018a), and also in the *prt6-1 big-2* background (Fig. 4, C and D), demonstrating that BIG influences hypoxia gene expression exclusively through RAP-type ERFVIIs. The mutants were also followed through development to determine whether regulation of ERVIIs underpins other phenotypes of *prt6-1 big-2*. Removal of RAP-type ERFVIIs did not have an impact on the overall morphology of *big-2*, consistent with the lack of stabilization in the single mutant. However, the stunted size and delayed flowering of *prt6-1 big-2* were partially rescued by the removal of these substrates (Supplementary Fig. S5).

Given that the increased stabilization of ERFVII transcription factors in *prt6-1 big-2* was associated with enhanced levels of key hypoxia-response genes and proteins (Figs. 3 and 4), we hypothesized that the double mutant might be more tolerant of hypoxia than *prt6*. Chlorophyll retention can be used as a marker of hypoxia tolerance, and we found that *prt6-1 big-2* and *prt6-5 big-2* seedlings had enhanced chlorophyll levels compared with the respective single mutants following hypoxia treatment, but similar seedling survival rates (Supplementary Fig. S6A–D). Ectopic expression of RAP2.3 in *prt6-5*, where *ADH* levels were markedly elevated, dramatically enhanced both chlorophyll content and survival of seedlings following hypoxia (Supplementary Fig. S6E–G), consistent with the role of RAP2.3 as a positive regulator of hypoxia responses. We also tested 2 further, distinct types of hypoxia responses: primary root regrowth after hypoxia treatment (in seedlings) and waterlogging tolerance (in mature plants). We did not observe reproducible tolerance of *prt6* or *prt6 big-2* mutants in root re-growth assays, but ∼20% of *prt6-5* roots expressing Pro35S:RAP2.3-HA re-grew after 4 h hypoxia (Supplementary Fig. S7A). Although *prt6* alleles exhibited waterlogging tolerance, *big-2* and *prt6 big-2* plants were sensitive to waterlogging, presumably due to their greatly reduced root systems. Interestingly, ectopic expression of RAP2.3-HA did not confer waterlogging tolerance under the conditions tested (Supplementary Fig. S7B).

VRN2 was not only first defined as a key regulator of vernalization, but also contributed to hypoxia stress survival, with the *prt6-1 vrn2-5* mutant exhibiting lower tolerance than *prt6-1* (Gibbs et al. 2018). However, *VRN2* was not required for hypoxia gene expression; indeed, expression of *PGB1* was increased in *prt6-1 vrn2-5* compared with *prt6-1* (Fig. 4, E and F), suggesting that VRN2 may suppress expression of some hypoxia-responsive genes under conditions where ERFVIIs are stabilized. Other hypoxia-responsive genes (*ADH1*, *ACC OXIDASE 1*, *HYPOXIA-RESPONSE ATTENUATOR1*, *HYPOXIA-RESPONSE UNKNOWN PROTEIN 40*, *PCO1*, *PCO2*, *PYRUVATE DECARBOXYLASE-2*) followed a similar trend but it was not statistically significant (Supplementary Fig. S8).

### The root transcriptome is extensively remodeled in *prt6-5 big-2* mutants

**Figure 5.**
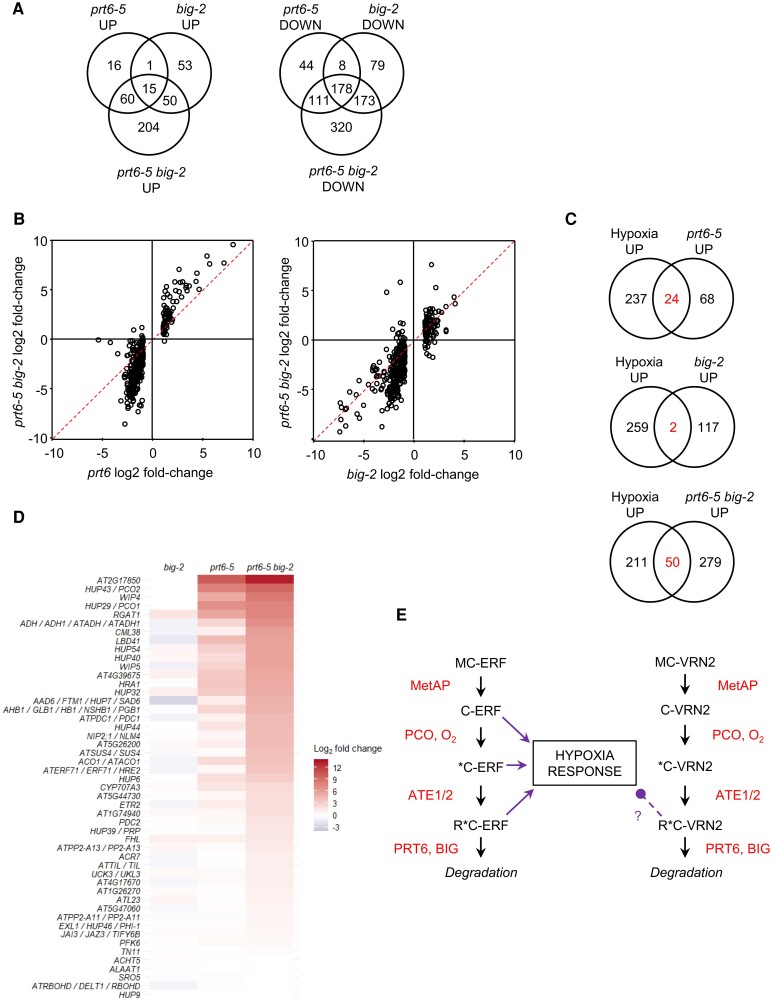
Impact of BIG on the root transcriptome. **A)** Numbers of DEGs in roots of different mutant backgrounds. DEGs are defined as having a fold change ≥2 at adjusted *P*-value <0.01. **B)** Plots comparing the fold change of transcripts in *big-2* and *prt6-5* single mutants with the double mutant, *prt6-5 big-2*. The dashed red line indicates an equal fold change in the 2 genotypes. **C)** Venn diagram showing overlap of DEGs with hypoxia-responsive genes in Arabidopsis roots (differentially regulated following 7 h of dark submergence; Lee et al. 2011). **D)** Heatmap showing log_2_-fold change of 49 “core hypoxia” genes (Mustroph et al. 2009) in the different mutant backgrounds relative to Col-0. Gene names or Arabidopsis Genome Initiative (AGI) codes are shown to the left of the panel. **E)** Scheme summarizing the impact of BIG on the N-degron pathway and the hypoxia response. Under normoxia, ERVII transcription factors and VRN2 are sequentially modified by MetAPs, PCOs, and arginyl-tRNA protein transferases (ATE1/2), such that the Nt Met is removed to reveal Cys2, which is oxidized (*C) and then arginylated (R*C). R*C-ERF and R*C-VRN2 are then targeted for degradation by PRT6 and also by a process involving BIG. Degradation is prevented in hypoxic conditions and in the absence of PRT6 (and BIG) function. The accumulation of ERFVIIs initiates the transcription of hypoxia-responsive genes. The accumulation of VRN2 negatively influences the expression of certain hypoxia-responsive genes.

To obtain further insight into the impact of *BIG* on the PRT6/N-degron pathway and potentially other processes, we conducted mRNA sequencing (RNA-seq) analysis of *big-2*, *prt6-5*, *prt6-5 big-2*, and Col-0 roots. One centimeter root sections containing the root tip were selected to minimize potential developmental effects associated with the small size of *big-2* seedlings. As principal component analysis indicated that samples clustered tightly by genotype (Supplementary Fig. S9A), we generated lists of differentially expressed genes (DEGs) for each mutant relative to wild type, with a cut-off fold change of 2 and adjusted *P*-value <0.01 (Supplementary Data Set 2). Analysis of DEGs identified 92 and 119 genes upregulated in *prt6-5* and *big-2*, respectively, with only 16 common DEGs. Three hundred and forty-one and 438 genes were downregulated in *prt6-5* and *big-2*, respectively, with 186 common to both data sets (Fig. 5A). Greater numbers of DEGs were identified in *prt6-5 big-2*, and in many cases, the fold changes of the common DEGs were markedly elevated in the double compared with the respective single mutants (Fig. 5B), indicative of a genetic interaction between *prt6-5* and *big-2*. There were notable overlaps between genes upregulated in *prt6-5* and *prt6-5 big-2* roots with previously published microarray data from *prt6* and *ate1/2* seedlings (Gibbs et al. 2011; de Marchi et al. 2016), but little overlap between *big-2* DEGs and the published data for *prt6* and *ate1/2* (Supplementary Fig. S9B–D).

Gene ontology term analysis (Ge et al. 2020) for “Biological Process” revealed that hypoxia-related terms were highly enriched in *prt6-5* and *prt6-5 big-2* upregulated genes, whereas “Photosynthesis,” “Glycolate and dicarboxylate metabolism,” and “Carbon metabolism” were enriched in *big-2* upregulated DEGs (Supplementary Fig. S10). All the 49 “core” genes known to be induced across cell types by hypoxia in wild-type plants (Mustroph et al. 2009) were present in the full transcriptome data set, with 21 upregulated in *prt6-5*. Comparison with transcriptome data from (Lee et al. 2011) revealed further hypoxia-responsive genes that are constitutively upregulated in *prt6-5* and *prt6-5 big-2* roots (Fig. 5C). In agreement with RT-qPCR and immunoblotting data (Fig. 4), the fold change in expression was markedly enhanced in *prt6-5 big-2* relative to the *prt6-5* single mutant (Fig. 5D), indicating an enhancement that is consistent with the increased stability of N-degron substrates such as the ERFVIIs (Fig. 5E). Six of the seven known N-degron pathway substrates were represented in the RNA-seq data set, among which only the hypoxia-responsive genes *HRE1* and *HRE2* were upregulated in *prt6-5 big-2* (Supplementary Fig. S9E).

### Suberin deposition is repressed in *prt6* and *big-2* roots

**Figure 6.**
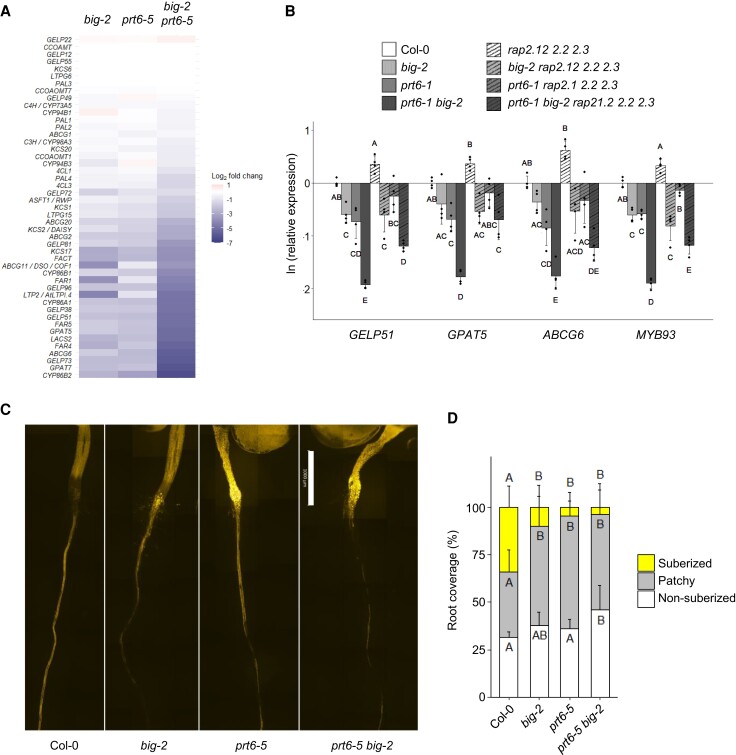
PRT6 and BIG regulate suberin deposition in roots. **A)** Heatmap derived from RNA-seq data showing log_2_-fold change of genes associated with suberin biosynthesis and deposition in the different mutant backgrounds, relative to Col-0. The gene list was curated from Mustroph and Bailey-Serres (2010), Ursache et al. (2021), and Serra and Geldner (2022). Gene names or AGI codes are shown to the left of the panel. **B)** RT-qPCR analysis of genes involved in suberin biosynthesis and deposition in 5-d-old roots of mutants, showing natural log of expression relative to Col-0. Values are means ± Sd (*n* = 4); different letters indicate significant differences between genotypes (*P* < 0.05). **C)** Representative composite micrographs showing Fluorol Yellow 088 staining of suberin in wild-type and mutant roots (scale bar represents 1 mm; images are scaled identically). The full root images are shown in Supplementary Fig. S8. **D)** Quantification of suberin deposition along the root axis using 3 different zones: nonsuberized, patchy, and continuous. Data are presented as mean percentage coverage of root length ± Sd (*n* = 10 roots; representative of 2 independent experiments); different letters indicate significant differences between genotypes for each region (*P* < 0.05; ANOVA with Tukey multiple comparison test).

Of the downregulated genes, “Glucosinolate biosynthetic process” was enriched in *prt6-5* and *prt6-5 big-2* DEGs, in agreement with previous findings for *ate1/2* (de Marchi et al. 2016), and “Cellular response to iron starvation” was enriched in *prt6-5* and *big-2* (Supplementary Fig. S11). Strikingly, however, the most enriched terms for downregulated genes in all genotypes were “Suberin biosynthetic process” and “Cutin biosynthetic process,” 2 pathways which share common components (Li-Beisson et al. 2013; Supplementary Data Set 2). Genes associated with suberin biosynthesis and transport were downregulated in both *big-2* and *prt6-5*, accounting for almost all the steps in the pathway. These include long-chain acyl-CoA synthetases, 3-ketoacyl-CoA synthetases, fatty acid reductases, fatty acid omega-hydroxylases, glycerol acyltransferases, feruloyl acyltransferase, fatty alcohol caffeoyl-CoA transferase, 4-coumarate-CoA ligase, ABC transporters, lipid transfer proteins and GDSL lipases (Li-Beisson et al. 2013; Serra and Geldner 2022). Fold changes of the differentially regulated genes were greater in *prt6-5 big-2*, compared with the respective single mutants (Fig. 6A; Supplementary Data Set 2).

Moreover, 6 MYB transcription factors (*MYB9*, *MYB41*, *MYB53*, *MYB52*, *MYB93*, and *MYB39/SUBERMAN*), which act in a hierarchical network to control suberin biosynthesis in Arabidopsis (Shukla et al. 2021; Xu et al. 2022), are also downregulated in the N-degron pathway mutants, with fold change increased in *prt6-5 big-2* relative to the single mutants (Supplementary Fig. S12A). In agreement with this, 45 of the 149 genes upregulated in *AtMYB41*-overexpressing plants (Cominelli et al. 2008) were downregulated in *prt6-5 big-2* (Supplementary Fig. S12B). RT-qPCR analysis showed that transcript levels of representative suberin genes were not significantly different between Col-0 and *prt6-1 rap2.12 rap2.2 rap2.3*, indicating that their repression in *prt6-1* roots requires RAP-type ERFVII transcription factors. In contrast, repression of suberin genes in *big-2* roots was ERFVII independent (Fig. 6B).

To explore the physiological significance of altered gene expression in the mutants, roots were stained with Fluorol Yellow 088. As *big-2* roots were significantly shorter than those of other genotypes (Supplementary Fig. S12C), suberization was expressed as a percentage of root length. The suberized zone was less extensive in *prt6-5*, *big-2*, and *prt6-5 big-2* roots than in wild-type Col-0 roots (Fig. 6, C and D; Supplementary Fig. S12D). Taken together, the results indicate that suberin deposition is constrained in PRT6/N-degron pathway mutant roots by ERFVII stabilization, and via an additional mechanism in *big-2*, pointing to shared and distinct roles for *BIG* and *PRT6* in control of this process.

Subsequent to the transcriptome study, resequencing of the *big-2* mutant revealed a second T-DNA inserted in the final exon of At3g61680 (*PLASTID LIPASE 1*; *PLIP1*) which encodes a plastid-localized phospholipase A1 involved in seed oil biosynthesis (Wang et al. 2017; Supplementary Fig. S1). We designated this allele *plip1-3* as 2 T-DNA mutants that have been reported previously (Wang et al. 2017). *BIG* and *PLIP1* are located at opposite ends of Chromosome 3, and consequently, in most cases, we were able to work with material in which the second T-DNA had been segregated out. An important exception is the RNA-seq analysis which unfortunately was performed with the seed homozygous for *plip1-3*. We consider it unlikely that a lesion in *PLIP1* would lead to the stabilization of N-degron pathway substrates. Nevertheless, to rule out the possibility that the lesion in *PLIP1* was causal for any of the *big-2* phenotypes reported in this paper, we repeated RT-qPCR analysis using material lacking *plip1-3*. This clearly demonstrated that the enhanced expression of hypoxia-responsive genes in *prt6-1 big-2* is independent of *plip1-3*, as is the altered expression of suberin genes (Supplementary Fig. S1^3^). While it is possible that *plip1-3* influences other transcript changes in *big-2* and *prt6-5 big-2*, we conclude that the key findings of the study (including those from the transcriptome data) are robust.

## Discussion

Despite the physiological and agronomic importance of the plant Arg/N-degron pathways, not all of the molecular components have yet been identified (Holdsworth et al. 2020). The PRT6 N-recognin influences the stability of Type 1 substrates such as those initiating with R that can be generated by the action of protein cleavage and/or ATEs (Garzón et al. 2007; Graciet et al. 2009; Gibbs et al. 2011; Licausi et al. 2011; White et al. 2017). Notably, however, incomplete stabilization of the model substrate R-GUS in *A. thaliana prt6* mutants (Garzón et al. 2007) and the more severe phenotype of *ate1 ate2* compared with *prt6* (Graciet et al. 2009) suggest the possibility of an additional N-recognin with specificity for PRT6/N-degrons. In this study, we provide several lines of evidence that the giant UBR box protein, BIG mediates turnover of proteins bearing Type 1 N-degrons in concert with the PRT6/N-degron pathway and show that this influences the molecular response to low oxygen in Arabidopsis. Moreover, we demonstrate that BIG also contributes to the turnover of proteins with Type 2 N-degrons via the PRT1/N-degron pathway.

This study utilized the *big-2* allele in which the T-DNA is inserted about halfway through the gene (Supplementary Fig. S1). As it has a severe morphological phenotype, we considered that *big-2* is likely to be a loss of function allele, and our RNA-seq data indicate that *big-2* expresses a truncated transcript, albeit at a much lower level than wild type (Supplementary Data Set 2). Although the truncated transcript could potentially produce a protein containing the UBR box, it is unclear whether the truncated protein would be correctly folded. Importantly, information from Drosophila and mammalian homologs of BIG indicates that a truncated protein is likely to be nonfunctional in the N-degron pathway because it lacks the hemi-RING E3 ligase domain (Barnsby-Greer et al. 2024). In theory, a mutant allele that produces a truncated protein could be a dominant negative but plants heterozygous for *big-2* look similar to wild type, suggesting that there is no dominant negative effect (Supplementary Fig. S1).

Using N-degron pathway reporters, we demonstrated enhanced stability of a model PRT6 substrate, R-GUS as well as increased abundance of R-LUC and physiological substrates, HRE2 and VRN2 in the *prt6-5 big-2* mutant relative to *prt6-5* (Figs. 1 and 3; Supplementary Fig. S2). The increased abundance of HRE2 and VRN2 was not driven by increased transcript, thus, while we cannot rule out increased translation, the data are consistent with increased stability in the double mutant background, as shown for R-GUS. It was not possible to test unequivocally whether RAP2.3 is similarly stabilized in *prt6-5 big-2* seedlings since double mutant plants expressing Pro35S:RAP2.3-HA did not set seed (Supplementary Fig. S4). However, this observation, together with the partial rescue of delayed flowering and reduced fertility of *prt6-5 big-2* plants by genetic removal of *RAP* function (Supplementary Fig. S4) strongly suggests that RAP2.3 is also hyperstabilized in the double mutant and that extreme stabilization of RAP-type ERFVII transcription factors is deleterious to growth and reproduction.

The vegetative phenotype of *prt6-5 big-2* lines expressing Pro35S:RAP2.3-HA resembles that of other plants in which N-degron pathway substrates are stabilized, including transgenics expressing N-terminally truncated RAP2.12 (Giuntoli et al. 2017) and mutiple *pco* mutants (Masson et al. 2019; Weits et al. 2023). Interestingly, constitutive expression of RAP2.3-HA in *prt6-5* did not have a profound effect on morphology in mature plants, but development was considerably delayed, and seedlings exhibited curled cotyledons with a slight cuticle defect (Supplementary Figs. S3 and S4). This aligns with the observation of Giuntoli et al. (2017) that, although the PRT6/N-degron pathway controls ERFVII stability throughout vegetative development, ERFVII-dependent transcriptional activation is attenuated with age.

Stabilization of PRT6/N-degron pathway substrates in *prt6-5 big-2* plants markedly amplified the transcriptional response to hypoxia, as evidenced by RT-qPCR, immunoblot, and especially RNA-seq analysis, and was accompanied by enhanced chlorophyll retention in seedlings following hypoxia treatment (Figs. 3–5; Supplementary Fig. S6). Additional approaches to explore low oxygen tolerance were explored; however, it was challenging to associate increased expression of hypoxia-responsive genes with hypoxia tolerance against the backdrop of a pleiotropic mutant phenotype. Primary root elongation and lateral root development are severely impaired in *big-2* (Ruegger et al. 1997; López-Bucio et al. 2005; Yamaguchi et al. 2007; Guo et al. 2013), and we ascribe the waterlogging sensitivity of *big-2* and *prt6 big-2* (Supplementary Fig. S7B) to their highly reduced root systems. Intriguingly, while overexpression of RAP2.3 in *prt6-5* conferred hypoxia tolerance to seedlings in both chlorophyll retention and root regrowth assays, mature plants were intolerant of waterlogging under the conditions used. This could be explained by the aforementioned age-dependent decline in the ability of ERFVIIs to modulate gene expression (Giuntoli et al. 2017). It is also possible that the small roots of these plants are unable to withstand longer periods of oxygen deprivation, whereas the acute stresses in the chlorophyll retention and root regrowth assays allow for a better comparison across genotypes.

Higher order combination mutants demonstrated that BIG and PRT6 control the hypoxia response in seedlings exclusively through RAP-type ERFVII transcription factors (Fig. 4, C and D). Interestingly, however, stabilization of the PRC2 subunit VRN2 in PRT6/N-degron pathway mutants negatively influenced expression of the hypoxia-responsive gene *PGB1* (Fig. 4, E and F). The mechanism by which this occurs remains to be explored but may involve methylation, given the known role of the PRC2 complex in epigenetic regulation. We did not explore the impact of enhanced VRN2 stabilization on flowering since the *big-2* allele is in the Col-0 background which does not require vernalization. Furthermore, ectopic expression of *VRN2* does not remove the requirement for vernalization in ecotypes that require prolonged winter to initiate flowering (Labandera et al. 2021).

RNA-seq analysis revealed that BIG and PRT6 not only play a role in the hypoxia response but also influence the expression of several other groups of genes, particularly a regulon associated with suberin biosynthesis. Suberin is a complex polymer that can act as a barrier to nutrients and gases, and which shows remarkable developmental plasticity in roots (Shukla et al. 2021). *big-2* and *prt6-5* had an independent, partially additive negative effect on transcript abundance (Fig. 6, A and B; Supplementary Fig. S12). In agreement with the lower expression of key *MYB* transcription factors and their downstream targets, suberin deposition was reduced in *big-2*, *prt6-5*, and *prt6-5 big-2* roots (Fig. 6, C and D). *RAP2.12*, *2.2*, and *2.3* were required for the repression of suberin biosynthetic genes in *prt6*, which is perhaps surprising given that limiting radial oxygen diffusion through suberin deposition is an adaptive response to waterlogging in wetland species (Ejiri et al. 2021). However, there are important temporal and developmental differences between wild-type plants experiencing hypoxia in the field and Arabidopsis roots grown on plates; negative regulation of suberization by ERFVIIs may be a feedback mechanism triggered by the sustained activation of the hypoxia response in N-degron pathway mutants.

Intriguingly, repression of suberin biosynthetic genes in the *big-2* single mutant was independent of ERFVII transcription factors (Fig. 6B), suggesting that BIG influences other factors that control suberin deposition independently of PRT6. Suberization is strongly influenced by hormones, including auxin, which is associated with the growth phenotype of *big* alleles (Gil et al. 2001; Yamaguchi et al. 2007; Guo et al. 2013; Yamaguchi and Komeda 2013) and which has complex effects on suberin synthesis and degradation in the endodermis in Arabidopsis (Cook et al. 2021; Ursache et al. 2021). It is tempting to speculate that dysregulation of auxin synthesis and transport underpin the reduced suberization in *big-2*; however, there was no significant enrichment in transcripts related to auxin signaling pathways in *big-2* roots, in contrast to a previous transcriptome analysis employing leaves of a different *big* allele (Bruggeman et al. 2020). While it is possible that there are tissue-specific differences in auxin-related gene expression that are not detected in the bulk root transcriptome, other mechanisms regulating suberin in *big-2* roots cannot yet be ruled out.

Taken together, our study provides evidence that BIG not only participates in the N-degron pathways, impacting different aspects of plant physiology, but also influences other processes. This raises interesting mechanistic questions regarding the operation of BIG in N-degron and possibly other proteostatic pathways. BIG contains numerous protein–protein interaction domains (Supplementary Fig. S1; Gil et al. 2001) providing a platform for interaction with diverse protein partners and substrates. Proximity labeling identified both PRT6 and BIG as potential R-TurboID-interacting proteins (Fig. 2C), suggesting that BIG (like PRT6) may bind Arg/N-degrons, although a mutually compatible hypothesis is that BIG exists in a complex with PRT6 (see below). Reporter experiments revealed that BIG also works in concert with the PRT1 E3 ligase to mediate the degradation of F-GUS (Fig. 1). Thus, BIG likely acts as an N-recognin for both Types 1 and 2 substrates. This is consistent with the domain structure of BIG. The mammalian N-recognins, UBR1 and UBR2 bind Type 1 substrates via the UBR box and Type 2 substrates at the Clp-S-like N-domain (Kim et al. 2021). BIG, UBR4, and PRT6 each contain UBR boxes but lack the N-domain (Tasaki et al. 2005, 2009; Garzón et al. 2007). Whereas the UBR box of PRT6 binds Type 1 degrons, the UBR box of UBR4 recognizes both Types 1 and 2 N-termini through a distinct mechanism (Kim et al. 2020, 2021, 2022; Jeong et al. 2023). PRT1 also lacks a ClpS-like domain and may recognize Type 2 substrates via a ZZ domain, which is also present in BIG (Stary et al. 2003).

An important question is whether BIG possesses intrinsic E3 ligase activity. Although BIG does not contain either a canonical E3 ligase RING or HECT domain, it shares with UBR4 a “hemi-RING” zinc finger, which serves as an affinity factor for the recruitment of E2 ubiquitin conjugating enzymes (Barnsby-Greer et al. 2024), strongly suggesting that it also an E3. Notably, however, neither R substrates nor F substrates were stabilized in the single *big-2* mutant, suggesting that PRT6 and PRT1 are the dominant N-recognins in planta, with BIG providing a lower level of substrate turnover that is only detectable in the absence of PRT6 and PRT1. Ultimately, it will only be possible to test this in a reconstituted system with purified PRT6/PRT1, BIG, and the respective E2 and E1 enzymes, which would undoubtedly be extremely challenging. An alternative scenario is that BIG may play a more general role to prevent the release of potentially toxic, partly degraded proteins from the proteasome; recognizing them via their neo-N-termini and contributing to the turnover of N-degron pathway substrates is a consequence of this (Besche et al. 2009). BIG may also participate in autophagic pathways as is the case for UBR4 (Tasaki et al. 2013).

**Figure 7.**
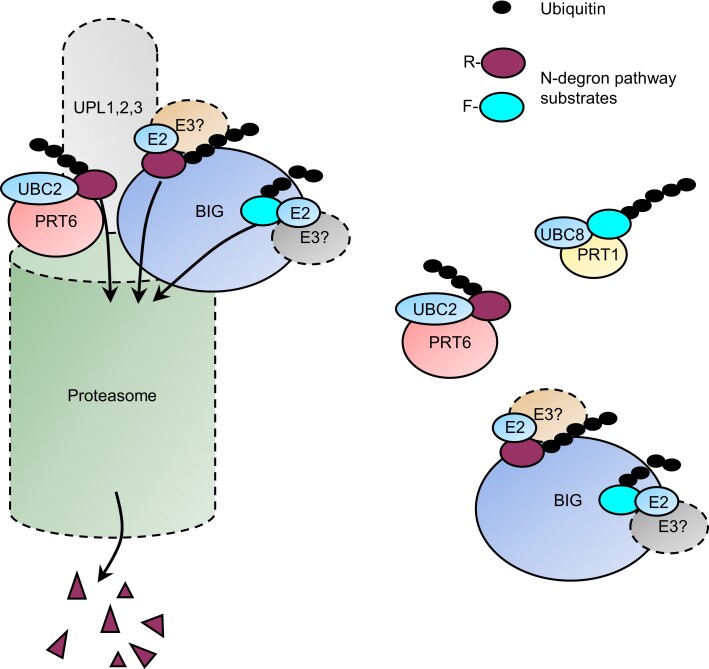
Speculative model for action of PRT6 and BIG. The cartoon is based on information from the literature and data from the current study. A subset of the cellular pool of BIG and PRT6 is bound to the proteasome lid where the 2 proteins may interact (Xie and Varshavsky 2000; Besche et al. 2009, 2014; Üstün et al. 2016; Fig. 2), but BIG and PRT6 likely also work without association with the proteasome, as indicated by the dashed lines around the proteasome and associated proteins. PRT6 is a candidate E3 ligase that works together with the E2 conjugating enzyme, UBC2 to ubiquitinate protein substrates with basic (Type 1) N-termini, targeting them for proteasomal degradation (Garzón et al. 2007; Kozlic et al. 2022). BIG acts as a scaffold, recruiting one or more as-yet unidentified E3 enzymes (indicated by different colors) as well as E2 enzymes to ubiquitinate protein substrates with Types 1 and 2 N-termini, resulting in their degradation (Ashton-Beaucage et al. 2016; Yau et al. 2017; Hunt et al. 2019; Fig. 1, E and F). Alternatively, BIG may have intrinsic E3 ligase activity, by analogy with UBR4 (Barnsby-Greer et al. 2024). The PRT1 E3 ligase also mediates the degradation of aromatic (Type 2) substrates, together with UBC8 (Stary et al. 2003; Mot et al. 2018). Several reports indicate that the HECT-type UPLs reside at the proteasome, where they increase the processivity of polyubiquitination in concert with multiple E3 ligases (Hwang et al. 2010; Wang and Spoel 2022).

Given that UBR4 interacts with a diverse array of protein partners, including E2 ubiquitin conjugating enzymes and E3 ligases to degrade both N-degron pathway substrates and other protein targets (Ashton-Beaucage et al. 2016; Yau et al. 2017; Hunt et al. 2019), it is plausible that BIG not only serves as a versatile recognition component of the Arg/N-degron pathways but also participates in other proteostatic mechanisms, interacting with one or more E3 ligases to mediate proteasomal degradation of a broad range of substrates (Fig. 7). Regulatory proteasome subunits and the HECT E3 ligases, ubiquitin protein ligase (UPL)1, UPL2, and UPL3 were enriched in R-TurboID samples (Fig. 2), which may indicate the presence of an N-recognin/E3 ligase complex at the proteasome. In agreement with this, BIG co-purified with proteasome subunits and UPL1/3 in transiently transfected *Nicotiana benthamiana* (Üstün et al. 2016). These observations are also consistent with previous reports of E3 ligases associated with the proteasome, including HECT E3 ligases (Wang and Spoel 2022) and yeast Ubr1 (Xie and Varshavsky 2000).

In yeast, the HECT E3 ligase Ufd4 binds Ubr1 and increases the processivity of polyubiquitination (Hwang et al. 2010); similarly, in plants, substrates from diverse E3 ligases, are relayed to UPLs which prevent substrate stalling at the proteasome (Wang and Spoel 2022). UBR4 is present at the proteasome at substoichiometric amounts in mammals (Besche et al. 2009, 2014) and not only co-purifies with UPL-type HECT E3 ligases but also regulates the proteolytic activity of the proteasome (Hunt et al. 2019, 2021). Collectively, this points to the existence of a proteostatic hub that is evolutionarily conserved but that has different interactors and substrates in plants and animals.

In conclusion, we have demonstrated that BIG participates in the Arg/N-degron pathways, contributing to the turnover of ERFVII transcription factors and VRN2 in the context of oxygen signaling and have shown that this does not underpin all of the known growth phenotypes associated with loss of *BIG* function. Key challenges for future work will be to identify additional substrates and E3 ligases associated with BIG and link them to its physiological functions.

## Materials and methods

### Plant material

All Arabidopsis (*A. thaliana*) genetic material used in this study is listed in Supplementary Table S1. This study utilizes *big-2* (SALK_045560; Supplementary Fig. S1; Kasajima et al. 2007; Ivanova et al. 2014). N-degron pathway mutants *prt6-5*, *prt6-1*, and *ate1 ate2* were crossed to *big-2* to generate the double mutants *prt6-5 big-2*, *prt6-1 big-2*, and *big-2 ate1 ate2* triple mutant. N-degron pathway mutant alleles expressing Pro35S:DHFR-Ub-X-GUS reporter lines (Garzón et al. 2007), ProUBQ3:X-LUC reporter lines (Worley et al. 1998; Graciet et al. 2010), and ProVRN2:VRN2:GUS (Gibbs et al. 2018) were crossed to *big-2* or *prt6-1 big-2*, and Pro35S:HRE2-HA in Col-0 (Gibbs et al. 2011) was crossed to *prt6-5 big-2* and segregated into different backgrounds. Pro35S:RAP2.3-HA in Col-0 (Gibbs et al. 2014) was crossed to *big-2* and *prt6-5*, respectively, the resultant lines were crossed to each other, and seeds were maintained as Pro35S:RAP2.3-HA *prt6-5−/− big+/−.* Higher order loss of function mutants was obtained by crossing *rap2.12 rap2.2 rap2.3*, *prt6-1 rap2.12 rap2.2 rap2.3* (Gibbs et al. 2014), and *vrn2-5 prt6-1* (Gibbs et al. 2018), to *big-2* and *prt6-1 big-2*. Where N-degron pathway reporters were compared in different genetic backgrounds, all lines were generated by crossing a specific transgenic line, such that all genotypes within a given experiment contain the same transgene event. All materials were validated by PCR- or CAPS-based genotyping. Details of primers are given in Supplementary Data Set 3. The *big-2* mutant was resequenced (∼25× raw coverage) by the Earlham Institute, using Low Input, Transposase Enabled library preparation and the Illumina NovaSeq 6000 S4 v1.5 platform. The positions of T-DNAs were identified by a BLAST search using the pBIN-pROK2 insertion sequences (http://signal.salk.edu/cgi-bin/tdnaexpress) as a query.

Constructs for proximity labeling are based on vector R4 GWB601 (Mair et al. 2019), obtained from Addgene, and transformed into Col-0. The amino acid sequence for R-Turbo (2548_HpaI_Turbo-NESYFP) is shown in Supplementary Fig. S1^4^, its M-Turbo counterpart differs by only 2 bases (exchange ATG for AGA, codon for first amino acid after ubiquitin cleavage).

R-LUC reporter lines in a wild-type Col-0 background (Graciet et al. 2010); based on constructs generated by Worley et al. (1998) were crossed with *prt6-5*, *big-2*, and *prt6-5 big-2* mutants. Lines containing the R-LUC reporter were selected on 0.5× Murashige and Skoog (MS) + 0.5% (w/v) sucrose, 0.8% (w/v) agar plates containing 20 mg/L Basta, and subsequently (i) genotyped to isolate homozygous mutants for *big-2* and *prt6-5*; and (ii) sequenced to confirm the identity of the R-LUC reporter.

### Growth of Arabidopsis

Seeds were raised from plants grown in Levington's F2S compost under long day conditions (16 h day/8 h night; 23/18 °C) light intensity of 250 *µ*mol photons m^−2^ s^−1^ (Sunlight replica NS1, Valoya); all genotypes to be compared were raised in the same controlled environment cabinet. Seeds were harvested, sieved (<425 *µ*m; Endecotts, London, UK) and stored at room temperature. After-ripened seeds were surface sterilized and sown on 0.5× MS medium containing 0.5% to 1% (w/v) sucrose and 0.8% (w/v) plant agar (Duchefa). After 2- to 3-d dark chilling at 4 °C, plates were grown in long day conditions (16/8 h; 22 °C) for 4- to 10-d light intensity of 150 *µ*mol photons m^−2^ s^−1^ (T5 54 W fluorescents, Sylvania).

### Genotyping

For DNA isolation, frozen tissue samples [1 to 2 leaves from soil-grown plants or ∼20 seedlings grown on 0.5× MS + 0.5% (w/v) sucrose and 0.8% (w/v) agar plates] were homogenized using a Geno/Grinder (1,750 rpm for 1.5 min) equipped with metal blocks prechilled with liquid N_2_. Five hundred microliters prewarmed cetyl trimethylammonium bromide buffer [2% (w/v) cetyl trimethylammonium bromide, 1% (w/v) polyvinyl pyrrolidone (MW = 40,000), 1.4 m NaCl, 0.1 m Tris HCl, 20 mm EDTA, pH 5.0] were added to the powder, and incubated at 60 °C for 30 min. Samples were centrifuged at 10,000 × *g* for 5 min, and 5 *µ*L RNase-A (10 mg/mL) were added to the supernatant and incubated for 15 min at room temperature. DNA was extracted by adding an equal volume of chloroform/isoamyl alcohol (24:1 v/v). Following centrifugation at 13,000*×g* for 1 min, DNA in the upper aqueous phase was precipitated by adding 0.7 volume of isopropanol and incubating at −20 °C for 15 min. DNA was pelleted by centrifugation at 13,000*×g* for 10 min, and the pellet was washed twice with 400 *µ*L prechilled 70% (v/v) ethanol and dried briefly, before dissolving in 100 *µ*L TE buffer (10 mm Tris, 1 mm EDTA, pH 8.0). PCR was performed using a 20 *µ*L total reaction volume, consisting of: 1× DreamTaq Green PCR Master Mix, 500 nm Forward Primer, 500 nm Reverse Primer, 10% (v/v) plant genomic DNA. Primers used are given in Supplementary Data Set 3.

### GUS staining

Six-d-old seedlings grown on 0.5× MS + 0.5% (w/v) sucrose and 0.8% (w/v) agar plates were immersed in 1 mL GUS assay buffer [100 mm sodium phosphate buffer (pH 7.0), 0.1% (v/v) Triton X-100, 0.5 mg/mL X-GlucA, 500 *µ*m potassium ferricyanide, 500 *µ*m potassium ferrocyanide] in sterile 24-well plates, vacuum infiltrated for 30 min in darkness, then wrapped in foil and incubated at 37 °C overnight. Chlorophyll was removed by incubation in 85% (v/v) ethanol, 15% (v/v) acetic acid with gentle agitation for 2 to 4 h until cleared, after which the seedlings were placed in sterile water. In each independent experiment, at least 10 seedlings were stained per genotype and 4 to 5 representative seedlings were arranged onto agar plates for photography.

### X-LUC assays

Seedlings stably expressing the R-LUC N-degron reporter construct were grown vertically on 0.5× MS + 0.5% (w/v) sucrose and 0.8% (w/v) agar plates containing 20 mg/L Basta to select for the presence of the reporter. Plates were kept at 4 °C in the dark for 3 days and then transferred to continuous light at 19.5 °C for 7 days (∼100 *µ*mol m^−2^ s^−1^ bulbs used: Philips 6,500 K T8 14.5 W). Forty seedlings per genotype and per biological replicate were harvested and immediately frozen in liquid nitrogen. Frozen tissue was ground using a drill and pestle, and the powder was split equally between 2 tubes for (i) LUC enzymatic assays and (ii) RNA extraction followed by RT-qPCR to normalize the LUC enzymatic activities to the expression of the *LUC* gene in each of the samples. Four biological replicates per genotype, each comprising 40 seedlings grown on separate plates were prepared and analyzed for both assays.

To test the enzymatic R-LUC activity, proteins were extracted from frozen ground tissue using 1× Luciferase Cell Culture Lysis Reagent (CCLR; Promega), supplemented with 1 mm phenylmethylsulfonyl fluoride and 1:100 plant Protease Inhibitor Cocktail (Sigma-Aldrich, St Louis, MO, USA). Samples were centrifuged at 12,000*×g* for 10 min at 4 °C to pellet cellular debris. Protein concentration was determined using the Bradford protein assay. Enzymatic LUC activity was measured as described in Graciet et al. (2010) and Luehrsen et al. (1992). Briefly, CCLR protein extract (1 *μ*L) was added to 100 *μ*L Luciferase Assay Reagent buffer (20 mm tricine, pH 7.8, 1.07 mm (MgCO_3_)_4_·Mg(OH)_2_·5H_2_O, 2.67 mm MgSO_4_, 0.1 mm EDTA acid, 33.3 mm DTT, 270 *μ*m coenzyme A, 470 *μ*M luciferin, and 530 *μ*M ATP in a 96-well plate (Sterilin). Luminescence was measured using a POLARstar Omega microplate reader (BMG LABTECH) for 10 s.

To determine expression levels of the R-LUC reporter, total RNA was extracted using the Spectrum Plant Total RNA Kit (Sigma-Aldrich/Merck), according to the manufacturer's instructions. Reverse transcription reactions were set up using 1,000 ng of total RNA, RevertAid Reverse Transcriptase (Thermo Fisher, Waltham, MA, USA) and associated buffer, RiboLock Rnase inhibitor (Thermo Fisher), and oligo(dT)18 and 1 mm dNTP mixture at 42 °C for 45 min. RT-qPCR reaction mixtures were prepared in LightCycler 480 96-well plates (Roche) with 1 *μ*L of cDNA, 1 *μ*L of primer pair mixture (1 *μ*m final concentration each primer; Supplementary Data Set 3), 5 *μ*L 2× SYBR green master mix (Roche), with nuclease-free water added to a final volume of 10 *μ*L per well. RT-qPCR reactions were carried out on a LightCycler 480 instrument (Roche). The second derivative maximum method was used to determine crossing point (Cp) values.

### Immunoblotting

Six-d-old roots or seedlings were harvested. Protein extraction and immunoblotting were performed as described in Zhang et al. (2018a), with the exception that 1% (w/v) BSA in phosphate buffered saline containing 0.1% (v/v) Tween-20 was used as the blocking agent in the case of the anti-Biotin blots. Briefly, proteins were separated in precast 4% to 12% (w/v) Bis-Tris gels using 1× SDS MES buffer and transferred to polyvinylidene fluoride using iBlot 2 Dry Blotting System (Thermo Fisher). Primary antibodies were used at the following dilutions: ADH (AS10685; Agrisera, Sweden), 1:3,000; PGB1 (raised in rabbit to full-length recombinant protein; Hartman et al. 2019) 1:3,000, GUS (G5420; Sigma-Aldrich), 1:1,000; HA (H 3663; Sigma) 1:1,000, and biotin (BN-34; Sigma) 1:2,000. The secondary antibodies used were antirabbit horseradish peroxidase conjugate (A0545; Sigma) diluted 1:50,000 (for ADH, PGB1 and GUS), m-IgGk BP-HRP (sc-516102; Santa Cruz Biotechnology) diluted 1:15,000 (for HA), or antimouse IgG-HRP (NA931; GE Healthcare) diluted 1:10,000 (for biotin). Blots were then washed and developed with SuperSignal West Pico PLUS Chemiluminescent Substrate (Thermo Fisher Scientific).

### Reverse transcription qPCR

Six-d-old roots or seedlings were harvested, frozen in liquid nitrogen, and homogenized using the Geno/Grinder as described for “Genotyping.” Total RNA was extracted using an RNeasy Plant Mini Kit (Qiagen) and treated using a TURBO DNA-free Kit (Invitrogen), or a Monarch Total RNA Miniprep Kit (New England Biolabs, Inc), with on-column DNAse I treatment. A RevertAid First Strand cDNA Synthesis Kit (Thermo Scientific) and anchored -oligo(dT)18 were used for cDNA synthesis for a 2-step RT-PCR. SYBR Green JumpStart Taq ReadyMix was used for real-time PCR using a Lightcycler 96 Instrument (Roche) or a Quantstudio 6 Pro (Thermo), according to the manufacturers' instructions. Three to 4 biological replicates, each consisting of 20 seedlings or 100 primary roots grown on separate plates, were included for each genotype. Two technical replicates were prepared per cDNA sample and primer combination. Relative quantification was performed using both *ACTIN 2* (*ACT2*; At3g18780.2) and *TUBULIN BETA CHAIN 4* (*TUB4*; At5g44340.1) as references. For the experiments presented in Fig. 4, C and E and Supplementary Fig. S8, a single reference gene (*ACT2*) was used due to practical constraints. *POLYUBIQUITIN10* (*UBQ10*; At4g05320.2) and At5g18800 were used as references for the experiments presented in Fig. 6B and Supplementary Fig. S13B. Relative gene expression was calculated using the 2^−ΔΔCt^ method (Livak and Schmittgen 2001), using the threshold cycles automatically determined by the software to obtain fold-change values, which were then normalized to the mean fold change of Col-0. Data were naturally log transformed for statistical analysis and visualization when indicated, as appropriate by linear modeling. Primers used are given in Supplementary Data Set 3.

### Hypoxia and waterlogging assays

Hypoxic conditions were imposed by anaero atmosphere generation bags (68,061 Sigma) in an anaerobic jar (28,029 Sigma), according to the manufacturer's instructions. Seedlings were grown on 0.5× MS + 0.5% (w/v) sucrose plus 0.8% (w/v) plant agar, and treated with hypoxia in the dark by enclosing the plates in an anaerobic jar, from which oxygen was reduced to below 1% within 1 h, monitored by smart sensor Oxygen Detector AR8100. Controls were kept in the dark for the same period of time under normal oxygen conditions. For chlorophyll measurement, 4-d-old seedlings were treated for 5 h, then returned to the light for 3-d recovery, seedlings were photographed, weighed, and submerged in 80% (v/v) acetone overnight at 4 °C, in darkness. Absorbance at 646 and 663 nm was used to estimate total chlorophyll (Lichtenthaler and Wellburn 1983). For survival scoring, seedlings were assigned a score based on their appearance as in Gibbs et al. (2011): 1 for no remaining chlorophyll, 3 for partial chlorophyll coverage, and 5 for complete chlorophyll coverage. Scores were aggregated to produce a mean survival score for each plate containing 20 to 30 seedlings. For the root regrowth assay, 7 seeds per genotype were sown on the same plates; 4 different configurations of plates were prepared, varying which position each genotype occupied, with 3 replicates per configuration. Therefore, there were 12 plates considered as biological replicates per treatment. Five-d-old seedlings were treated for 4 h, then the plates were turned 90° and photographed after 2-d recovery in the light. Regrowth, as indicated by bending of the primary roots, was scored. Waterlogging tolerance was assayed as described in Gibbs et al. (2018).

### Suberin quantification

Seeds were surface sterilized and plated on 0.5× MS + 0.5% (w/v) sucrose containing 0.8% (w/v) agar. After 2- to 3-d dark chilling at 4 °C, seedlings were grown vertically in long days (16/8 h; 22 °C) for 5 d and stained with Fluorol Yellow 088, as described in Barberon et al. (2016). Tiled images were captured across whole seedlings using a Zeiss Axio Imager.Z2 microscope (10× objective and GFP fluorescence filters: excitation 450 to 490 nm; emission 500 to 550 nm, illumination 450 to 488 nm) and Zen3.0 blue edition software. Seedlings were initially viewed using brightfield imaging at a low-light level to define the region to be scanned and create focal points (“support points”) along the length of the root. Entire seedlings were then scanned using fluorescence contrast imaging with 100% light intensity (excitation 488 nm; emission 509 nm) and a 10% overlap of tile images for alignment and stitching. Images were pseudo-colored using the “YellowToWhite” LUT, annotated with a 1,000 *µ*m scale bar, and exported as TIFF files at 70% of the original size. Suberization patterns were quantified using ImageJ to measure the length of the different regions in *µ*m: “suberized” for continuous suberization, “patchy” for partial suberization, and “nonsuberized” for the region with no suberized cells. Results were expressed as the percentage of the total root length.

### Cuticle staining

Cuticular integrity was assessed by Toluidine Blue staining, according to Tanaka et al. (2004). Briefly, 8-d-old seedlings grown on 0.5× MS + 1% (w/v) sucrose + 0.8% (w/v) agar plates were immersed in an aqueous solution of 0.05% (w/v) Toluidine Blue O (Sigma-Aldrich) for 2 min, rinsed twice with water, and arranged onto agar plates for photography.

### Statistical analysis

Statistical analyses were performed on comparable sets of data using the R environment and are presented in Supplementary Data Set 4. For analysis of multiple genotypes, transgenic lines, and/or treatments, base R was used to perform ANOVA, and the R packages “emmeans,” “predictmeans,” and “multcomp” were used for subsequent Tukey multiple comparisons tests. All packages are available from CRAN (https://cran.r-project.org/). Prior to statistical testing, data were log transformed if indicated as necessary by linear model fitting. All statistical tests performed were 2-sided.

Bar plots presented in this paper display mean values with individual data points overlaid, and error bars indicate Sd. Unique letters indicate statistically significant differences between groups (*P* < 0.05). Box plots display the median as the center line, the upper and lower quartiles as the box limits, 1.5× the interquartile range as whiskers, and individual data points are overlaid.

### RNA-seq

After-ripened seeds were surface sterilized and plated on nylon mesh (Sefar NITEX, 03-110/47; Heiden, Switzerland) overlaid on 0.5× MS medium containing 0.5% (w/v) sucrose and 0.8% (w/v) plant agar in square plates (688161, Greiner). One centimeter sections containing the root tip were harvested from 5-d-old seedlings, frozen in liquid nitrogen, and RNA was extracted using an RNeasy Plant Mini Kit (Qiagen) and treated using a TURBO DNA-free Kit (Invitrogen). Five biological replicates per genotype were prepared; an individual plate comprised a biological replicate. RNA-seq and data analysis were done using Illumina HiSeq (2 × 150 paired end reads) by Genewiz. Briefly, sequence reads were trimmed to remove possible adapter sequences and nucleotides with poor quality using Trimmomatic v.0.36. The trimmed reads were mapped to the *A. thaliana* TAIR10 reference genome available on ENSEMBL using the STAR aligner v.2.5.2b. Unique gene hit counts were calculated by using feature Counts from the Subread package v.1.5.2. Only unique reads that fell within exon regions were counted. Since a strand-specific library preparation was performed, the reads were strand-specifically counted. Differential expression analysis was performed using DESeq2. The Wald test was used to generate *P*-values and log_2_-fold changes. Genes with an adjusted *P*-value <0.05 and absolute log_2_-fold change >1 were called as DEGs for each comparison.

### Proximity labeling

Arabidopsis plants (15 seedlings per well, 24-well plate) were grown in liquid culture [1 mL 1 × MS medium with 1% (w/v) sucrose per well] for 1 week under long day conditions (23 °C, 16 h light from cool white fluorescent bulbs). Medium was exchanged with medium supplemented with 10 *µ*m Bortezomib and 50 *µ*m biotin 75 min before harvest. Plants were washed 4× with 2 mL ice-cold water, then 1 mL ice-cold water was added before they were dried and snap frozen in liquid N_2_. Tissue was homogenized in a precooled Tissue Lyser (2 × 10 min, 28 Hz) and 170 *µ*L extraction buffer [50 mm Tris pH 7.5; 150 mm NaCl; 1 mm EDTA; 0.5% (v/v) NP-40 substitute; 3 mm DTT; plant protease inhibitor cocktail (Sigma)] added. Extracts from 3 wells were pooled (= ∼500 *µ*L crude extract as input). After centrifugation at 4 °C, 500 *µ*L of the supernatant was loaded onto a Sephadex G-25 column (Cytiva MiniTrap PD-10) and eluted with 1 mL extraction buffer to remove free biotin. The protein concentration of the eluate was determined by Bradford assay, and amounts of extracts were adjusted to the sample with the lowest protein concentration (2.3 mg total protein). Samples were incubated with Pierce Magnetic Streptavidin beads equilibrated in extraction buffer (25 *µ*L beads per sample) for 60 min with rotation at 4 °C. The beads were washed 4× with 1 mL wash buffer (20 mm Tris pH 7.5; 500 mm NaCl; 0.5 mm EDTA), transferred to a fresh low binding tube, washed again with 1 mL wash buffer and finally resuspended in 500 *µ*L wash buffer. Three technical replicates per genotype were submitted to proteomic analysis. Two additional biological replicates (each including technical replicates) gave similar results.

### Sample preparation for MS analysis

The beads from the proximity labeling reactions were resuspended in 50 *µ*L 1 m urea, 50 mm ammonium bicarbonate. Disulfide bonds were reduced with 2 *µ*L of 250 mm DTT for 30 min at room temperature before adding 2 *µ*L of 500 mm iodoacetamide and incubating for 30 min at room temperature in the dark. The remaining iodoacetamide was quenched with 1 *µ*L of 250 mm DTT for 10 min. Proteins were digested with 150 ng LysC (MS grade; FUJIFILM Wako Chemicals) in 1.5 *µ*L 50 mm ammonium bicarbonate at 25 °C overnight. The supernatant without beads was digested with 150 ng trypsin (Trypsin Gold, Promega) in 1.5 *µ*L 50 mm ammonium bicarbonate followed by incubation at 37 °C for 5 h. The digest was stopped by the addition of trifluoroacetic acid to a final concentration of 0.5% (w/v), and the peptides were desalted using C18 Stagetips (Rappsilber et al. 2007).

### Liquid chromatography–MS analysis

Peptides were separated on an Ultimate 3000 RSLC nano-flow chromatography system (Thermo Fisher), using a precolumn for sample loading (Acclaim PepMap C18, 2 cm × 0.1 mm, 5 *μ*m; Thermo Fisher), and a C18 analytical column (Acclaim PepMap C18, 50 cm × 0.75 mm, 2 *μ*m; Thermo Fisher), applying a segmented linear gradient from 2% to 35% and finally 80% (v/v) solvent B [80% (v/v) acetonitrile, 0.1% (v/v) formic acid; Solvent A 0.1% (v/v) formic acid] at a flow rate of 230 nL/min over 120 min.

Eluting peptides were analyzed on an Exploris 480 Orbitrap mass spectrometer (Thermo Fisher) coupled to the column with a FAIMS pro ion-source (Thermo Fisher) using coated emitter tips (PepSep, MSWil) with the following settings: the mass spectrometer was operated in DDA mode with 2 FAIMS compensation voltages (CVs) set to −45 or −60 and 1.5 s cycle time per CV. The survey scans were obtained in a mass range of 350 to 1,500 *m*/*z*, at a resolution of 60k at 200 *m*/*z*, and a normalized automatic gain control (AGC) target of 100%. The most intense ions were selected with an isolation width of 1.2 *m*/*z*, fragmented in the higher-energy collisional dissociation cell at 28% collision energy, and the spectra recorded for maximum 100 ms at a normalized AGC target of 100% and a resolution of 15k. Peptides with a charge of +2 to +6 were included for fragmentation, the peptide match feature was set to preferred, the exclude isotope feature was enabled, and selected precursors were dynamically excluded from repeated sampling for 45 s.

### Proteomics data analysis

MS raw data split for each CV using FreeStyle 1.7 (Thermo Fisher) were analyzed using the MaxQuant software package (version 2.1.0.0; Tyanova et al. 2016) with the Uniprot *A. thaliana* reference proteome (version 2022.01; www.uniprot.org), target sequences, as well as a database of the most common contaminants. The search was performed with full trypsin specificity and a maximum of 2 missed cleavages at a protein and peptide spectrum match false discovery rate of 1%. Carbamidomethylation of cysteine residues was set as fixed, oxidation of methionine, and N-terminal acetylation as variable modifications. For label-free quantification (LFQ), the “match between runs” only within the sample batch and the LFQ function were activated—all other parameters were left at default. MaxQuant output tables were further processed in R 4.2.1 (https://www.R-project.org) using Cassiopeia_LFQ (https://github.com/moritzmadern/Cassiopeia_LFQ). Reverse database identifications, contaminant proteins, protein groups identified only by a modified peptide, protein groups with <2 quantitative values in 1 experimental group, and protein groups with <2 razor peptides were removed for further analysis. Missing values were replaced by randomly drawing data points from a normal distribution model on the whole dataset (data mean shifted by −1.8 Sds, a width of the distribution of 0.3 Sds). Differences between groups were statistically evaluated using the LIMMA 3.52.1 (Ritchie et al. 2015) at 5% false discovery rate (Benjamini–Hochberg).

### Accession numbers

Sequence data from this article can be found in the EMBL/GenBank data libraries under accession numbers: AT3G24800, AT5G02310, AT3G02260, AT5G05700, AT3G11240, AT1G53910, AT3G14230, AT3G16770, AT2G47520, AT4G16845, AT1G55860, AT1G70320, AT4G38600, AT5G39890, AT5G15120, AT4G33070, AT4G27450, AT4G24110, AT4G10270, AT3G23150, AT3G02550, AT3G10040, AT1G19530, AT1G43800, AT1G33055, AT2G16060, AT2G17850, AT1G77120, AT2G19590, AT4G17670, AT1G35140, AT1G26270, AT5G26200, AT5G66985, AT5G62520, AT5G61440, AT5G58070, AT5G54960, AT5G47910, AT5G47060, AT5G45340, AT5G44730, AT5G42200, AT5G10040, AT5G02200, AT3G61060, AT3G43190, AT4G39675, AT4G33560, AT4G32840, AT4G22780, AT3G27220, AT3G23170, AT3G17860, AT1G63090, AT1G76650, AT1G17290, AT1G74940, AT1G72940, AT1G55810, AT2G34390, AT1G51680, AT1G65060, AT2G39350, AT1G17840, AT2G37360, AT3G53510, AT5G13580, AT5G41040, At2g40890, AT2G30490, AT1G67990, AT4G34050, AT4G26220, AT5G58860, AT5G23190, AT5G08250, AT5G63450, AT3G48520, AT5G63560, AT5G22500, AT3G44540, AT3G44550, AT1G28650, AT1G54000, AT1G74460, AT2G19050, AT2G23540, AT2G30310, AT3G48460, AT3G50400, AT4G26790, AT5G37690, AT3G11430, AT5G06090, AT1G01120, AT4G34510, AT1G04220, AT5G43760, AT1G68530, AT1G49430, AT2G38530, AT2G48130, AT1G55260, AT2G37040, AT3G53260, AT5G04230, AT3G10340, and AT3G61680.

Data underlying this article are available and have been deposited in the following repositories: Genome resequencing data are deposited in the NCBI Sequence Read Archive, accession number PRJNA1046295. The RNA-seq data files for this study have been uploaded to NCBI (https://www.ncbi.nlm.nih.gov/) under project number PRJNA975350, with accession numbers SAMN35345055 to SAMN35345074. The MS proteomics data have been deposited with the ProteomeXchange Consortium via the PRIDE (Perez-Riverol et al. 2022) partner repository with the dataset identifier PXD041610.

## Supplementary Material

koae117_Supplementary_Data

## Supplementary data

The following materials are available in the online version of this article.

**Supplementary Figure S1.** Details of the *big-2* allele.

**Supplementary Figure S2.** BIG influences the stability of a model Type 1 Arg/N-degron pathway substrate.

**Supplementary Figure S3.** Morphology of seedlings ectopically expressing RAP2.3-HA.

**Supplementary Figure S4.** Morphology of plants ectopically expressing RAP2.3-HA.

**Supplementary Figure S5.** Morphology of PRT6/N-degron pathway and *erfvii* combination mutants.

**Supplementary Figure S6.** Hypoxia response of PRT6/N-degron pathway mutants.

**Supplementary Figure S7.** Response of PRT6/N-degron pathway mutants to hypoxia and waterlogging.

**Supplementary Figure S8.** Relative expression of hypoxia-responsive genes in N-degron pathway mutants.

**Supplementary Figure S9.** Transcriptome analysis of N-degron pathway mutants.

**Supplementary Figure S10.** Gene ontology term enrichment in genes upregulated in roots of N-degron pathway mutants.

**Supplementary Figure S11.** Gene ontology term enrichment in genes downregulated in roots of N-degron pathway mutants.

**Supplementary Figure S12.** Suberin biosynthesis and deposition in N-degron pathway mutants.

**Supplementary Figure S13.** Relative expression of hypoxia and suberin biosynthesis and deposition genes in *big-2* combination mutants lacking the *plip1-3* T-DNA.

**Supplementary Figure S14.** Amino acid sequence of R-Turbo and M-Turbo.

**Supplementary Table S1.** Genetic materials used in this study.

**Supplementary Data Set 1.** TurboID proteomics data.

**Supplementary Data Set 2.** RNA-seq data.

**Supplementary Data Set 3.** Primers used in this study.

**Supplementary Data Set 4.** Details of statistical analysis.

## Dive Curated Terms

The following phenotypic, genotypic, and functional terms are of significance to the work described in this paper:

upl1 Gramene: AT1G55860
upl1 Araport: AT1G55860

UPL2 Gramene: AT1G70320
UPL2 Araport: AT1G70320

ZPR2 Gramene: AT3G60890
ZPR2 Araport: AT3G60890

PRT6 Gramene: At5g02310
PRT6 Araport: At5g02310

adh1 Gramene: AT1G77120
adh1 Araport: AT1G77120

MYB52 Gramene: AT1G17950
MYB52 Araport: AT1G17950

TUB4 Gramene: AT5G44340
TUB4 Araport: AT5G44340

BIG Gramene: AT3G02260
BIG Araport: AT3G02260

ACT2 Gramene: AT3G18780
ACT2 Araport: AT3G18780

UBQ10 Gramene: AT4G05320
UBQ10 Araport: AT4G05320

HRE2 Gramene: AT2G47520
HRE2 Araport: AT2G47520

PLIP1 Gramene: At3g61680
PLIP1 Araport: At3g61680
