# Supplementary material for: BIG enhances Arg/N-degron pathway-mediated protein degradation to regulate Arabidopsis hypoxia responses and suberin deposition
Source: Plant Cell. 2024 Apr 12;36(9):3177–200. doi: 10.1093/plcell/koae117 (PMC11371152; doi:10.1093/plcell/koae117)
Supplement: koae117_Supplementary_Data [file koae117_supplementary_data.zip › Supplementary data.pdf]

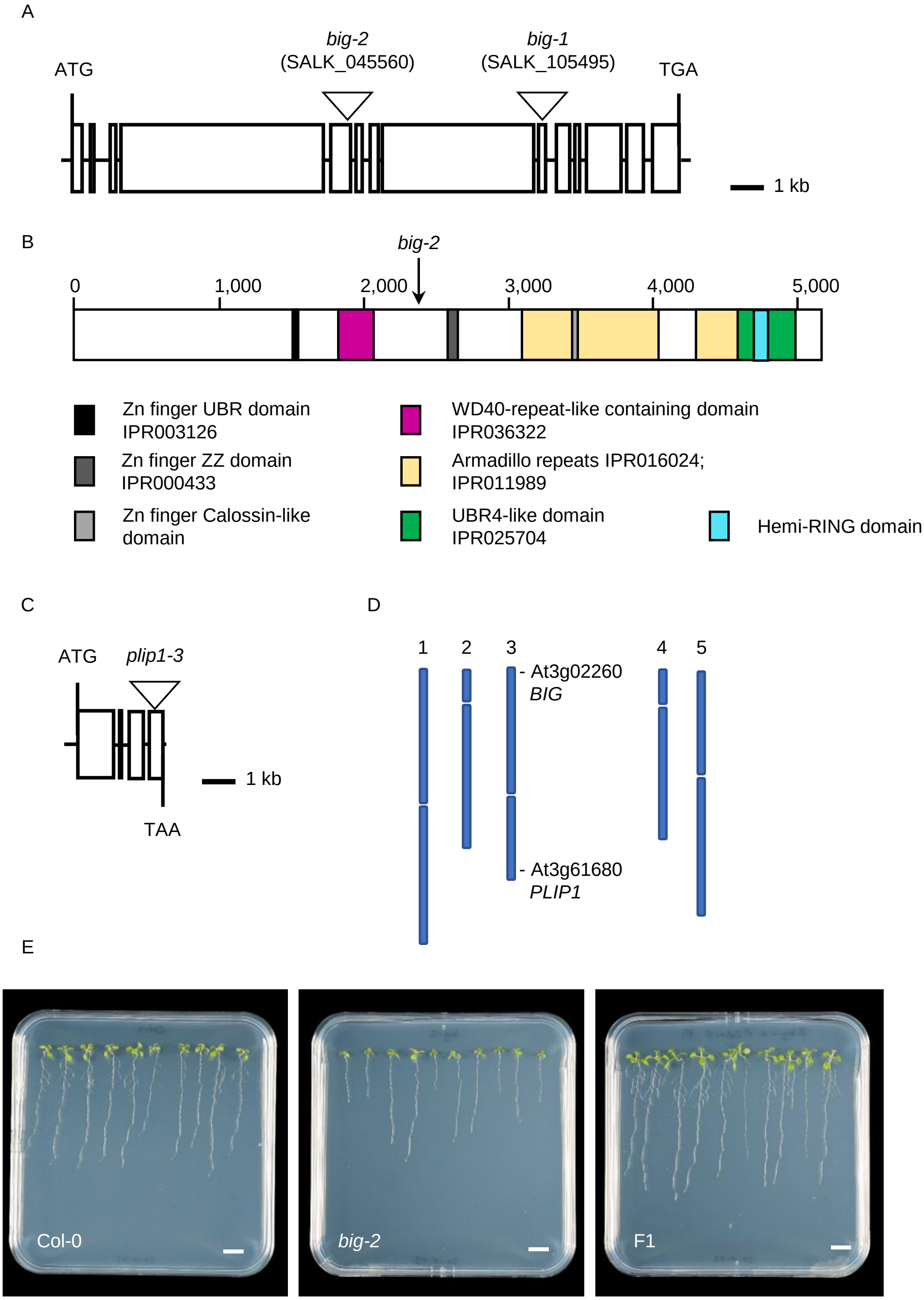

Supplementary Figure S1. Details of the *big-2* allele. Supports Fig. 1

(A) Genomic structure of *BIG* (At3g02260), showing positions of two T-DNA insertions originally described in (Kasajima et al., 2007); note that *big-1* and *big-2* are mis-labelled in some later publications. Intron/exon positions are as in the corrected gene model from (He et al., 2018). (B) Schematic of BIG showing positions of predicted protein domains, as indicated in (Gil et al., 2001; Barnsby-Greer et al., 2024) and TAIR. The arrow shows the position of the stop codon in *big-2* and the scale shows number of amino acids. (C) Detail of a second T-DNA in the original SALK_045560 stock, inserted in *PLASTID LIPASE 1* (*PLIP1*; At3g61680) after T2021 in the genomic sequence (T1674 in the cDNA). This allele was designated *plip1-3*. (D) Chromosomal location of *BIG* and *PLIP1.* (E) Morphology of 10-d old seedlings. Col-0, *big-2* and F1 progeny of a *big-2* x Col-0 cross were grown under long days on 0.5 x MS medium containing 0.5 % (w/v) sucrose. Bar = 1 cm.

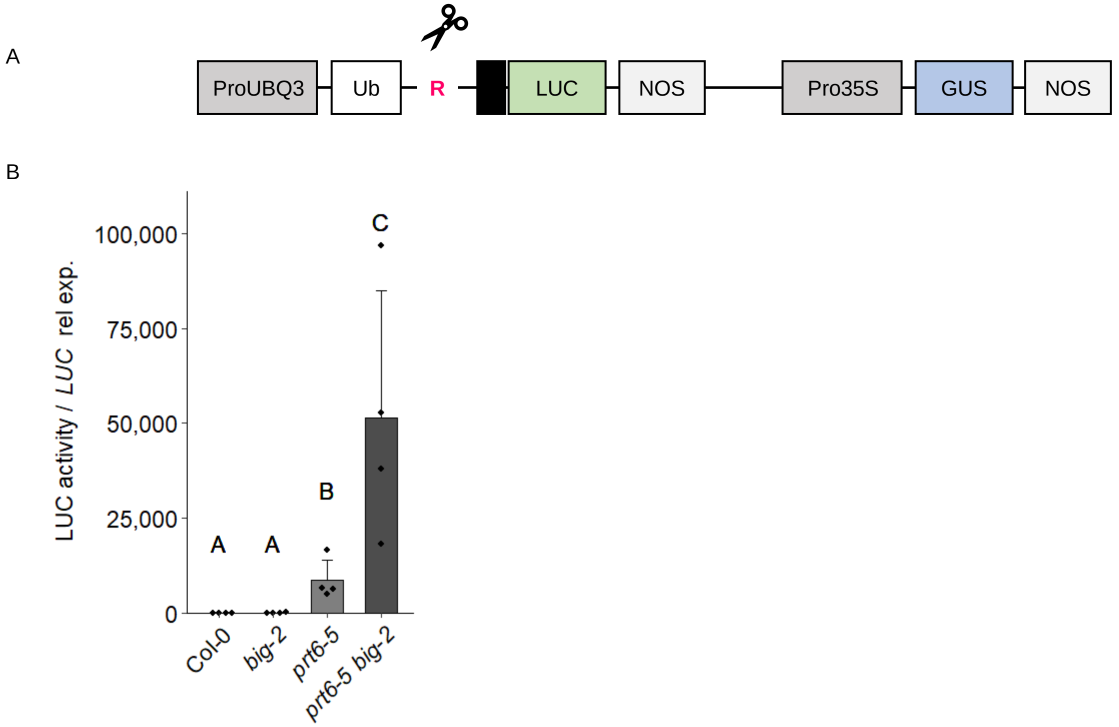

Supplementary Figure S2. BIG influences the stability of a model type 1 Arg/N-degron pathway substrate. Supports Figure 1

(A) Alternative system for generation of N-degron pathway substrates (Worley et al., 1998). A fusion of Ubiquitin (Ub; white) to *LUCIFERASE* (LUC, green) is driven by the *POLYUBIQUITIN 3* promoter (ProUBQ3, grey). A short linker (represented by a black rectangle) is present between the variant residue, in this case, R, and the LUC reporter. Co-translational cleavage by ubiquitin-specific proteases reveals R as the neo-N-terminus in planta. In the same construct, *GLUCURONIDASE* (GUS; blue) driven by the *CaMV35S* promoter (Pro35S) acts as a stable reference protein. NOS: nopaline synthase transcriptional terminator sequence. (B) Luciferase activity in 7-d old seedlings of different genotypes expressing R-LUC. Luciferase activity was normalised according to *LUC* transcript levels because the *GUS* normalising cassette was variably silenced during crossing, probably due to the presence of multiple copies of the *CaMV35S* promoter in the mutant backgrounds. Values are means ± SD (n=4); different letters indicate significant differences between conditions ($P < 0.05$; ANOVA with Tukey multiple comparison test).

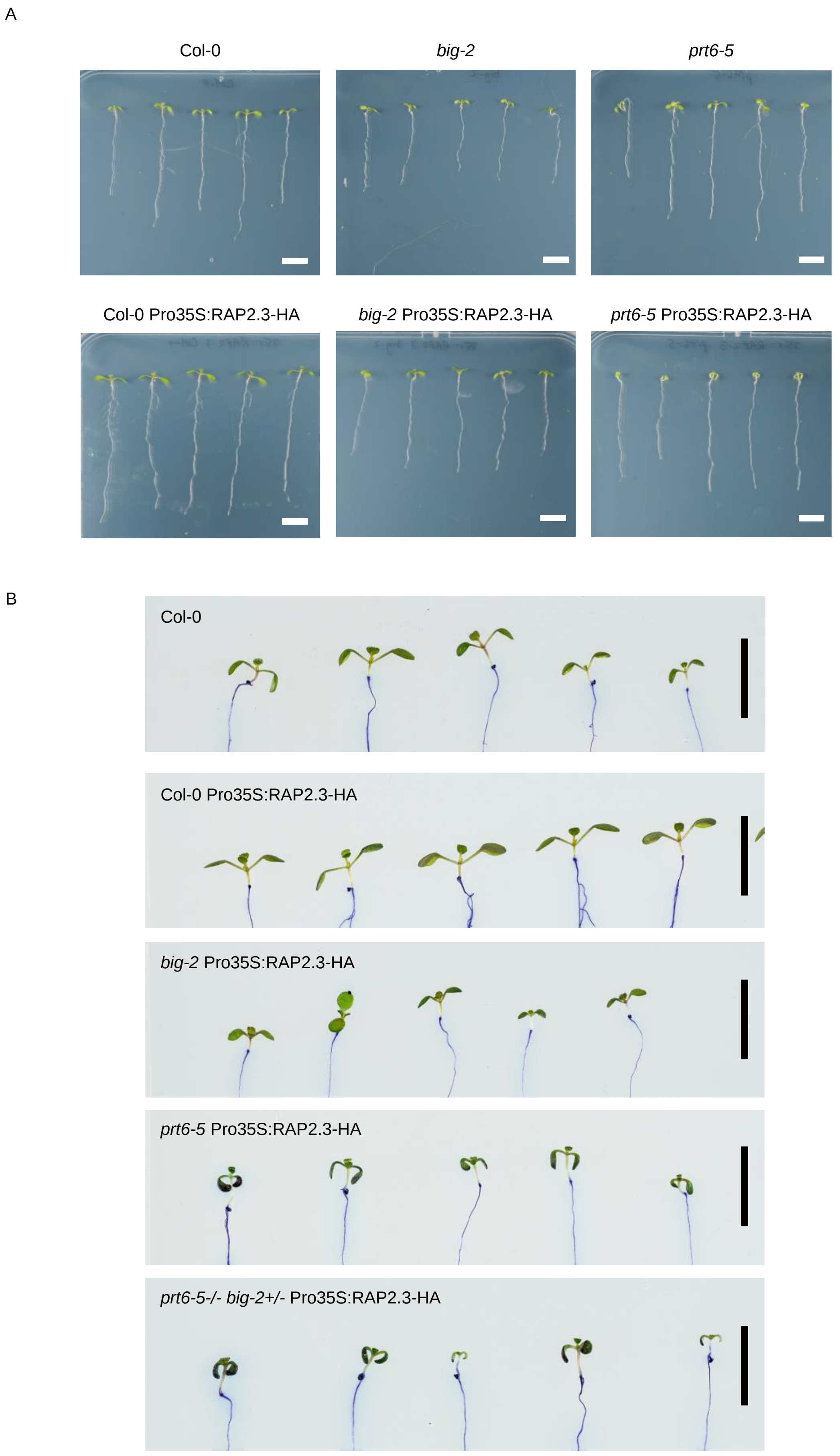
A
Col-0
big-2
prt6-5
Col-0 Pro35S:RAP2.3-HA
big-2 Pro35S:RAP2.3-HA
prt6-5 Pro35S:RAP2.3-HA
B
Col-0
Col-0 Pro35S:RAP2.3-HA
big-2 Pro35S:RAP2.3-HA
prt6-5 Pro35S:RAP2.3-HA
prt6-5-/- big-2+/- Pro35S:RAP2.3-HA

Supplementary Figure S3. Morphology of seedlings ectopically expressing RAP2.3-HA. Supports Figure 3

(A) 8 d-old seedlings grown on 0.5 X MS containing 1 % (w/v) sucrose under long days. Bar = 1 cm.
(B) Toluidine Blue staining of 8 d-old seedlings grown on 0.5 X MS containing 1% (w/v) sucrose under long days. After staining, seedlings were rearranged on a fresh plate for photography. Bar = 1 cm. Representative of two independent experiments.

A

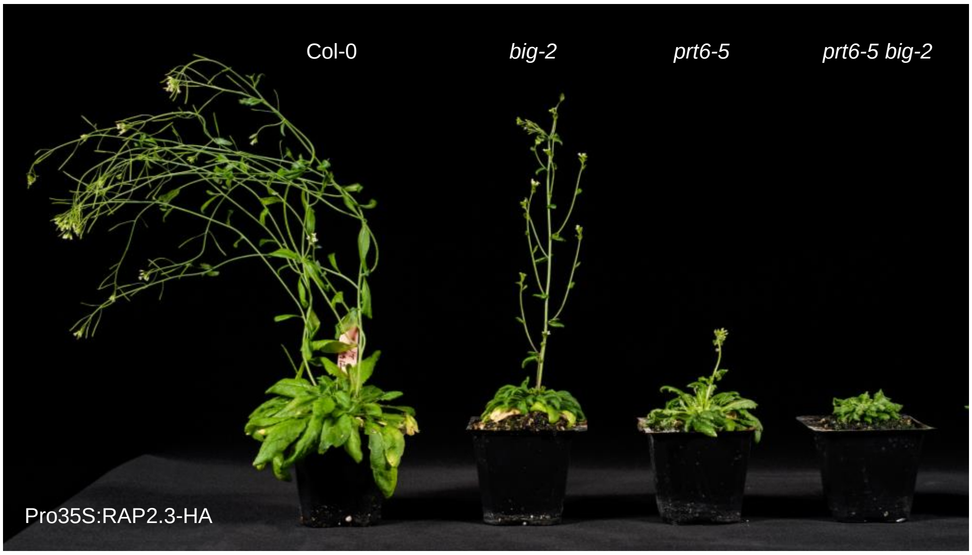

B

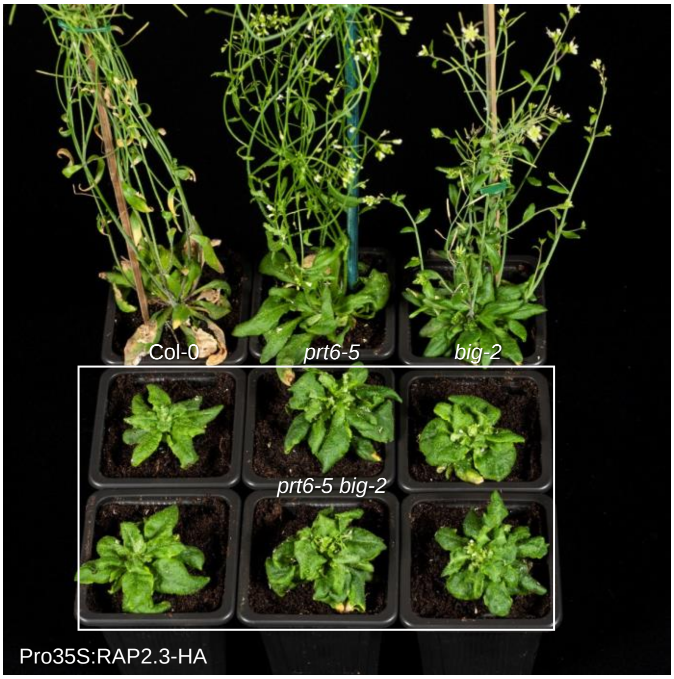

C

Pro35S:RAP2.3-HA

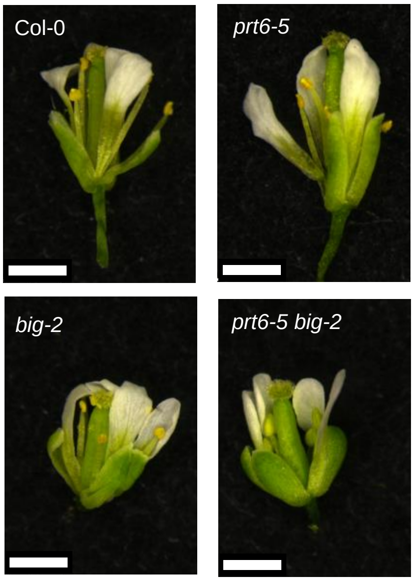

Supplementary Figure S4. Morphology of plants ectopically expressing RAP2.3-HA. Supports Figure 3

(A) Plants grown in soil for 12 weeks in neutral days. (B) Plants germinated on 0.5 X MS containing 0.5 % (w/v) sucrose, transplanted to soil, and photographed after 5 weeks under long day conditions. The white box contains six *prt6-5 big-2* plants expressing Pro35S:RAP2.3-HA. (C) Flowers from the primary bolt of different genotypes expressing Pro35S:RAP2.3-HA were partially dissected to reveal stamens and gynoecia. Bar = 1 mm.

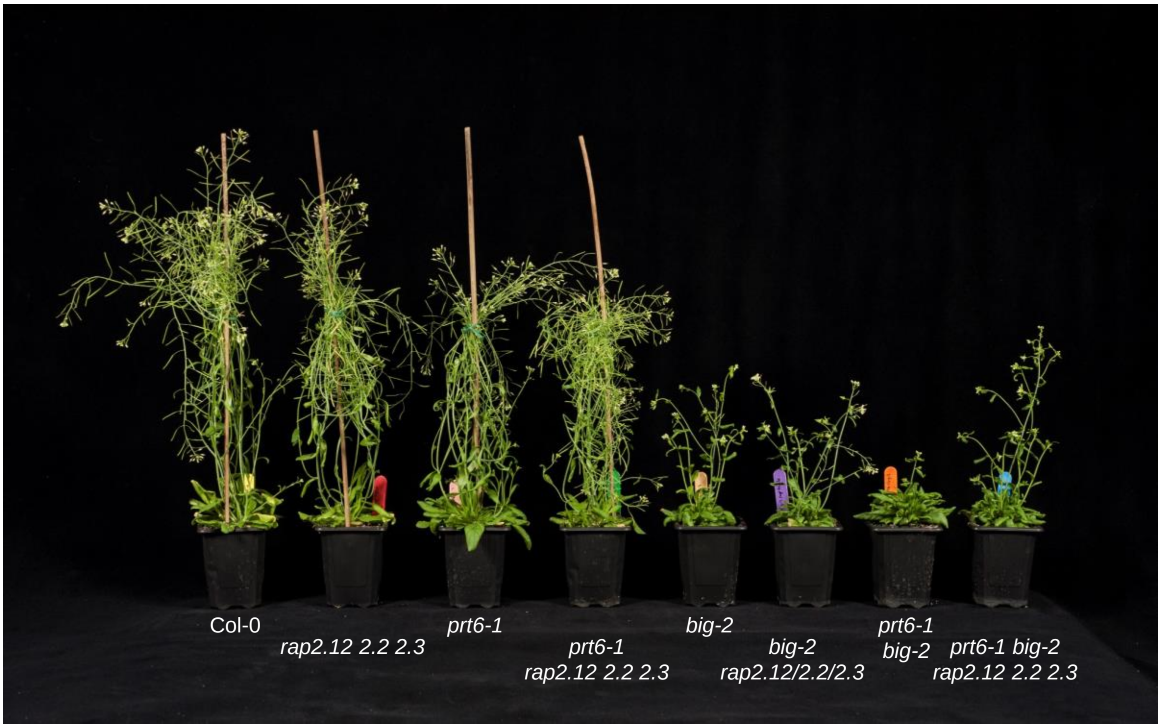

Supplementary Figure S5. Morphology of PRT6/N-degron pathway and *erfvii* combination mutants. Supports Figure 4.

Plants were grown in soil for six weeks under long day conditions, prior to photography.

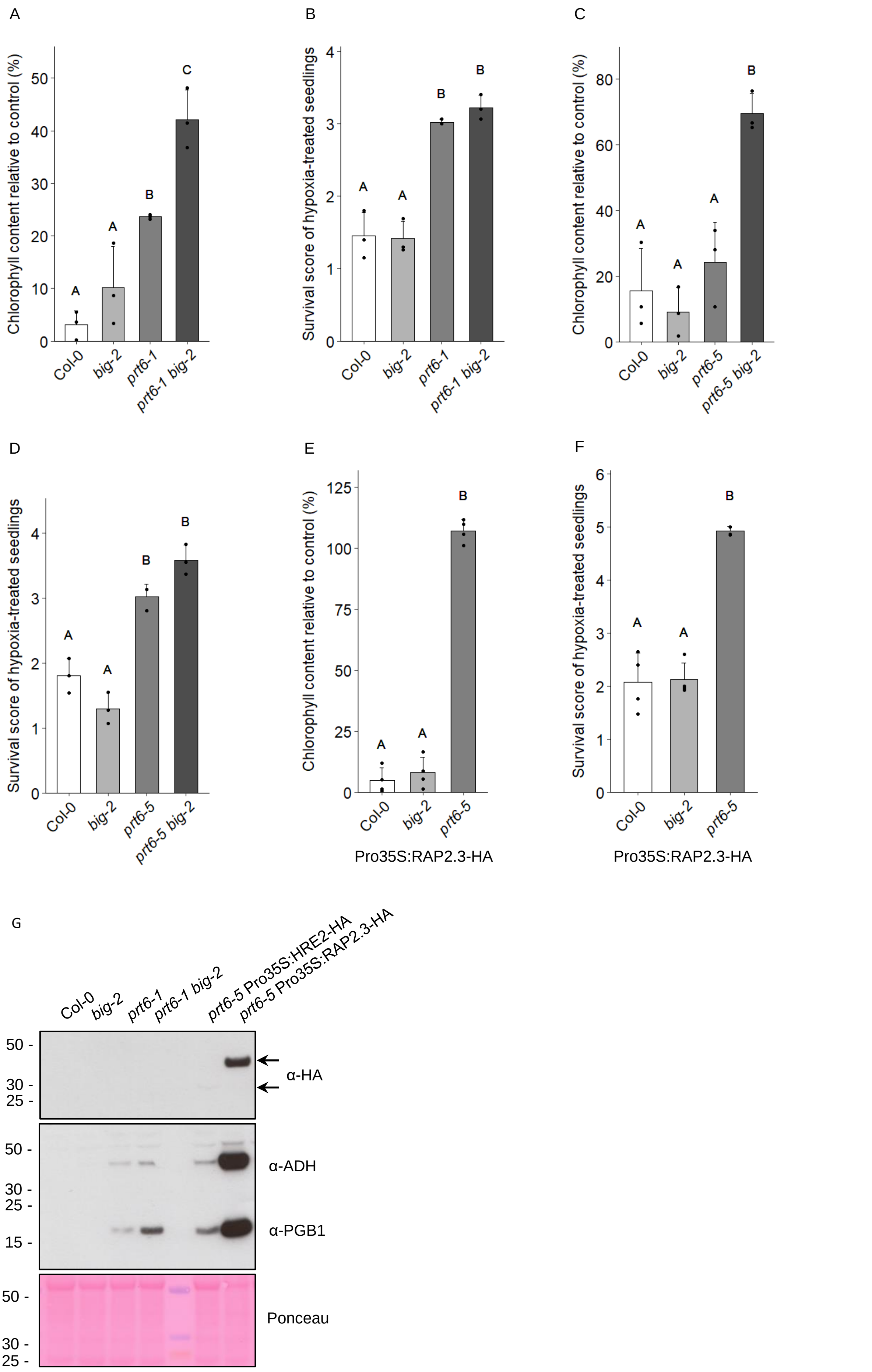
A
Chlorophyll content relative to control (%)
Col-0
big-2
prt6-1
prt6-1 big-2
B
Survival score of hypoxia-treated seedlings
Col-0
big-2
prt6-1
prt6-1 big-2
C
Chlorophyll content relative to control (%)
Col-0
big-2
prt6-5
prt6-5 big-2
D
Survival score of hypoxia-treated seedlings
Col-0
big-2
prt6-5
prt6-5 big-2
E
Chlorophyll content relative to control (%)
Col-0
big-2
prt6-5
Pro35S:RAP2.3-HA
F
Survival score of hypoxia-treated seedlings
Col-0
big-2
prt6-5
Pro35S:RAP2.3-HA
G
Col-0
big-2
prt6-1
prt6-1 big-2
prt6-5 Pro35S:HRE2-HA
prt6-5 Pro35S:RAP2.3-HA
α-HA
α-ADH
α-PGB1
Ponceau

Supplementary Figure S6. Hypoxia response of PRT6/N-degron pathway mutants. Supports Figures 3 and 4.

Seedlings of untransformed mutants (A-D) and transgenic lines ectopically expressing Pro35S:RAP2.3-HA (E,F) were grown on 0.5 X MS medium containing 0.5 % (w/v) sucrose for 4 d under long days, subjected to 5 h hypoxia, and analysed after 3 d recovery in the light. (A,C,E) Chlorophyll content of hypoxia treated seedlings relative to control (A,C: n=3 replicates of 20-30 seedlings each; E: n=4); (B,D,F) Survival data. Values are means ± SD (B,D: n=3 replicates of 20-30 seedlings each; F: n=4). For all plots, different letters indicate significant differences between conditions ($P < 0.05$; ANOVA with Tukey multiple comparison test). Representative of several independent experiments. (G) Immunoblots of crude protein extracts from 6-d old seedlings of the indicated genotypes, probed with antibodies specific for the hypoxia markers, ALCOHOL DEHYDROGENASE (ADH) and PHYTOGLOBIN1 (PGB1) (which were applied to the same membrane) and for the HA tag. Ponceau S staining was used to confirm equal loading. Positions of HA-tagged ERFVIIs are indicated by arrows; relative molecular mass markers (kDa) are shown to the left of the panel.

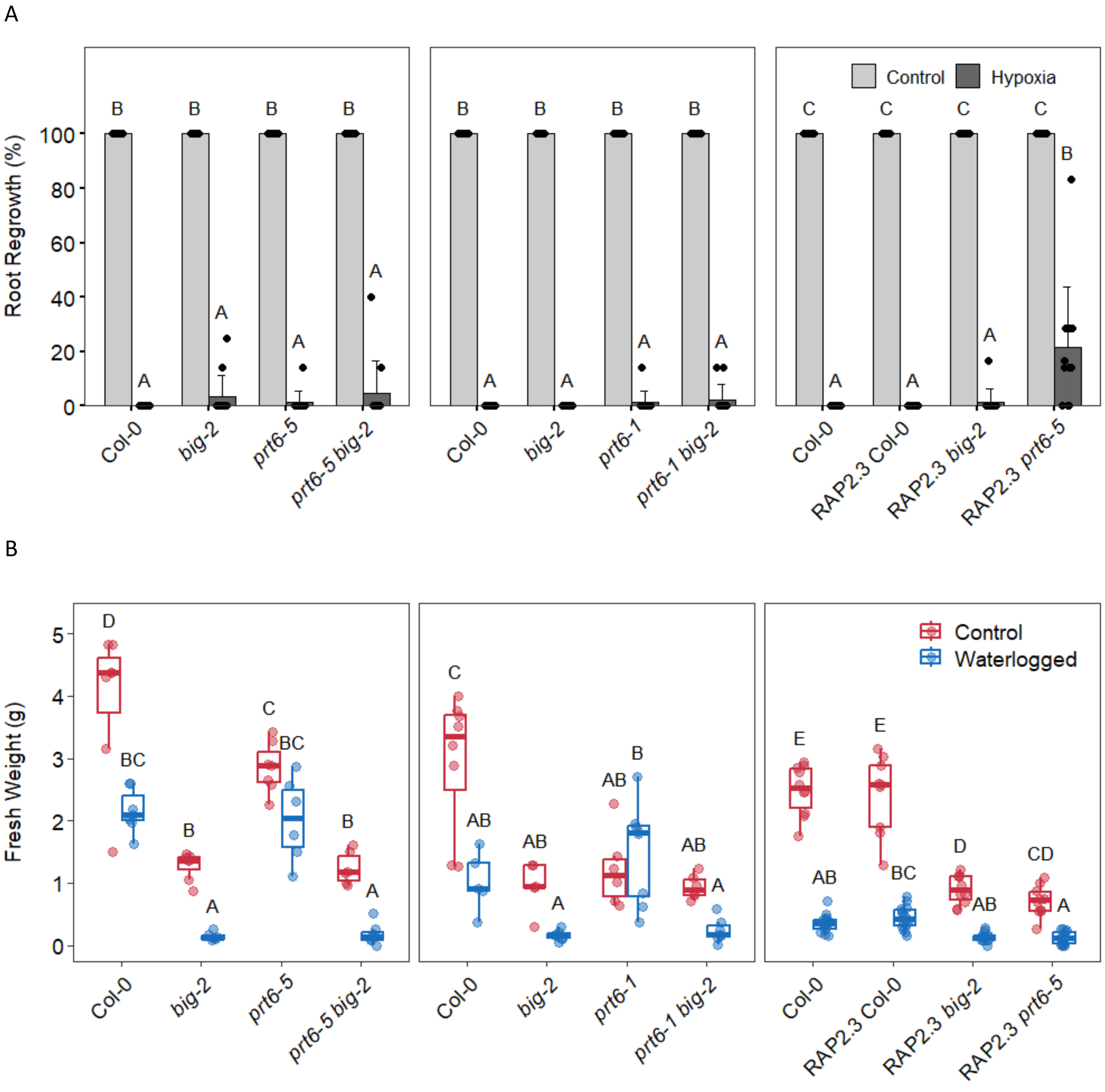

Supplementary Figure S7. Response of PRT6/N-degron pathway mutants to hypoxia and waterlogging. Supports Figures 3 and 4.
(A) Regrowth of roots following hypoxia treatment of untransformed mutants and transgenic lines expressing Pro35S:RAP2.3-HA (RAP2.3). 5 d old seedlings were grown on 0.5 x MS plates containing 0.5 % (w/v) sucrose and subjected to 4 h hypoxia in the dark. Root regrowth was scored after 2 d in the light. Values are means ± SD, with individual data points overlaid (n=12 replicates of 7 seedlings each). (B) Rosette fresh weight of untransformed mutants and transgenic lines expressing Pro35S:RAP2.3-HA (RAP2.3) following waterlogging. 12 d-old seedlings were transferred to soil and grown under neutral days (12 h L:12 h D) for 14 d then subjected to waterlogging for 21 days or watered as normal with good drainage. The boxes indicate the median and interquartile range with individual points overlaid (5-15 plants/treatment). The separate panels represent individual experiments and letters indicate differences in statistical significance ($p<0.05$; ANOVA with Tukey multiple comparison test).

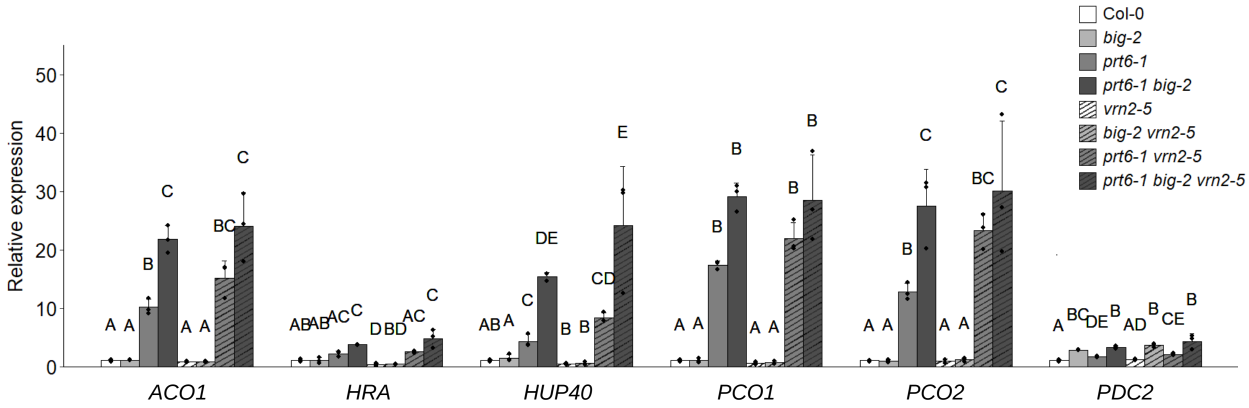

Supplementary Figure S8. Relative expression of hypoxia responsive genes in N-degron pathway mutants Supports Figure 4.

Expression of selected “core 49” hypoxia response genes in 6-d old seedlings of the indicated genotypes relative to Col-0. Values are means ± SD (n=3). Different letters indicate significant differences between conditions (*P* < 0.05; ANOVA with Tukey multiple comparison test).

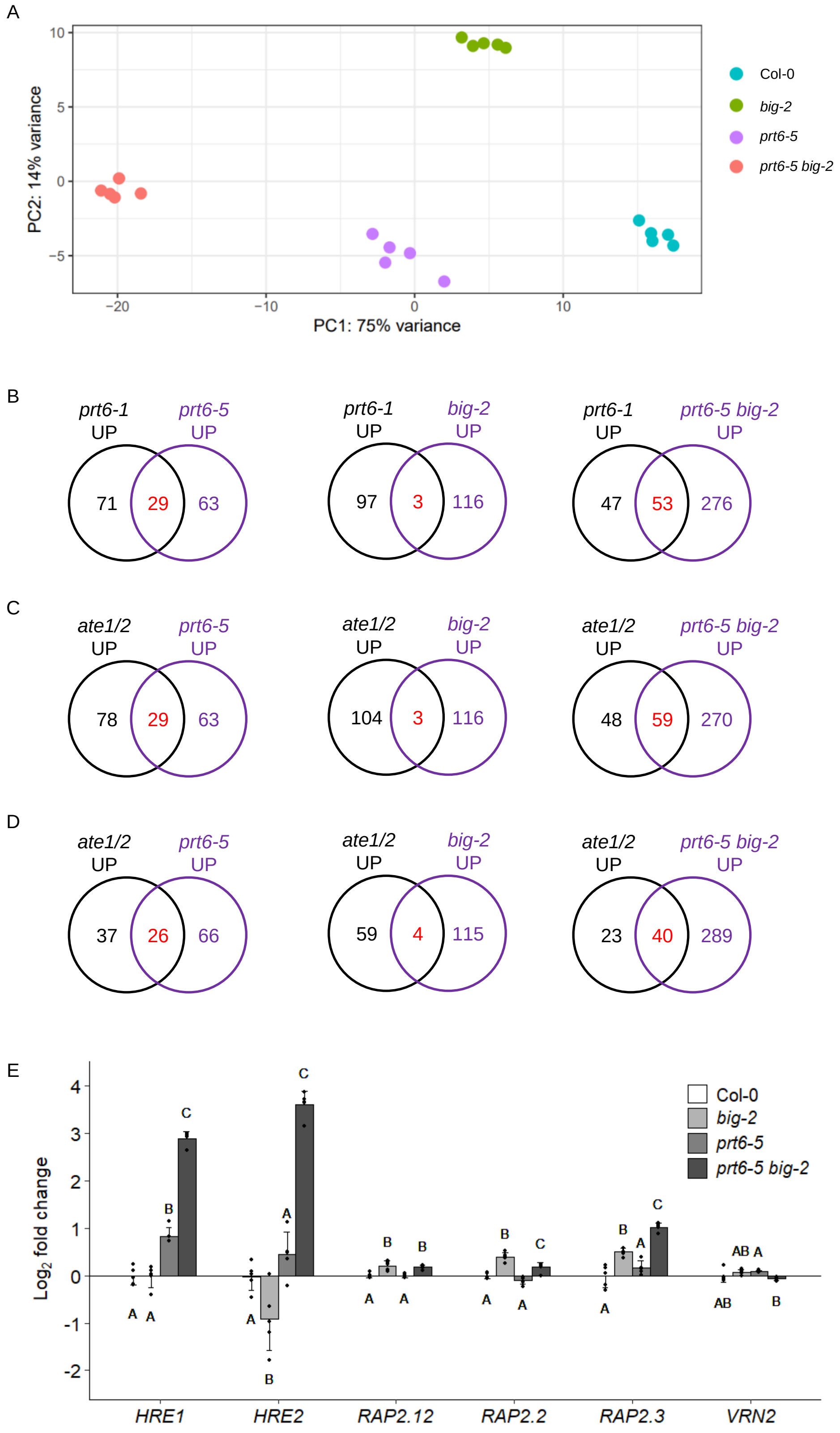
A
PC2: 14% variance
PC1: 75% variance
Col-0
big-2
prt6-5
prt6-5 big-2
B
prt6-1 UP
prt6-5 UP
71
29
63
prt6-1 UP
big-2 UP
97
3
116
prt6-1 UP
prt6-5 big-2 UP
47
53
276
C
ate1/2 UP
prt6-5 UP
78
29
63
ate1/2 UP
big-2 UP
104
3
116
ate1/2 UP
prt6-5 big-2 UP
48
59
270
D
ate1/2 UP
prt6-5 UP
37
26
66
ate1/2 UP
big-2 UP
59
4
115
ate1/2 UP
prt6-5 big-2 UP
23
40
289
E
Log2 fold change
Col-0
big-2
prt6-5
prt6-5 big-2
HRE1
HRE2
RAP2.12
RAP2.2
RAP2.3
VRN2

Supplementary Figure S9. Transcriptome analysis of N-degron pathway mutants. Supports Figure 5.

(A) Principal component analysis of RNA-seq data. (B-D) Venn diagrams showing comparison of differentially expressed genes from the RNA-seq data reported in this study (purple) with differentially regulated genes in published microarray studies (black): (B) *prt6-1* seedlings (Gibbs et al., 2011), (C) *ate1 ate2* seedlings (Gibbs et al., 2011), (D) *ate1 ate2* seedlings (de Marchi et al., 2016). Common differentially expressed genes are indicated in red. (E) Expression of genes encoding known PRT6/N-degron pathway substrates in roots of different mutant backgrounds, relative to Col-0. Data were re-plotted from the RNA-seq data set; values are means ± SD (n=5) ; different letters indicate significant differences between conditions (P < 0.05; ANOVA with Tukey multiple comparison test). *LITTLE ZIPPER2* (*ZPR2*) was not identified in the data set.

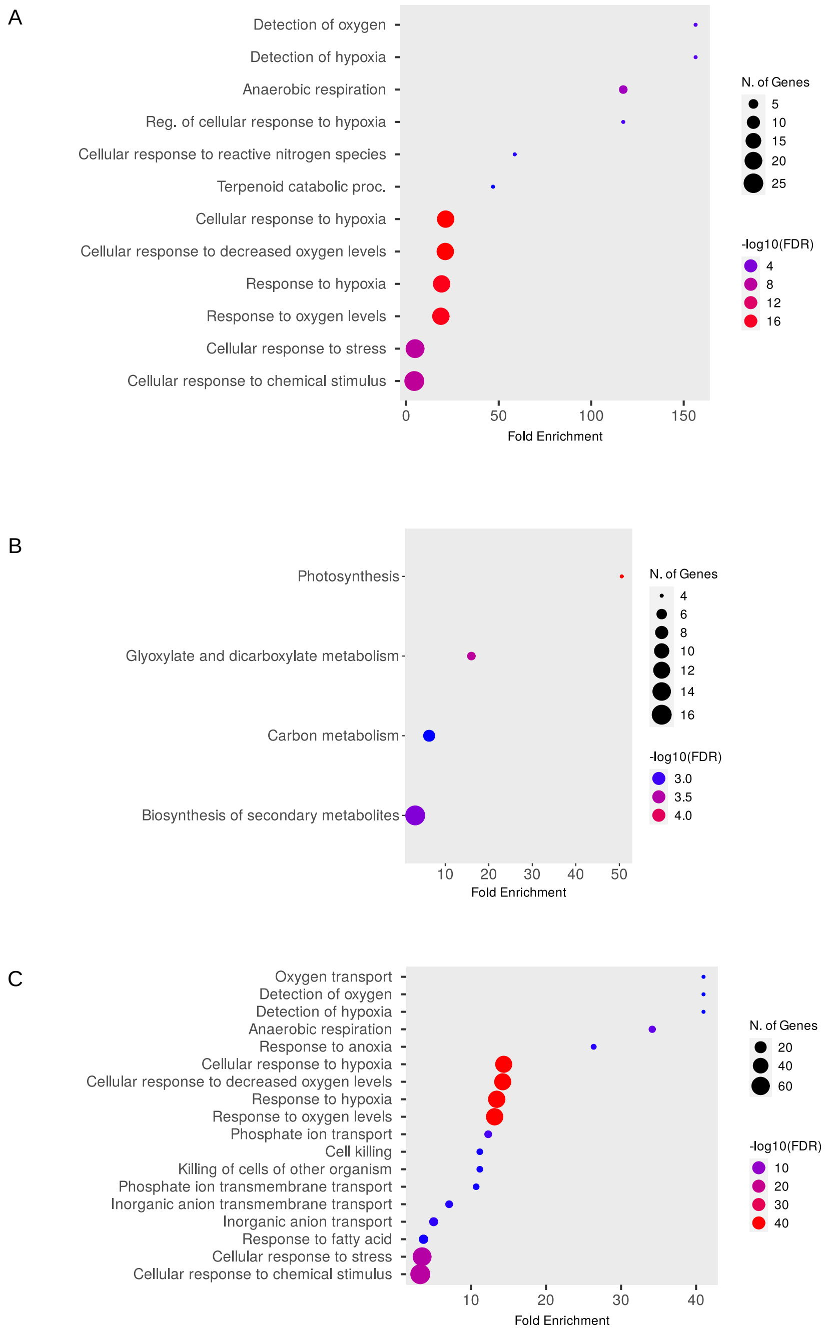

Supplementary Figure S10. Gene Ontology term enrichment in genes up-regulated in roots of N-degron pathway mutants. Supports Figure 5.

Differentially expressed gene lists were analysed with ShinyGO (Ge et al., 2020) using all genes identified in the RNA-seq data set as background. (A) *prt6-5*. (B) *big-2*. (C) *prt6-5 big-2*.

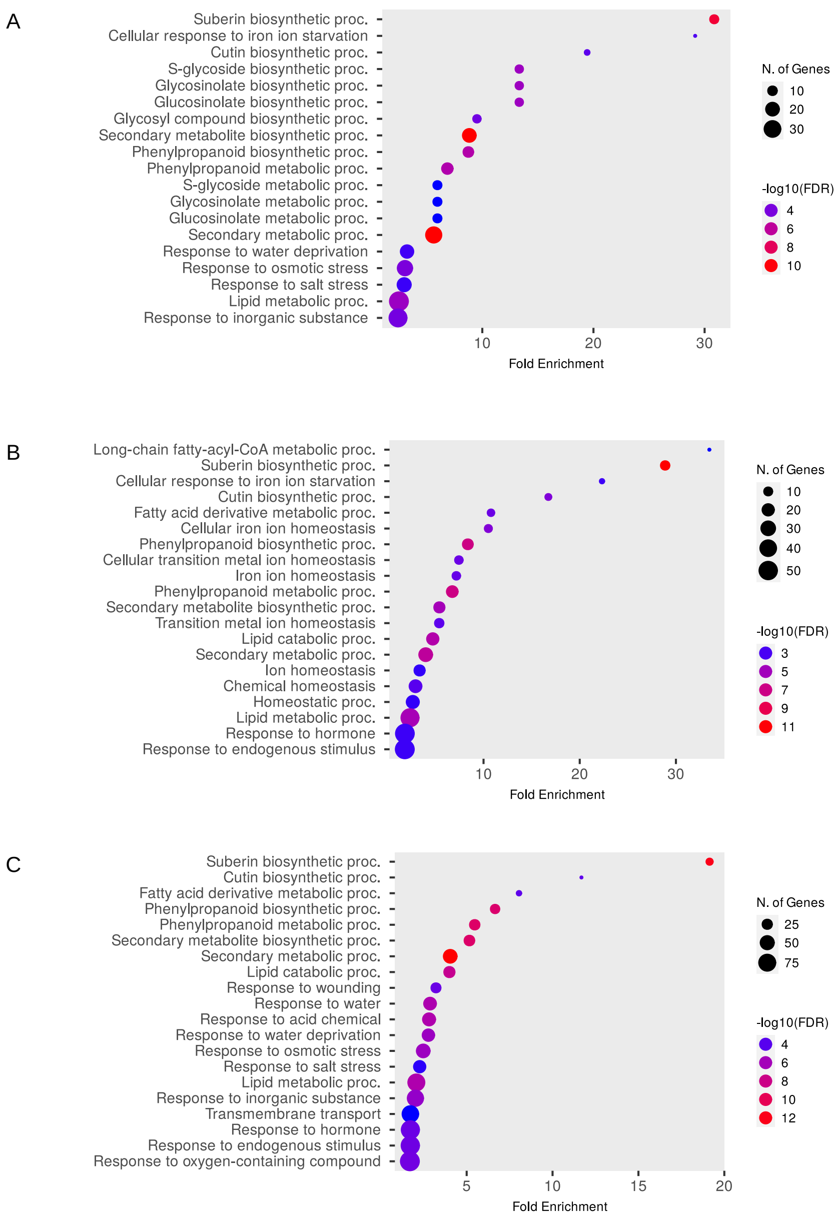

Supplementary Figure S11. Gene Ontology term enrichment in genes down-regulated in roots of N-degron pathway mutants. Supports Figure 5.

Differentially expressed gene lists were analysed with ShinyGO (Ge et al., 2020) using all genes identified in the RNA-seq data set as background. (A) *prt6-5*. (B) *big-2*. (C) *prt6-5 big-2*.

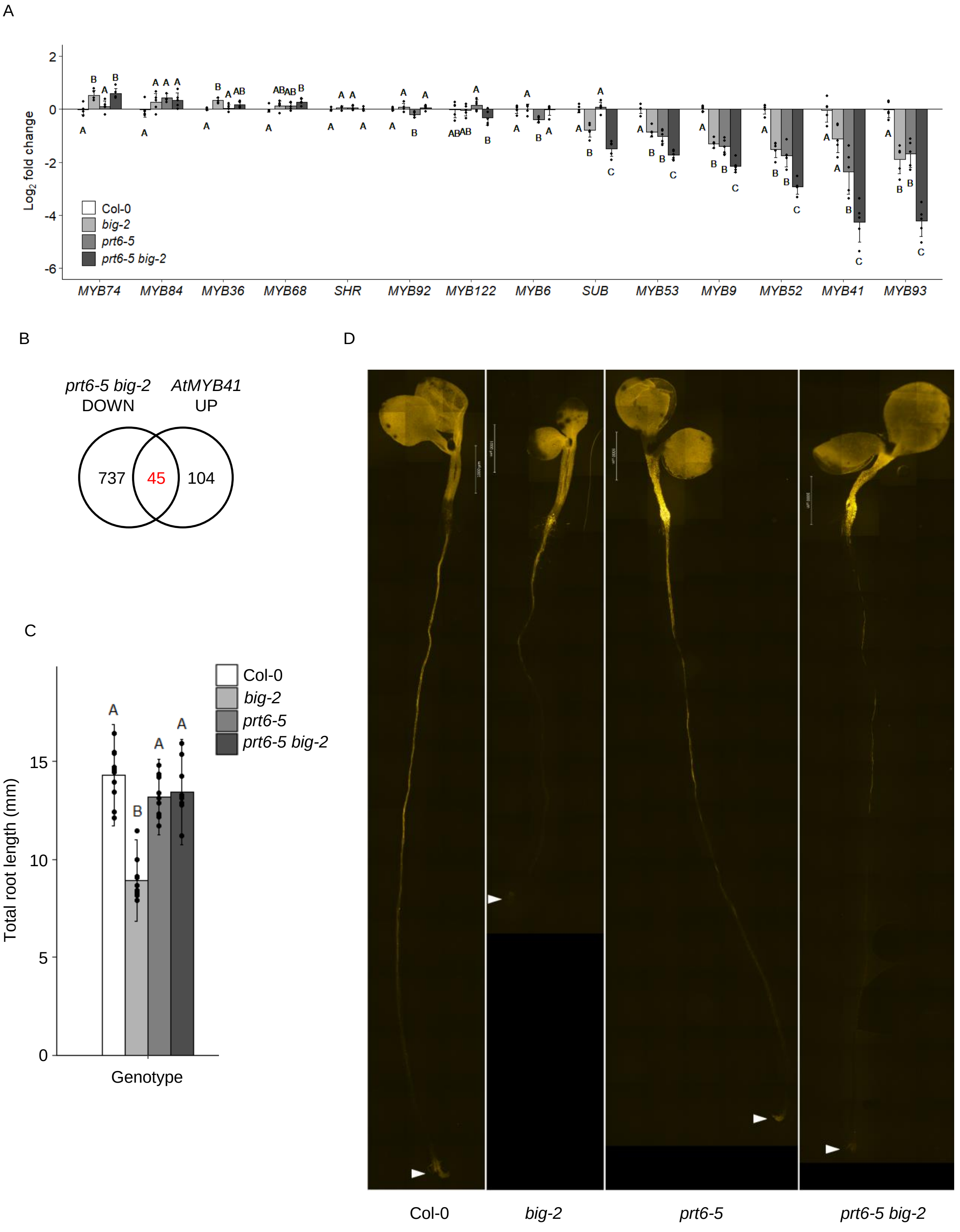
A
Log2 fold change
2
0
-2
-4
-6
Col-0
big-2
prt6-5
prt6-5 big-2
MYB74
MYB84
MYB36
MYB68
SHR
MYB92
MYB122
MYB6
SUB
MYB53
MYB9
MYB52
MYB41
MYB93
B
prt6-5 big-2
DOWN
AtMYB41
UP
737
45
104
C
Total root length (mm)
15
10
5
0
Genotype
Col-0
big-2
prt6-5
prt6-5 big-2
D
Col-0
big-2
prt6-5
prt6-5 big-2

Supplementary Figure S12. Suberin biosynthesis and deposition in N-degron pathway mutants. Supports Figure 6.

(A) Expression of transcription factors involved in suberin biosynthesis (Shukla et al., 2021; Xu et al., 2022) relative to Col-0. Data were re-plotted from the RNA-seq data set; values are means ± SD (n=5) different letters indicate significant differences between genotypes ($P < 0.05$; ANOVA with Tukey multiple comparison test). (B) Venn diagram showing comparison of genes down-regulated in *prt6-5 big-2* roots with genes up-regulated by ectopic expression of *MYB41* from (Cominelli et al., 2008); common differentially expressed genes are indicated in red. (C) Root length of seedlings used for suberin staining; data represent means ± SD (n=10); different letters indicate significant differences between genotypes ($P < 0.05$). (D) Representative composite micrographs showing Fluorol Yellow 088 staining of suberin in wild type and mutant roots (scale bars represent 1 mm). White arrowheads indicate positions of the root tips. C and D are representative of two independent experiments.

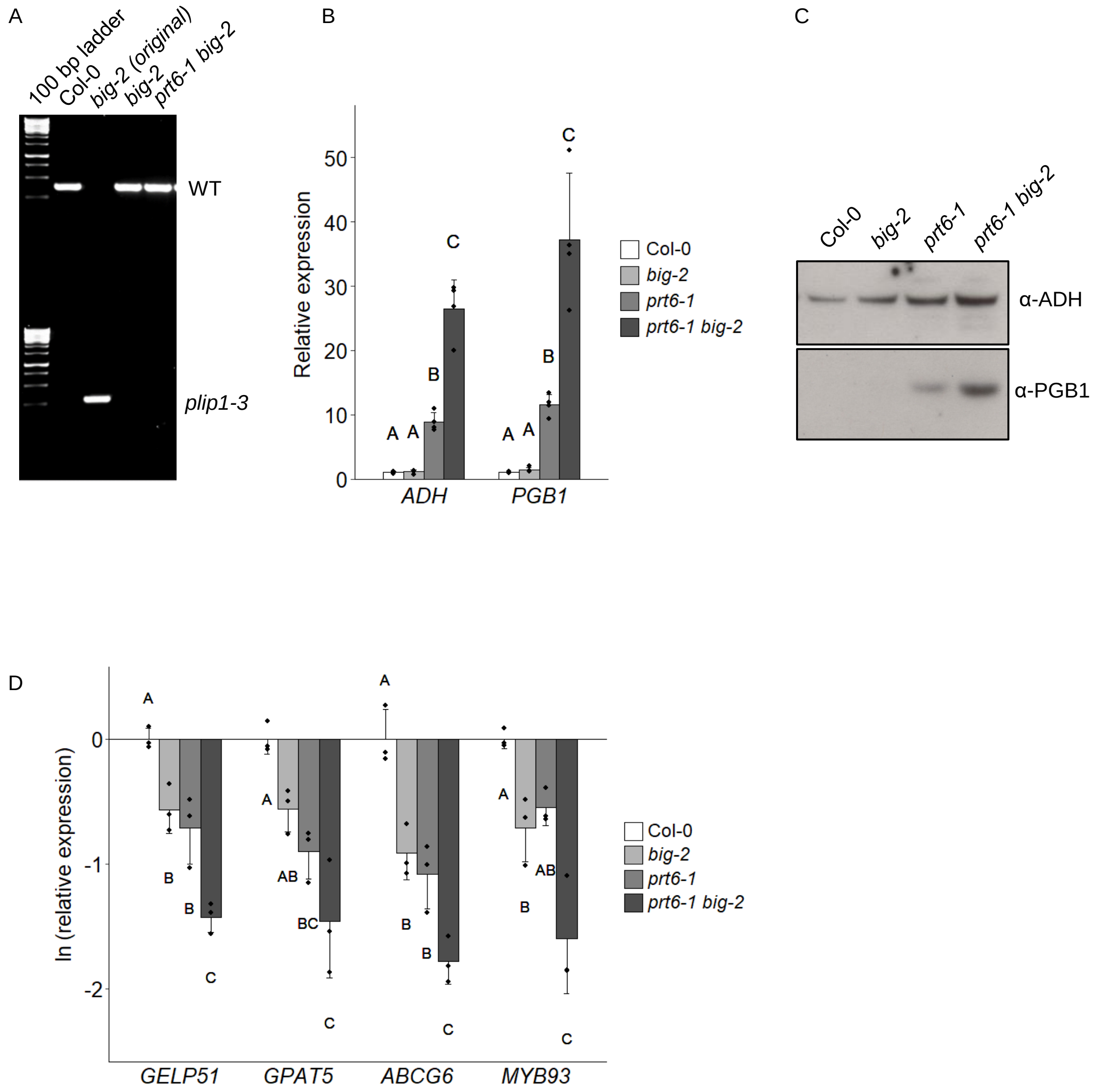

Supplementary Figure S13. Relative expression of hypoxia and suberin genes in *big-2* combination mutants lacking the *plip1-3* T-DNA.
Supports Figures 5 and 6.

(A) Genotyping PCR for *plip1-3* T-DNA, showing original *big-2* stock and mutants in which the second T-DNA has been segregated out. (B) RT-qPCR analysis of hypoxia responsive genes in 5-d old seedlings of mutants relative to Col-0. Values are means ± SD (n=4); different letters indicate significant differences between genotypes ($P < 0.05$). (C) Immunoblot of crude protein extracts from 6-d old seedlings of the indicated genotypes, probed with anti-HA (α-HA) antiserum or antibodies specific for the hypoxia markers, ALCOHOL DEHYDROGENASE (ADH) and PHYTOGLOBIN1 (PGB1). (D) RT-qPCR analysis of genes involved in suberin biosynthesis and deposition in 5-day old roots of mutants relative to Col-0. Values are means ± SD (n=3); different letters indicate significant differences between genotypes ($P < 0.05$; ANOVA with Tukey multiple comparison test).

M/RRSGGGGGGGGGGGGGRGGSGAWLLPVSLVKRKTTLAPNTQTASPRAL
ADSVNGKPIPNPLLGLDSTASKDNTVPLKLIALLANGEFHSGEQLGETLGMS
RAAINKHIQTLRDWGVDVFTVPGKGYSLPEPIPLLNAKQILGQLDGGSVAVLP
VVDSTNQYLLDRIGELKSGDACIAEYQQAGRGSRGRKWFSPFGANLYLSMF
WRLKRGPAAIGLGPVIGIVMAEALRKLGADKVRVKWPNDLYLQDRKLAGILV
ELAGITGDAAQIVIGAGINVAMRRVEESVVNQGWITLQEAGINLDRNTLAATLI
RELRAALELFEQEGLAPYLPRWEKLDNFINRPVKLIIGDKEIFGISRGIDKQGA
LLLEQDGVIKPWMGGEISLRSAEKLQLPPLERLTLDGGGGSGGGSMVSKGE
ELFTGVVPILVELDGDVNGHKFSVSGEGEGDATYGKLTLKFICTTGKLPVPW
PTLVTTFGYGLQCFARYPDHMKQHDFFKSAMPEGYVQERTIFFKDDGNYKT
RAEVKFEGDTLVNRIELKGIDFKEDGNILGHKLEYNYNSHNVYIMADKQKNGI
KVNFKIRHNIEDGSVQLADHYQQNTPIGDGPVLLPDNHYLSYQSALSKDPNE
KRDHMVLLEFVTAAGITLGMDELYK*

Supplementary Figure S14 Amino acid sequence of R-Turbo and M-Turbo.

Single letter amino acid code is used. The N-terminal residue revealed after cleavage in planta is indicated in red.

Supplementary Table S1. Genetic materials used in this study.

Line	Description	Source/reference
Col-0	Wild type accession	NASC
prt6-1	T-DNA insertion mutant; SAIL_1278_H11	Holman et al., 2009
prt6-5	T-DNA; SALK_0510_88	Graciet et al., 2009
big-2	T-DNA; SALK_045560	Kasajima et al., 2007; Ivanova et al., 2014
prt6-1 big-2		This study
prt6-5 big-2		This study
prt1-1	EMS allele; Q111-STOP	Garzón et al., 2007; NASC accession, N119
ate1-2 ate 2-1 (ate1 ate2)	T-DNA; SALK_023492 T-DNA; SALK_040788	Holman et al., 2009; Graciet et al., 2009
ate1 ate2 big-2		This study
rap2.12 rap2.2 rap2.3	*rap2.12-1*, GABI-KAT line GK_503A1_11 *rap2.2-1*, SAIL184_G12 *rap2.3-1* allele; Ogawa et al., 2007	Gibbs et al., 2014; Zhang et al., 2018a
prt6-1 rap2.12 rap2.2 rap2.3		Gibbs et al., 2014
big-2 rap2.12 rap2.2 rap2.3		This study
prt6-1 big-2 rap2.12 rap2.2 rap2.3		This study
vrn2-5	SALK_201153	Gibbs et al., 2018
prt6-1 vrn2-5		Gibbs et al., 2018
big-2 vrn2-5		This study
prt6-1 big-2 vrn2-5		This study
R-GUS Col-0		Garzón et al., 2007
R-GUS *prt6-1*		Garzón et al., 2007
R-GUS *big-2*		This study
R-GUS *prt6-1 big-2*		This study
F-GUS Col-0		Garzón et al., 2007
F-GUS *prt1-1*		This study
F-GUS *big-2*		This study
F-GUS *prt1-1 big-2*		This study

M-GUS Col-0		Garzón et al., 2007
M-GUS *prt1-1*		Garzón et al., 2007
M-GUS *prt6-1*		This study
M-GUS *big-2*		This study
M-GUS *prt6-1 big-2*		This study
R-LUC Col-0		Graciet et al., 2010
R-LUC *prt6-5*		This study
R-LUC *big-2*		This study
R-LUC *prt6-5 big-2*		This study
Pro35S:RAP2.3-HA Col-0		Gibbs et al., 2014
Pro35S:RAP2.3-HA Col-0 *prt6-5*		This study
Pro35S:RAP2.3-HA Col-0 *big-2*		This study
Pro35S:RAP2.3-HA Col-0 *prt6-5 big-2*		This study
Pro35S:HRE2-HA Col-0		Gibbs et al., 2011
Pro35S:HRE2-HA Col-0 *prt6-5*		This study
Pro35S:HRE2-HA Col-0 *big-2*		This study
Pro35S:HRE2-HA Col-0 *prt6-5 big-2*		This study
ProVRN2:VRN2-GUS Col-0		This study
ProVRN2:VRN2-GUS *prt6-1*		Gibbs et al., 2018
ProVRN2:VRN2-GUS *big-2*		This study
ProVRN2:VRN2-GUS *prt6-1 big-2*		This study
ProUBQ10:M-Turbo-NES-YFP (M-Turbo) Col-0		This study
ProUBQ10:R-Turbo-NES-YFP (R-Turbo) Col-0		This study

References

Barnsby-Greer L, Mabbitt PD, Dery MA, Squair DR, Wood NT, Lamoliatte F, Lange SM, Virdee S. (2024) UBE2A and UBE2B are recruited by an atypical E3 ligase module in UBR4. Nat Struct Mol Biol. doi: 10.1038/s41594-023-01192-4.

Cominelli E, Sala T, Calvi D, Gusmaroli G, Tonelli C. (2008) Over-expression of the Arabidopsis *AtMYB41* gene alters cell expansion and leaf surface permeability. Plant J. 53(1): 53-64

de Marchi R, Sorel M, Mooney B, Fudal I, Goslin K, Kwaśniewska K, Ryan PT, Pfalz M, Kroymann J, Pollmann S, et al. (2016) The N-end rule pathway regulates pathogen responses in plants. Sci. Rep. 6: 26020

Garzón M, Eifler K, Faust A, Scheel H, Hofmann K, Koncz C, Yephremov A, Bachmair, A. (2007) PRT6/At5g02310 encodes an Arabidopsis ubiquitin ligase of the N-end rule pathway with arginine specificity and is not the *CER3* locus. FEBS Lett. 581: 3189-3196

Ge SX, Jung D, Yao R. (2020) ShinyGO: a graphical gene-set enrichment tool for animals and plants. Bioinformatics 36(8): 2628-2629

Gibbs DJ, Isa NM, Movahedi M, Lozano-Juste J, Mendiondo GM, Berckhan S, Marín-de la Rosa N, Vicente Conde J, Sousa Correia C, Pearce SP, et al. (2014) Nitric oxide sensing in plants is mediated by proteolytic control of group VII ERF transcription factors. Mol. Cell. 53: 369-379

Gibbs DJ, Lee SC, Isa NM, Gramuglia S, Fukao T, Bassel GW, Correia CS, Corbineau F, Theodoulou FL, Bailey-Serres J, et al. (2011) Homeostatic response to hypoxia is regulated by the N-end rule pathway in plants. Nature 479: 415-418

Gibbs DJ, Tedds HM, Labandera AM, Bailey M, White MD, Hartman S, Sprigg C, Mogg SL, Osborne R, Dambire C, et al. (2018) Oxygen-dependent proteolysis regulates the stability of angiosperm polycomb repressive complex 2 subunit VERNALIZATION 2. Nat. Commun. 9: 5438

Gil P, Dewey E, Friml J, Zhao Y, Snowden KC, Putterill J, Palme K, Estelle M, Chory J. (2001) BIG: a calossin-like protein required for polar auxin transport in Arabidopsis. Genes Dev. 15: 1985-1997

Graciet E, Mesiti F, Wellmer F. (2010) Structure and evolutionary conservation of the plant N-end rule pathway. Plant J. 61: 741-751

Graciet E, Walter F, Ó'Maoiléidigh DS, Pollmann S, Meyerowitz EM, Varshavsky A, Wellmer F. (2009) The N-end rule pathway controls multiple functions during Arabidopsis shoot and leaf development. Proc. Natl. Acad. Sci. U. S. A. 106: 13618-13623

He J, Zhang RX, Peng K, Tagliavia C, Li S, Xue S, Liu A, Hu H, Zhang J, Hubbard KE, et al. (2018) The BIG protein distinguishes the process of CO_2-induced stomatal closure from the inhibition of stomatal opening by CO_2. New Phytol. 218: 232-241

Holman TJ, Jones PD, Russell L, Medhurst A, Ubeda Tomás S, Talloji P, Marquez J, Schmuths H, Tung SA, Taylor I, et al. (2009) The N-end rule pathway promotes seed germination and establishment through removal of ABA sensitivity in Arabidopsis. Proc. Natl. Acad. Sci. U. S. A. 106: 4549-4554

Ivanova A, Law SR, Narsai R, Duncan O, Lee JH, Zhang B, Van Aken O, Radomiljac JD, van der Merwe M, Yi, J et al. (2014) A Functional Antagonistic Relationship between Auxin and Mitochondrial Retrograde Signaling Regulates Alternative Oxidase1a Expression in Arabidopsis. Plant Physiol. 165: 1233-1254

Kasajima I, Ohkama-Ohtsu N, Ide Y, Haysashi H, Yoneyama T, Suzuki Y, Naito S, Fujiwara T. (2007) The *BIG* gene is involved in regulation of sulfur deficiency-responsive genes in *Arabidopsis thaliana*. Physiol. Plant. 129: 351-363

Mair A, Xu SL, Branon TC, Ting AY, Bergmann DC, (2019) Proximity labeling of protein complexes and cell-type-specific organellar proteomes in Arabidopsis enabled by TurboID. Elife 8: e47864

Ogawa T, Uchimiya H, Kawai-Yamada M. (2007) Mutual regulation of *Arabidopsis thaliana* ethylene-responsive element binding protein and a plant floral homeotic gene, *APETALA2*. Ann. Bot. (Lond.) 99: 239–244

Shukla V, Han JP, Cléard F, Lefebvre-Legendre L, Gully K, Flis P, Berhin A, Andersen TG, Salt DE, Nawrath C, Barberon M (2021) Suberin plasticity to developmental and exogenous cues is regulated by a set of MYB transcription factors. Proc Natl Acad Sci U S A. 118: e2101730118

Worley CK, Ling R, Callis J, (1998) Engineering in vivo instability of firefly luciferase and Escherichia coli beta-glucuronidase in higher plants using recognition elements from the ubiquitin pathway. Plant Mol. Biol. 37: 337–347

Xu H, Liu P, Wang C, WS, Dong C, Lin Q, Sun W, Huang B, Xu M, Tauqeer A, Wu S. (2022) Transcriptional networks regulating suberin and lignin in endodermis link development and ABA response. Plant Physiol. 190(2): 1165-1181

Zhang H, Gannon L, Hassall KL, Deery MJ, Gibbs DJ, Holdsworth MJ, van der Hoorn RAL, Lilley KS, Theodoulou FL. (2018) N-terminomics reveals control of Arabidopsis seed storage proteins and proteases by the Arg/N-end rule pathway. New Phytol. 218: 1106-1126
