# Supplementary material for: BIG enhances Arg/N-degron pathway-mediated protein degradation to regulate Arabidopsis hypoxia responses and suberin deposition
Source: Plant Cell. 2024 Apr 12;36(9):3177–200. doi: 10.1093/plcell/koae117 (PMC11371152; doi:10.1093/plcell/koae117)
Supplement: koae117_Supplementary_Data [file tpc.00575.2023-s08.pdf › tpc.00575.2023-s08.pdf]

THE PLANT CELL

BIG enhances protein degradation through the Arg/N-degron pathway to regulate the hypoxia response and suberin deposition in Arabidopsis.

Hongtao Zhang, Chelsea Rundle, Nikola Winter, Alexandra Miricescu, Brian Mooney, Andreas Bachmair, Emmanuelle Graciet, and Frederica Theodoulou

Review Timeline:

Submission Date:	22-Jun-2023
Editorial Decision:	01-Sep-2023
Revision Received:	26-Jan-2024
Editorial Decision:	14-Mar-2024
Revision Received:	15-Mar-2024
Accepted:	21-Mar-2024

Prof. Frederica L. Theodoulou
Rothamsted Research
Harpenden, Hertfordshire AL5 2JQ
United Kingdom

Dear Dr. Theodoulou:

We have received reviews of your manuscript entitled "BIG enhances protein degradation through the Arg/N-degron pathway to regulate the hypoxia response and suberin deposition in Arabidopsis.." Thank you for submitting your best work to The Plant Cell. The editorial board agrees that the work you describe is substantive, falls within the scope of the journal, and may become acceptable for publication, pending revision and potential re-review. However, I should stress that we are reluctant to see manuscripts undergoing multiple rounds of revision and would be unlikely to offer you more than one chance to satisfy the reviewers.

It appears that all three reviewers are rather positive about the manuscript, but a number of clarifications are required. Reviewer #1 wishes more clarification on the choice of the big-2 allele and on its possible complementation. However, asking for the use of additional putative loss of function alleles of BIG, would not be necessary. This reviewer also wishes more clarification on the experiments reporting how BIG influences the abundance of physiological PRT6/N-degron pathway substrates. Reviewer #2 indicates a number of experiments to clarify the molecular function of BIG1 in submergence stress responses and hypoxic regulation. This reviewer also wished a better clarification of the genetic interaction between PRT6 and BIG, which needs more careful interpretation and a more open appraisal of how their genetic interaction data fits with the proposed model. Finally, all reviewers ask for substantial clarification of the text and figures.

Please contact us if there are ambiguous comments or if you wish to discuss the revision.

Given the nature of the comments, we are offering you 60 days from when we have issued this decision to complete the revision. If a revision is not returned within this time frame, and if you have not been granted an extension, we will withdraw the manuscript, which will leave you free to submit the work elsewhere. If you need an extension, we encourage you to contact us at any point before the 60 days have passed.

When you are ready to submit the revised version, please upload a highlighted copy that indicates all changes made in response to the editor and reviewer recommendations. Include an itemized list of all changes made in response to each of the reviewer's suggestions in the "Response to Reviewers" section; please note that reviewers do not have access to your cover letter, nor was this decision letter shared with them.

Thank you for the privilege of reviewing your work. We look forward to receiving your revised manuscript.

Sincerely,

The Plant Cell Editorial Board

Please note the following:

-The Plant Cell now requires authors to complete and submit an author revisions checklist upon submission of a revised manuscript. The aim of the checklist is to aid authors in preparing a high-quality manuscript, facilitate the review and assessment of revised manuscripts, and help to ensure that journal standards are maintained across the board. If your manuscript is accepted, the completed checklist will be published as supplemental material attached to the article online. Please download a copy of the checklist (pdf fillable form) at this link, for submission with your revised manuscript: https://tpc.msubmit.net/html/Author_Revisions_Checklist.pdf.

-Supplemental materials should be restricted to large datasets and tables, presentation of replicates, and validation of reagents, methods, or genotypes. Any data that are used to support the major claims must be in the main manuscript. Supplemental figure legends must indicate what figure in the main manuscript is supported by the supplemental data presented. Please justify how each of the supplemental figures meet the criteria.

-Sampling methods and nature of "biological replicates" should be described precisely (i.e. different plants, parts of

plants, pooled tissue, independent pools of tissue, sampled at different times, etc), along with a clear description of and rationale for any statistical analyses conducted. The reader should know exactly what was sampled; what forms the basis of the calculation of any means and statistical parameters reported. This is also necessary to ensure that proper statistical analysis was conducted.

-Want to add this revision deadline to your calendar? Click below!

-- Reviewer comments:

Reviewer #1 (Comments for the Author):

Review Zhang et al.

Hongtao Zhang and colleagues have, in a multi-lab collaborative effort, compiled to my knowledge the first manuscript about a possible role of the suggested N-recognin BIG in plant proteostasis. They suggest its role in a possibly novel branch of the N-degron pathways. They describe phenotypes of single and higher order mutants of BIG as well as effects of loss-of-function mutants on protein half-life of engineered N-degron pathway reporters. At the same time, the authors test for changes in abundance of some of the very few known physiological substrates of the N-degron pathways, here they concentrate on the Arg/Cys-N-degron pathway with protein levels of VRN2 and ERFVIIs, and, for ZPR2, they show transcript data. They also test changes in the root transcriptome if BIG was mutated and for the first time associate both PRT6 and BIG with suberin deposition in roots, a both interesting and at the same time physiologically and anatomically relevant and complex process.

I tried to find more information on the used BIG loss-of-function allele big-2 and why the authors selected this (and only this one) line. BIG has more than 17,000 bp that translates to an up to 570 kDa protein and with a T-DNA interruption in big-2 which happens apparently in exon five right in the middle of the gene, a lot of the original sequence (one half or 280 kDa (!) to be precise) remains, amongst it regions encoding several protein-protein interaction domains.
Then, was the line backcrossed before using, if yes, how often and can the authors exclude that additional T-DNA insertions besides the one in BIG are causative for the phenotypes of the big-2 mutant? I am not aware of a complementation, is there one? A proper illustration of big-2 is missing in the supplements with primers and domain binding sites annotated in a gene representation similar to Kasajima et al. Physiol Plant (2007). Is big-2 a loss-of-function mutation and can big-2 be complemented by full length or truncated versions of BIG? Have other alleles of big mutant candidates been tested (there are quite a number of candidate mutants from the collections available)? Have also other alleles been tested and if so, did they show similar phenotypes, e.g. SALK_107817 or SALK_015237 or any of the GABI-Kat lines?

The authors test potential protein interactions using one Arg-initiated bait (the R-TurboID sample) by proximity labelling and found PRT6 and BIG. Alone that this technique worked is remarkable. On top, they found this unexpected dual interaction. However, finding out biochemical properties of the huge BIG protein will be more than challenging.

The authors close with a speculative model for PRT6 and BIG acting jointly together in the N-degron pathways in plants and regulating known physiological routes such as hypoxia response. Moreover, they suggest that BIG might serve as a recognition component of the Arg/N-degron pathways and possibly other proteostatic mechanisms in form of an interaction platform. It is remarkable again that protein accumulation effects documented by Westerns are increased in the prt6 big double mutant compared to the prt1 single mutant whereas big alone is indistinguishable from the wild type. The authors explain this with an additive effect.

I find this work very exciting and timely, as in the proteostasis community, colleagues are discussing frequently about novel candidates for N-recognins and especially about the mystic humungous protein BIG which has, if mutated, misregulations in so many and so diverse physiological functions. Taken together, this makes it a tough cookie to work on and this manuscript, if published after some clarifications will eventually have been made, will soon turn into a paper that will be cited a decent number of times in various contexts of plant biology and general proteostasis.

Major points:
- The big mutant description is confusing: SALK_045560 (their big-2) is also called big-1 in pevious publications and already in 2014, authors described more alleles (Ivanova et al. 2014; what about SALK_145141 this study also discussed?)
- l. 70 ff. to my knowledge, there is no data on PRT6 really acting as a Ub ligase, i.e. attaching Ub to a protein substrate, especially not in the cited papers close by in this paragraph. So, I prefer to see this written more carefully and, for example, still call PRT6 a "candidate" Ub ligase or similar or add the information that there is good reasons for thinking that it is one but that there is, so far, no biochemical evidence for this.
- On the other hand, there is such data available for PRT1, which the authors also call a Ub E3 ligase, but they might

have missed it's biochemical description e.g. in Mot et al. New Phytol (2018).
- l.100 f. "N-terminally arginylated ERFVIIs are then recognised by PRT6" is a common assumption but has not yet been shown molecularly. It appears like this in many schemes and summaries of the Arg/Cys-N-degron pathway but I haven't heard about interaction studies etc. and also PRT6 protein preparations, I have never heard of. So, also this part should - in my eyes - be more carefully written in an assumptive way to stick to the science made/published.
- l. 1191 f.: I find the title of Fig. 3 ("Regulation of the hypoxia response by BIG requires arginylation and ERFVIIs") far fetched. The authors tested and documented changes in expression of ADH1 and PGB1 also in higher order mutants where ate1/2 was introgressed, but they do not test for actual "arginylation" as the title suggests.
- l. 202 ff. I can appreciate that HRE2-HA is more abundant in the prt6 big double mutant compared to the prt6 alone which is in line with the title of Fig. 2 ("BIG influences the abundance of physiological PRT6/N-degron pathway substrates") and this might also be the case for the VRN2:GUS construct. But these experiments are delicate and require many controls. The controls done are not described in the caption or methods part and also information on replicates and reproducibility are lacking. How many independent transgenic lines demonstrated the relevant phenotypes on western blot or GUS histostaining levels? We all know that between different transformants within the same genetic background, we usually expect to have high variabilities.
- Fig. 2 why is there no prt6 big double mutant shown in D and G (for RAP2.3-HA)? In G only prt6 -/- big +/- appears but not the double homozygous one although it exists and was shown in A and Fig. 3.

Minor points:
- l. 158 ff. it is unclear to me if the stable control (M-GUS) used together with the DHFR-Ub-X-GUS system from Garzón et al. (2007) is also a DHFR-Ub-X-GUS fusion itself or if it was a regular Methionine initiated stable control lacking the initiator part of the Ub fusion system (DHFR-Ub moiety). The same construct is shown in Supp. Fig. 1 B and here, it becomes apparent that it is likely to have a different layout. If it was Met-initiated "X-GUS", it is crucial, if the Met will be cleaved or not and what eventually would be the neo-N-terminal amino acid after Met excision... I strongly recommend to check all constructs again and verify that they are correctly explained.
- Supp. Fig. 2A: labeling unclear, are the two left and two right rosettes Col-0 and big-2 whereas in the center, we see two different genotypes?

Reviewer #2 (Comments for the Author):

In their study, Zhang et al. present biochemical data about the role of the UBR-domain protein BIG of Arabidopsis in the Arg/N-degron pathway. This report is valuable and timely, in that it provides the first information on the molecular mechanisms of action of BIG, a protein involved in a diverse array of physiological responses. Having observed small effect on PRT6/NDP (N-degron pathway) substrates, the Authors concluded that BIG participation in that pathway is not likely to explain all phenotypes described for big mutants, and propose that BIG may also be involved in proteasomal degradation of non-NDP substrates and autophagy (see l. 434 ff., Fig. 6 legend), suggesting a direction for future investigations. This hypothesis is agreeable and is based on a well-defined set of experiments to provide sufficient support to it.
The paper is enjoyable, well written, all figures are very clear and presented in a logical way. As the only comments on the data presentation, I found the green crosses in Fig. 1 not visible enough, and the table in Fig. 4A unnecessary (it does not add anything to the description given at l. 284). One reference is missing from l. 329: a hierarchical network of MYB transcription factors controlling suberin deposition has been first described by Shukla et al. PNAS 2021 (doi.org/10.1073/pnas.2101730118).
My main observations on the manuscript, as follows, are related to the physiological implications of BIG involvement in the Arg/NDP and to VRN2 role in hypoxic gene regulation.
1. Fig. 2 and 3 overall show that, in seedlings, VRN2 was regulated in a similar fashion and to a similar extent as RAP2.3 by PRT6 and PRT6+BIG. Consequently, VRN2 also appears in a final scheme (Fig. 4F). Its connection to the regulation of anaerobic genes and their products was explored in this study for the first time. More abundant ADH protein was detected in vrn2 prt6 double mutants than in prt6 single mutants (Fig. 3F) and possibly also PGB1, but the blot is saturated for those bands; higher PGB1 transcript was measured in the double mutant, while the difference in ADH expression was not significant (Fig. 3E). This evidence is suggestive of a negative role of VRN2 in the regulation of hypoxic gene expression. The panel of anaerobic genes tested could be easily expanded to some more markers, chosen among core anaerobic genes, to consolidate these findings, and to understand whether VRN2 may mediate widespread regulation on anaerobic genes, or rather have a specific role on ADH and PGB1.
2. The claims made on the contribution of hypoxia survival of the novel regulation described in the study are based on chlorophyll retention in seedlings as the sole tolerance test. Root tip survival in big-2 prt6 seedlings or submergence/waterlogging rosette survival should be further tested, to understand the physiological impact of BIG participation to hypoxic regulation.
3. To understand the influence of BIG on physiological NDP substrates, the Authors combined big-2 and prt6 mutations with a 35S:RAP2.3-HA construct. The phenotypes associated with the two individual single mutant backgrounds were not commented. The top part of Suppl. Fig. 2A shows fairly similar plant appearance between mutants and wild-type, at

a late flowering stage: since ERFVII stabilization has been associated with strong developmental phenotypes, in particular for RAP-type ERFVIIs, it is surprising that prt6-5 plants did not develop any. RAP2.3 accumulation was indeed promoted in that background (Fig. 2D), at least in seedlings. One possibility is that the PRT6/N-degron pathway loses effectiveness at degrading its ERFVII substrates as plants age, as observed by Giuntoli et al. PCE 2017 (doi.org/10.1111/pce.13037). In this case, a difference between wild-type or big-2 and prt6 would be expected to be visible at young vegetative stages, but not in adult plants, with prt6 plants showing at the young stage some of the traits manifested by the double mutant background plants. Could the Authors comment on these aspects, or document the progression of development of 35S:RAP2.3-HA plants before the flowering phase?
4. On the contrary, big-2 prt6 35S:RAP2.3-HA displayed evident phenotypes, among which smaller rosettes, twisted leaves with abnormal surfaces, and reproductive defects. Similar phenotypic outcome has been associated before with the expression of an N-terminal truncation of RAP2.12, as reported in the same study cited previously (Giuntoli et al. PCE 2017), reasonably due to highly effective stabilization of the protein. There, some defect in cuticle deposition has been observed and linked to the leaf phenotype of the over-expressor. Given the similarities between cuticle and suberin biosynthesis, those previous observations should be referenced in the discussion (l. 373 may be a proper place, for instance). Overall, deltaN-RAP2.12, quadruple pco mutants (Weits et al., 2023; doi.org/10.1111/pce.14440) and the data presented in this manuscript align well to suggest that high constitutive presence of ERFVII proteins prevents proper biosynthesis of surface polymers and suberin.
5. The analysis in Fig. 5A-B led the Authors to conclude that both ERFVII-dependent and BIG-dependent regulation occurs in roots during suberin deposition. It would be interesting to extend the analysis of the genotypes in Fig. 5B to the expression of genes from Fig. 5A that are shared with cutin biosynthetic pathway (such as LACS2, CYP86A, or GPATs), to be measured in leaves of soil grown plants, to understand whether or not analogous distinct regulation may take place in the aerial organs (and connect to the previous point).
6. The observation that the PRT6 pathway and BIG are involved in suberin deposition in roots seems to have straightforward links with the physiological adaptations of the plant to flooded conditions. For instance, it may not be too far-fetched to speculate that the hypoxic response also includes the regulation of root permeability to water and solutes when the soil is flooded. The findings related to suberin synthesis are highlighted in the title and summary, but were eventually barely discussed. I believe that the readers may appreciate being guided through the perspectives or future directions of these findings a bit more.
Regarding the genetic interaction between PRT6 and BIG, it is defined as "synergistic" in several instances, however this term does not seem completely accurate, since big-2 mutation alone had no effects on the (molecular) phenotypes observed, to be added to prt6 effect in the combined mutant. , Synergy would imply the phenotype made by the two null mutations to be greater than the sum of their individual effects (doi:10.1016/j.tig.2009.06.004). PRT6 and BIG seem to rather have an epistatic relationship. A speculative molecular mechanism underlying this genetic interaction is contained in Fig. 6, whereby the gathered information on BIG participation in proteasome functions is summarized. The model makes sense, but it is still hard to figure out why BIG removal from the UPS complex should become relevant only after PRT6 is missing (if anything, one would expect some effect from BIG removal, rather than enhanced effect to PRT6 removal).
Finally, I have two additional questions:
• Clarification on the blots with the model substrates used in Fig. 1: the reference and reporter protein should be present in stoichiometric amounts, when the latter is fully stabilized, but the reference band is always much weaker in the blots. It this due to the different amounts of HA repetitions?
• Among the BIG mutants mentioned in the introduction, the only one missing is the one used in this study, big-2. It is unclear to me whether this study is the first one characterizing the mutant phenotype, or there may be more information to mention on it, from the source paper mentioned in Suppl Tab. 1 or subsequent studies.

Reviewer #3 (Comments for the Author):

This manuscript describes functions of the BIG protein in Arabidopsis using genetics and biochemical methods. BIG is a member of the conserved UBR protein family in plants, yeast and animals that is involved in the recognition of different N termini of proteins and facilitation of their degradation. Some plant UBRs have been characterised such as PRT6 that targets basic N-termini, but mechanisms that target other N-termini types are not well characterised in plants. The N degron pathway maintains protein levels in a wide variety of developmental and environmental responses, therefore it is a very important and relatively uncharacterised aspect of plant biology.
Mutants in the BIG protein, a UBR homolog, have a wide range of phenotypes in Arabidopsis but it's potential role in degron pathways is not known. Here the authors show a role for BIG in protein degradation and describe its function with respect to another UBR protein PRT6 and another N- degron recognin PRT1. New processes influenced by these protein degradation systems such as suberin formation have been described.

General comments

The manuscript covers an important but under-characterised area of plant biology. The approaches taken are appropriate and insightful, working to the biochemical strengths of the authors. The work is thorough (includes VRN2

and hypoxia proteins) and mostly well described, and the conclusions are supported by the evidence.

Specific comments
Introduction

It could be shortened a little- it's quite review-like in places especially the N degron system.
L115 how many UBR family proteins are there in Arabidopsis?
Provide a more detailed description of BIG function from work in other systems and what could be expected from this knowledge

Results

L162-164. The reader needs a clearer description of what the type 1 substrate and type 2 substrates are and what the stable M-GUS is exactly. The Introduction describes type 1 degrons but not type 2 as far as I can make out. The authors need to make sure that they pitch the experimental results as clearly as possible to the general plant reader.
Figure 1. Need to describe R- and F- GUS and M-GUS precisely in the Figure and Legend. It's not clear enough to the non-expert. Components of Fig S1B, C need to be in the main Figure for direct comparison of M-GUS with the R- and M-GUS experiments.
L169. Re-phrase "removal of N-recognin function" sentence to make it clearer.
L172-174. Fig 1 C and D show the absence of the precursor HA-GUS fusions in big1-2 relative to the HA-DHFR stability protein. Isn't it clearer to state that the GUS fusions require BIG function for their stability? I don't understand how it's possible to conclude that BIG mediates R- and F-GUS degradation? This whole section describing Fig 1C and 1D needs to be described more clearly. Are big mutant plants relatively sick and have pleiotropic effects eg Fig 2B? They don't look sick from for Fig 1E.
L186. To comprehend Figure 1F a diagram of the Turbo ID components is needed in the Figure. The reader should not have to rifle through supplemental Figures for key information.
Figure 2, what do the A, B, C, D letters mean on the graphs with respect to significance levels?
L205. Need to describe specifically the role of ERFVIIs in the hypoxia response.

Discussion

L350. The sentence starting "Notably..." should be made more accessible by describing the functions of ATE and PRT6 proteins, not the mutants. The general reader needs this information in front of them.
Use knowledge from other systems to understand the interactions of BIG family proteins and PRT6 eg do they interact?. In particular explain the phenotype of increased substrate turnover in big mutants, and reduced turnover in big prt6 mutants. In the absence of BIG function does PRT6 target the substrate protein for higher levels of degradation?

Reviewing Editor comments:
Overall, some more experimentation and much more clear description are required to reach to necessary standard for Plant Cell.

Response to Referees
Zhang et al.

Dear Dr. Theodoulou:

We have received reviews of your manuscript entitled "BIG enhances protein degradation through the Arg/N-degron pathway to regulate the hypoxia response and suberin deposition in Arabidopsis.." Thank you for submitting your best work to The Plant Cell. The editorial board agrees that the work you describe is substantive, falls within the scope of the journal, and may become acceptable for publication, pending revision and potential re-review. However, I should stress that we are reluctant to see manuscripts undergoing multiple rounds of revision and would be unlikely to offer you more than one chance to satisfy the reviewers.

It appears that all three reviewers are rather positive about the manuscript, but a number of clarifications are required.
Reviewer #1 wishes more clarification on the choice of the big-2 allele and on its possible complementation. However, asking for the use of additional putative loss of function alleles of BIG, would not be necessary. This reviewer also wishes more clarification on the experiments reporting how BIG influences the abundance of physiological PRT6/N-degron pathway substrates.
Reviewer #2 indicates a number of experiments to clarify the molecular function of BIG1 in submergence stress responses and hypoxic regulation. This reviewer also wished a better clarification of the genetic interaction between PRT6 and BIG, which needs more careful interpretation and a more open appraisal of how their genetic interaction data fits with the proposed model.
Finally, all reviewers ask for substantial clarification of the text and figures.

Please contact us if there are ambiguous comments or if you wish to discuss the revision.

Given the nature of the comments, we are offering you 60 days from when we have issued this decision to complete the revision. If a revision is not returned within this time frame, and if you have not been granted an extension, we will withdraw the manuscript, which will leave you free to submit the work elsewhere. If you need an extension, we encourage you to contact us at any point before the 60 days have passed.

When you are ready to submit the revised version, please upload a highlighted copy that indicates all changes made in response to the editor and reviewer recommendations. Include an itemized list of all changes made in response to each of the reviewer's suggestions in the "Response to Reviewers" section; please note that reviewers do not have access to your cover letter, nor was this decision letter shared with them.

Thank you for the privilege of reviewing your work. We look forward to receiving your revised manuscript.

Sincerely,

The Plant Cell Editorial Board

Please note the following:</>

-The Plant Cell now requires authors to complete and submit an author revisions checklist upon submission of a revised manuscript. The aim of the checklist is to aid authors in preparing a high-quality manuscript, facilitate the review and assessment of revised manuscripts, and help to ensure that journal standards are maintained across the board. If your manuscript is accepted, the completed checklist will be published as supplemental material attached to the article online. Please download a copy of the checklist (pdf fillable form) at this link, for submission with your revised manuscript: https://tpc.msubmit.net/html/Author_Revisions_Checklist.pdf.

-Supplemental materials should be restricted to large datasets and tables, presentation of replicates, and validation of reagents, methods, or genotypes. Any data that are used to support the major claims must be in the main manuscript. Supplemental figure legends must indicate what figure in the main manuscript is supported by the supplemental data presented. Please justify how each of the supplemental figures meet the criteria.

-Sampling methods and nature of "biological replicates" should be described precisely (i.e. different plants, parts of plants, pooled tissue, independent pools of tissue, sampled at different times, etc), along with a clear description of and rationale for any statistical analyses conducted. The reader should know exactly what was sampled; what forms the basis of the calculation of any means and statistical parameters reported. This is also necessary to ensure that proper statistical analysis was conducted.

-Want to add this revision deadline to your calendar? Click below!

-- Reviewer comments:

Reviewer #1 (Comments for the Author):

Review Zhang et al.

Hongtao Zhang and colleagues have, in a multi-lab collaborative effort, compiled to my knowledge the first manuscript about a possible role of the suggested N-recognin BIG in plant proteostasis. They suggest its role in a possibly novel branch of the N-degron pathways. They describe phenotypes of single and higher order mutants of BIG as well as effects of loss-of-function mutants on protein half-life of engineered N-degron pathway reporters. At the same time, the authors test for changes in abundance of some of the very few known physiological substrates of the N-degron pathways, here they concentrate on the Arg/Cys-N-degron pathway with protein levels of VRN2 and ERFVIIs, and, for ZPR2, they show transcript data. They also test changes in the root transcriptome if BIG was mutated and for the first time associate both PRT6 and BIG with suberin deposition in roots, a both interesting and at the same time physiologically and anatomically relevant and complex process.

I tried to find more information on the used BIG loss-of-function allele big-2 and why the authors selected this (and only this one) line. BIG has more than 17,000 bp that translates to an up to 570 kDa protein and with a T-DNA interruption in big-2 which happens apparently in exon five right in the middle of the gene, a lot of the original sequence (one half or 280 kDa (!) to be precise) remains, amongst it regions encoding several protein-protein interaction domains.
Then, was the line backcrossed before using, if yes, how often and can the authors exclude that additional T-DNA insertions besides the one in BIG are causative for the phenotypes of the big-2 mutant? I am not aware of a complementation, is there one? A proper illustration of big-2 is missing in the supplements with primers and domain binding sites annotated in a gene representation similar to Kasajima et al. Physiol Plant (2007). Is big-2 a loss-of-function mutation and can big-2 be complemented by full length or truncated versions of BIG? Have other alleles of big mutant candidates been tested (there are quite a number of candidate mutants from the collections available)? Have also other alleles been tested and if so, did they show similar phenotypes, e.g. SALK_107817 or SALK_015237 or any of the GABI-Kat lines?

Thank you for these suggestions which we are agree are important considerations for this study.

big-2 is the SALK_045560 T-DNA insertion line, originally described in Kasajima et al., 2007. We have included a schematic of the gene and protein showing the position of the T-DNA in the revised manuscript (new Supplemental Figure 1) and referenced this early in the Results section.

Our study was started several years ago, and we chose *big-2* for the pragmatic reason that it was available from the stock centre and potential collaborators agreed to work with the same line. As it has a severe morphological phenotype, we considered that it was likely to be a loss of function allele. The reviewer is correct that the T-DNA is inserted about halfway through the gene and our RNA-seq data indicate that *big-2* expresses a truncated transcript, albeit at a much lower level than wild type. The truncated transcript could potentially produce a protein containing the UBR box and this is supported by our unpublished proteomic analysis of the *big-2* allele. However, it is impossible to say whether the truncated protein would be correctly folded. Importantly, information from Drosophila and mammalian homologues of BIG indicate that a truncated protein is likely to be non-functional in the N-degron pathway because it lacks the recently identified hemi-RING E3 ligase domain (Barnsby-Greer et al., 2024 https://doi.org/10.1038/s41594-023-01192-4).

In theory, a mutant allele that produces a truncated protein could be a dominant negative but plants heterozygous for *big-2* look similar to wild type (in fact, the small size of *big-2* homozygotes is an extremely useful marker when analysing cross progeny) suggesting that there is no dominant negative effect. Images of *big-2* heterozygotes are now shown in Supplemental Figure 1.

Owing to the enormous size of the BIG cDNA and genomic clones, we did not attempt complementation. In the manuscript, we report 12 different mutant combinations or transgenic lines that contain the *big-2* allele; this represents a large body of work, and we did not have the resources to test multiple alleles. Nevertheless, we agree that it is important to rule out the possibility that a second T-DNA (or other genetic variant) could be causative for the N-degron pathway-associated phenotypes. The *big*-2 T-DNA insertion line was originally obtained from the Nottingham Arabidopsis Stock Centre and made homozygous. Whilst it was not back-crossed to Col-0 before use, we made many crosses in the course of our study and did not observe segregation of the N-degron pathway phenotype away from the *big-2* T-DNA insertion. However, our recent re-sequencing the *big-2* allele revealed the presence of a second T-DNA in the final exon of AT3G61680 which encodes plastid localised phospholipase A1 (PLIP1) involved in seed oil biosynthesis (Wang et

al., 2017 https://do.org/10.1105/tpc.17.00397). We have designated this allele *plip1-3* as two T-DNA alleles were published previously (Wang et al., 2017). We consider it unlikely that a lesion in *PLIP1* would lead to stabilisation of N-degron pathway substrates. However, we designed a PCR assay to detect the presence of the *plip1-3* T-DNA in our seed stocks and in archived DNA samples used for genotyping during the construction of crosses and transgenics. *BIG* and *PLIP1* are at opposite ends of chromosome 3 and consequently, in most cases, we were able to work with material in which the *PLIP* T-DNA had been segregated out. An important exception is the RNA-seq study which unfortunately was performed with *big-2* seed homozygous for *plip1-3*. Whilst it is possible that *plip1-3* influences some of the transcript changes in *big-2* and *prt6-5 big-2*, the headline conclusions from the transcriptome data are consistent with other data reported in the manuscript. For additional assurance, we repeated key q-RT-PCR experiments using material lacking *plip1-3.* This clearly demonstrated that the enhanced expression of hypoxia-responsive genes in *prt6-1 big-2* is independent of *plip1-3*, as is the altered expression of suberin genes (Supplemental Figure 13). In light of these data, we consider that the conclusions reported in the manuscript are robust. These points are now incorporated in the revised manuscript.

The authors test potential protein interactions using one Arg-initiated bait (the R-TurboID sample) by proximity labelling and found PRT6 and BIG. Alone that this technique worked is remarkable. On top, they found this unexpected dual interaction. However, finding out biochemical properties of the huge BIG protein will be more than challenging.

Biochemical analysis of BIG is indeed challenging, and this is one reason why we took a principally genetic approach for this study.

The authors close with a speculative model for PRT6 and BIG acting jointly together in the N-degron pathways in plants and regulating known physiological routes such as hypoxia response. Moreover, they suggest that BIG might serve as a recognition component of the Arg/N-degron pathways and possibly other proteostatic mechanisms in form of an interaction platform. It is remarkable again that protein accumulation effects documented by Westerns are increased in the prt6 big double mutant compared to the prt1 single mutant whereas big alone is indistinguishable from the wild type. The authors explain this with an additive effect.

I find this work very exciting and timely, as in the proteostasis community, colleagues are discussing frequently about novel candidates for N-recognins and especially about the mystic humungous protein BIG which has, if mutated, misregulations in so many and so diverse physiological functions. Taken together, this makes it a tough cookie to work on and this manuscript, if published after some clarifications will eventually have been made, will soon turn into a paper that will be cited a decent number of times in various contexts of plant biology and general proteostasis.

Thank you for these positive comments!

Major points:
- The big mutant description is confusing: SALK_045560 (their big-2) is also called big-1 in previous publications and already in 2014, authors described more alleles (Ivanova et al. 2014; what about SALK_145141 this study also discussed?)

Please see the response above. *big-2* is the correct name for the allele, based on publication precedence. However, to provide clarity, we have now included a figure showing the position of the T-DNA insertions in *big-1* and *big-2* (and their correct stock numbers) in Supplemental Figure 1.

- l. 70 ff. to my knowledge, there is no data on PRT6 really acting as a Ub ligase, i.e. attaching Ub to a protein substrate, especially not in the cited papers close by in this paragraph. So, I prefer to see this written more carefully and, for example, still call PRT6 a "candidate" Ub ligase or similar or add the information that there is good reasons for thinking that it is one but that there is, so far, no biochemical evidence for this.

The reviewer is correct that there is no direct biochemical evidence for PRT6 acting as a ubiquitin ligase (although it is a very reasonable proposition), therefore we have modified the text accordingly with the term “candidate”.

- On the other hand, there is such data available for PRT1, which the authors also call a Ub E3 ligase, but they might have missed it's biochemical description e.g. in Mot et al. New Phytol (2018).

We apologise for this omission and have cited Mot et al., 2018 accordingly, making an explicit reference to the E3 activity of PRT1.

- l.100 f. "N-terminally arginylated ERFVIIs are then recognised by PRT6" is a common assumption but has not yet been shown molecularly. It appears like this in many schemes and summaries of the Arg/Cys-N-degron pathway but I haven't heard about interaction studies etc. and also PRT6 protein preparations, I have never heard of. So, also this part should - in my eyes - be more carefully written in an assumptive way to stick to the science made/published.

We have modified the text to read: “N-terminally arginylated ERFVIIs are then thought to be recognised by PRT6, which targets the proteins for proteasomal degradation”.

- l. 1191 f.: I find the title of Fig. 3 ("Regulation of the hypoxia response by BIG requires arginylation and ERFVIIs") far fetched. The authors tested and documented changes in expression of ADH1 and PGB1 also in higher order mutants where ate1/2 was introgressed, but they do not test for actual "arginylation" as the title suggests.

To address this valid point, we have edited the figure title to read: "Regulation of the hypoxia response by BIG requires ATE1/2 and ERFVIIs". (This is now Figure 4 in the revised manuscript).

- l. 202 ff. I can appreciate that HRE2-HA is more abundant in the prt6 big double mutant compared to the prt6 alone which is in line with the title of Fig. 2 (“BIG influences the abundance of physiological PRT6/N-degron pathway substrates”) and this might also be the case for the VRN2:GUS construct. But these experiments are delicate and require many controls. The controls done are not described in the caption or methods part and also information on replicates and reproducibility are lacking. How many independent transgenic lines demonstrated the relevant phenotypes on western blot or GUS histostaining levels? We all know that between different transformants within the same genetic background, we usually expect to have high variabilities.

When we embarked on this study, there were relatively few available transgenic lines expressing N-degron pathway substrates in the *prt6* background, which limited the possibility to use independent transgenic lines for each reporter. This is partly because we and others have found it difficult to obtain lines in which substrates can be detected by immunoblotting, perhaps due to the deleterious effects of extreme ERFVII stabilisation. These experiments are also made more challenging by the slow development and low seed yield of *prt6 big-2* double mutants. Originally, we had two independent *prt6-5*/35S::HRE2-HA lines with different expression levels, and crossed both to *big-2* but could only recover the homozygous *prt6-5 big-2* progeny from one of these, despite several crossing attempts. This may be because the *HRE2* transgene is inserted close to the *big-2* T-DNA or could reflect extreme stabilisation of HRE2 in the double mutant background for this event, as we encountered for RAP2.3.

So, we had to be pragmatic and work with single lines. However, we would like to draw the reviewer's attention to the fact that we have shown increased abundance of five different substrates in *prt6 big-2* mutants (R-GUS, F-GUS, R-LUC, HRE2 and VRN2), all of which of course are independent transgenics, which gives us confidence that our conclusions are robust. Moreover, the data were generated in two different labs independently (R-LUC data in EG's lab; X-GUS and physiological substates in FT's lab). With the exception of Fig 3G (where the number of seeds was limiting), all experiments were repeated at least once and some many times. For histochemistry, ~15 seedlings were used for each experiment and five representative seedlings were rearranged for photography. These points are now incorporated in the revised manuscript.

Regarding controls, we are unclear what the referee means here; we do, however, show Ponceau staining of immunoblots as loading controls in all figures. We prefer these to probing the blot with antibodies to "housekeeping" proteins because some of these are also differentially expressed in the mutants. We also show that the increased levels of N-degron pathway substrates in *prt6 big-2* are not due to increases in their transcripts.

- Fig. 2 why is there no prt6 big double mutant shown in D and G (for RAP2.3-HA)? In G only prt6 -/- big +/- appears but not the double homozygous one although it exists and was shown in A and Fig. 3.

The double homozygous *prt6 big-2* mutants presented in Fig 2A are expressing 35S::HRE2-HA and those in Fig 3 are <u>untransformed plants</u>. Fig 2D and 2G show plants of different genotypes expressing 35S::RAP2.3-HA. As explained in the text (Results lines 267-273; Discussion lines 448-455; relevant text copied below), whilst we could obtain double homozygous *prt6-5 big-2* plants expressing 35S::RAP2.3-HA, they did not set seed, so we couldn't generate multiple seedlings required for immunoblotting. Moreover, it was not possible to make a meaningful comparison of mature tissue from plants of different genotypes expressing RAP2.3 due to the extreme morphological phenotype of *prt6-5 big-2* over-expressers (Supplemental. Fig 4).

We interpret this result as an effect of extreme RAP2.3 stabilisation on plant development. Apparently, stabilisation of HRE2 is less deleterious, which is consistent with its more secondary role in hypoxia signalling (Sasidharan and Mustroph 2011 https://doi.org/10.1105/tpc.111.093880). Furthermore, the expression level of HRE2-HA is lower than that of RAP2.3-HA, as judged from immunoblots of crude protein extracts from the transgenic lines probed with anti-HA, ADH and PGB1 antibodies. This image is now shown in Supplemental Figure 6.

Relevant excerpts from the manuscript text:

"*prt6-5 big-2* double mutant lines expressing RAP2.3-HA exhibited curled leaves and generally stunted growth, and flowering was extremely delayed with only a short primary bolt produced (Supplemental Fig. 2A). We were unable to recover seeds from these plants; dissection of flowers revealed incompletely elongated stamens that did not mature or release pollen (Supplemental Fig. 2B). Therefore, we analysed pooled seedlings homozygous for *prt6-5* but segregating for *big-2*."

"It was not possible to test unequivocally whether RAP2.3 is similarly stabilised in *prt6-5 big-2* seedlings since double mutant plants expressing 35S::RAP2.3-HA did not set seed (Supplemental Fig. 2). However, this observation, together with the partial rescue of delayed flowering and reduced fertility of *prt6-5 big-2* plants by genetic removal of RAP function (Supplemental Fig. 3) strongly suggests that RAP2.3 is also hyperstabilised in the double mutant and that extreme stabilisation of RAP-type ERFVII transcription factors is deleterious to growth and reproduction."

Minor points:
- l. 158 ff. it is unclear to me if the stable control (M-GUS) used together with the DHFR-Ub-X-GUS system from Garzón et al. (2007) is also a DHFR-Ub-X-GUS fusion itself or if it was a regular Methionine initiated stable control lacking the initiator part of the Ub fusion system (DHFR-Ub moiety). The same construct is shown in Supp. Fig. 1 B and here, it becomes apparent that it is likely to have a different layout. If it was Met-initiated "X-GUS", it is crucial, if the Met will be cleaved or not and what eventually would be the neo-N-terminal amino acid after Met excision... I strongly recommend to check all constructs again and verify that they are correctly explained.

We apologise for the lack of clarity. The M-GUS stable control is also a DHFR-Ub-X-GUS fusion (as in Garzón et al., 2007), which should be clearer in the revised version of Fig. 1 which shows M-, R- and F-GUS side by side.

Methionine amino peptidases work co-translationally and only cleave Met1 when it is adjacent to a small residue at position 2, with the specificity: M/AGPSTV (Meinnel and Giglione, 2022 https://doi.org/10.1093/jxb/erac290). The residue following "X" in the X-GUS constructs is Arg and therefore the Met revealed by DUB-catalysed cleavage of DHFR-Ub would not be processed further.

- Supp. Fig. 2A: labeling unclear, are the two left and two right rosettes Col-0 and big-2 whereas in the center, we see two different genotypes?

The six plants within the white box are all rosettes of *prt6-5 big-2* expressing 35S::RAP2.3-HA. We apologise that this was unclear and have amended the legend accordingly.

Reviewer #2 (Comments for the Author):

In their study, Zhang et al. present biochemical data about the role of the UBR-domain protein BIG of Arabidopsis in the Arg/N-degron pathway. This report is valuable and timely, in that it provides the first information on the molecular mechanisms of action of BIG, a protein involved in a diverse array of physiological responses. Having observed small effect on PRT6/NDP (N-degron pathway) substrates, the Authors concluded that BIG participation in that pathway is not likely to explain all phenotypes described for big mutants, and propose that BIG may also be involved in proteasomal degradation of non-NDP substrates and autophagy (see l. 434 ff., Fig. 6 legend), suggesting a direction for future investigations. This hypothesis is agreeable and is based on a well-defined set

of experiments to provide sufficient support to it.
The paper is enjoyable, well written, all figures are very clear and presented in a logical way.

Thank you for these positive comments!

As the only comments on the data presentation, I found the green crosses in Fig. 1 not visible enough, and the table in Fig. 4A unnecessary (it does not add anything to the description given at l. 284).

Thank you for these suggestions. Fig 1 has been edited to improve legibility and the table in Fig. 4A removed.

One reference is missing from l. 329: a hierarchical network of MYB transcription factors controlling suberin deposition has been first described by Shukla et al. PNAS 2021 (doi.org/10.1073/pnas.2101730118).

Apologies for this oversight; this reference has now been cited in the revised manuscript.

My main observations on the manuscript, as follows, are related to the physiological implications of BIG involvement in the Arg/NDP and to VRN2 role in hypoxic gene regulation.
1. Fig. 2 and 3 overall show that, in seedlings, VRN2 was regulated in a similar fashion and to a similar extent as RAP2.3 by PRT6 and PRT6+BIG. Consequently, VRN2 also appears in a final scheme (Fig. 4F). Its connection to the regulation of anaerobic genes and their products was explored in this study for the first time. More abundant ADH protein was detected in vrn2 prt6 double mutants than in prt6 single mutants (Fig. 3F) and possibly also PGB1, but the blot is saturated for those bands; higher PGB1 transcript was measured in the double mutant, while the difference in ADH expression was not significant (Fig. 3E). This evidence is suggestive of a negative role of VRN2 in the regulation of hypoxic gene expression. The panel of anaerobic genes tested could be easily expanded to some more markers, chosen among core anaerobic genes, to consolidate these findings, and to understand whether VRN2 may mediate widespread regulation on anaerobic genes, or rather have a specific role on ADH and PGB1.

Thank you for this suggestion. We have analysed another six of the Core 49 hypoxia genes (*ACO1, HRA, HUP40, PCO1, PCO2, PDC2*) and have included the data in Supplemental Figure 8. Although there is a trend towards greater abundance in *prt6 vrn2* compared to *prt6*, this was not statistically significant for the additional genes tested. Therefore, the effect of VRN2 seems to be specific for *ADH* and *PGB1*. This has been incorporated in the revised text.

2. The claims made on the contribution of hypoxia survival of the novel regulation described in the study are based on chlorophyll retention in seedlings as the sole tolerance test. Root tip survival in big-2 prt6 seedlings or submergence/waterlogging rosette survival should be further tested, to understand the physiological impact of BIG participation to hypoxic regulation.

Thank you for this suggestion. We also considered a root tip survival assay to complement the chlorophyll retention assays and indeed did several experiments prior to the original submission. However, we encountered two problems with these experiments. Firstly, we struggled to detect increased tolerance of *prt6* vs. wild type; using hypoxia jars, the window of hypoxia treatment varies

between experiments, so despite conducting multiple experiments to optimise the conditions we considered that we did not have a good baseline for analysing *prt6 big-2*. The second problem is that in this experimental set-up, we are trying to correlate increased expression of hypoxia responsive genes with hypoxia tolerance against a backdrop of a pleiotropic mutant phenotype. *big-2* has short primary roots and almost no laterals, making it very hard to interpret any assay involving root growth. In our hands, we were only able to detect increased root tip survival in *prt6* roots over-expressing RAP2.3, in which there is a very high level of hypoxia gene expression compared to untransformed *prt6* and *prt6 big-2*. These data are presented in Supplemental Fig. 6 (immunoblot) and in Supplemental Fig. 7A (root tip survival). The small root phenotype of *big-2* and *prt6 big-2* was also a confounding factor in waterlogging experiments, where we found that these mutants were sensitive to waterlogging under the conditions tested. These data are presented in Supplemental Fig. 7B.

3. To understand the influence of BIG on physiological NDP substrates, the Authors combined big-2 and prt6 mutations with a 35S:RAP2.3-HA construct. The phenotypes associated with the two individual single mutant backgrounds were not commented. The top part of Suppl. Fig. 2A shows fairly similar plant appearance between mutants and wild-type, at a late flowering stage: since ERFVII stabilization has been associated with strong developmental phenotypes, in particular for RAP-type ERFVIIs, it is surprising that prt6-5 plants did not develop any. RAP2.3 accumulation was indeed promoted in that background (Fig. 2D), at least in seedlings. One possibility is that the PRT6/N-degron pathway loses effectiveness at degrading its ERFVII substrates as plants age, as observed by Giuntoli et al. PCE 2017 (doi.org/10.1111/pce.13037). In this case, a difference between wild-type or big-2 and prt6 would be expected to be visible at young vegetative stages, but not in adult plants, with prt6 plants showing at the young stage some of the traits manifested by the double mutant background plants. Could the Authors comment on these aspects, or document the progression of development of 35S:RAP2.3-HA plants before the flowering phase?

Whilst *prt6-5* and Col-0 plants expressing 35S::RAP2.3-HA look similar at the late flowering stage, we do indeed see differences in seedlings. They have curled cotyledons, lack lateral roots and the true leaves develop more slowly. Overall, their vegetative development is slower, and they take longer to flower, indicative of the impact of constitutively stabilised RAP2.3. As the referee suggests, the more pronounced phenotype in seedlings is consistent with the reduced impact of the PRT6/N-degron pathway in older plants as reported in Guintoli et al., 2017. We have revised the Supplemental figures to include representative seedling images (new Supplemental Fig. 3) and images showing the delayed development (new Supplemental Fig. 4) and have added accompanying text in the Results and Discussion sections.

4. On the contrary, big-2 prt6 35S:RAP2.3-HA displayed evident phenotypes, among which smaller rosettes, twisted leaves with abnormal surfaces, and reproductive defects. Similar phenotypic outcome has been associated before with the expression of an N-terminal truncation of RAP2.12, as reported in the same study cited previously (Giuntoli et al. PCE 2017), reasonably due to highly effective stabilization of the protein. There, some defect in cuticle deposition has been observed and linked to the leaf phenotype of the over-expressor. Given the similarities between cuticle and suberin biosynthesis, those previous observations should be referenced in the discussion (l. 373 may be a proper place, for instance). Overall, deltaN-RAP2.12, quadruple pco mutants (Weits et al., 2023; doi.org/10.1111/pce.14440) and the data presented in this manuscript align well to

suggest that high constitutive presence of ERFVII proteins prevents proper biosynthesis of surface polymers and suberin.

Thank you for this suggestion. We have referred to the similarities between our lines and those reported in Giuntoli et al., 2017, Weits et al., 2022 and Masson et al., 2019 which are now referenced in the revised Discussion section. To explore the possibility of cuticular defects in RAP2.3-HA over-expressors, we used Toluidine Blue staining of seedlings. We observed light staining of coleoptiles in *prt6-5* and *prt6-5-/- big-2+/-* seedlings expressing RAP2.3-HA, indicative of a cuticular defect. The images are presented in Supplemental Fig. 3.

5. The analysis in Fig. 5A-B led the Authors to conclude that both ERFVII-dependent and BIG-dependent regulation occurs in roots during suberin deposition. It would be interesting to extend the analysis of the genotypes in Fig. 5B to the expression of genes from Fig. 5A that are shared with cutin biosynthetic pathway (such as LACS2, CYP86A, or GPATs), to be measured in leaves of soil grown plants, to understand whether or not analogous distinct regulation may take place in the aerial organs (and connect to the previous point).

As the referee outlines above, the ability of ERFVIIs to modulate gene expression appears to decline through development (Giuntoli et al., 2017), suggesting that extending information regarding the regulation of suberin deposition in roots to cutin biosynthesis in leaves may not be straightforward. Therefore, whilst we agree that this is an interesting question, we consider that this is outside the scope of the current manuscript.

6. The observation that the PRT6 pathway and BIG are involved in suberin deposition in roots seems to have straightforward links with the physiological adaptations of the plant to flooded conditions. For instance, it may not be too far-fetched to speculate that the hypoxic response also includes the regulation of root permeability to water and solutes when the soil is flooded. The findings related to suberin synthesis are highlighted in the title and summary, but were eventually barely discussed. I believe that the readers may appreciate being guided through the perspectives or future directions of these findings a bit more.

We agree with this suggestion and have expanded the Discussion section accordingly.

Regarding the genetic interaction between PRT6 and BIG, it is defined as "synergistic" in several instances, however this term does not seem completely accurate, since big-2 mutation alone had no effects on the (molecular) phenotypes observed, to be added to prt6 effect in the combined mutant. , Synergy would imply the phenotype made by the two null mutations to be greater than the sum of their individual effects (doi:10.1016/j.tig.2009.06.004). PRT6 and BIG seem to rather have an epistatic relationship. A speculative molecular mechanism underlying this genetic interaction is contained in Fig. 6, whereby the gathered information on BIG participation in proteasome functions is summarized. The model makes sense, but it is still hard to figure out why BIG removal from the UPS complex should become relevant only after PRT6 is missing (if anything, one would expect some effect from BIG removal, rather than enhanced effect to PRT6 removal).

We agree with the first point and have removed the term “synergistically” from the text. Regarding the second point, our interpretation of the data is that PRT6 is the predominant N-recognin for Type 1 substrates such that removal of BIG alone does not have an impact. However, removing BIG in the *prt6* background prevents residual turnover of substrates. A similar argument could be applied for

PRT1. Ultimately, it will only be possible to test this in a reconstituted system with purified PRT6/PRT1, BIG and the respective E2 enzymes, which would undoubtedly be extremely challenging. Based on data from UBR4, we speculate that removal of BIG alone may have general effects on proteostasis that are unrelated to the N-degron pathway; for example, UBR4 has recently been shown to regulate proteolytic activity of the proteasome (Hunt et al., 2021 https://doi.org/10.1038/s41467-021-21738-8). This is consistent with the co-purification of UBR4 (and BIG) with UPL-type HECT E3 ligases, since ULPs increase processivity of polyubiquitination at the proteasome. Some of these effects may explain, at least in part, the pleiotropic phenotype of *big* mutants.

The relevant part of the Discussion section has been rewritten to clarify these points.

Finally, I have two additional questions:
• Clarification on the blots with the model substrates used in Fig. 1: the reference and reporter protein should be present in stoichiometric amounts, when the latter is fully stabilized, but the reference band is always much weaker in the blots. It this due to the different amounts of HA repetitions?

This is correct. There are three copies of the HA tag in the GUS reporter and one in the co-translated, metabolically stable reference protein, DHFR-Ub. as described in Garzón et al., 2007 https://doi.org/10.1016/j.febslet.2007.06.005. This has been clarified in the revised legend.

• Among the BIG mutants mentioned in the introduction, the only one missing is the one used in this study, big-2. It is unclear to me whether this study is the first one characterizing the mutant phenotype, or there may be more information to mention on it, from the source paper mentioned in Suppl Tab. 1 or subsequent studies.

As the manuscript states, there are many *big* mutants in the literature and different groups have reported on different ones. *big-2* was used in Kasajima et al., 2007 https://doi.org/10.1111/j.1399-3054.2006.00814.x and in Ivanova et al., 2014 https://doi.org/10.1104/pp.114.237495) This is now mentioned in the revised manuscript.

Reviewer #3 (Comments for the Author):

This manuscript describes functions of the BIG protein in Arabidopsis using genetics and biochemical methods. BIG is a member of the conserved UBR protein family in plants, yeast and animals that is involved in the recognition of different N termini of proteins and facilitation of their degradation. Some plant UBRs have been characterised such as PRT6 that targets basic N-termini, but mechanisms that target other N-termini types are not well characterised in plants. The N degron pathway maintains protein levels in a wide variety of developmental and environmental responses, therefore it is a very important and relatively uncharacterised aspect of plant biology.
Mutants in the BIG protein, a UBR homolog, have a wide range of phenotypes in Arabidopsis but it's potential role in degron pathways is not known. Here the authors show a role for BIG in protein degradation and describe its function with respect to another UBR protein PRT6 and

another N- degron recognin PRT1. New processes influenced by these protein degradation systems such as suberin formation have been described.

General comments

The manuscript covers an important but under-characterised area of plant biology. The approaches taken are appropriate and insightful, working to the biochemical strengths of the authors. The work is thorough (includes VRN2 and hypoxia proteins) and mostly well described, and the conclusions are supported by the evidence.

We thank the referee for these positive comments.

Specific comments
Introduction

It could be shortened a little- it's quite review-like in places especially the N degron system.
L115 how many UBR family proteins are there in Arabidopsis?
Provide a more detailed description of BIG function from work in other systems and what could be expected from this knowledge

Given the diverse backgrounds of the journal's audience, we feel that it is important to provide a clear explanation not only of the N-degron pathway structure but also its physiological relevance for the non-specialist reader. Therefore, we consider that the length is appropriate.

We have added a sentence referencing the UBR family proteins of Arabidopsis and further information about BIG homologues in other systems, as requested.

Results

L162-164. The reader needs a clearer description of what the type 1 substrate and type 2 substrates are and what the stable M-GUS is exactly. The Introduction describes type 1 degrons but not type 2 as far as I can make out. The authors need to make sure that they pitch the experimental results as clearly as possible to the general plant reader.

Figure 1. Need to describe R- and F- GUS and M-GUS precisely in the Figure and Legend. It's not clear enough to the non-expert. Components of Fig S1B, C need to be in the main Figure for direct comparison of M-GUS with the R- and M-GUS experiments.

Thank you for these suggestions. The Introduction defines type 1 and type 2 substrates, but these terms have now been clarified in the relevant part of the Results section. All the X-GUS panels have been incorporated into the revised version of Figure 1, and the legend has been edited to give a clearer description of how the substrates are generated in planta. The panels describing the proximity labelling assay have been moved from the original Figure 1 to a separate figure (new Fig. 2) for reasons of space and clarity.

L169. Re-phrase "removal of N-recognin function" sentence to make it clearer.

This sentence has been rephrased and now reads: "R-GUS and F-GUS were stabilised in *prt6-1* and *prt1-1* mutants respectively, as previously reported".

L172-174. Fig 1 C and D show the absence of the precursor HA-GUS fusions in big1-2 relative to the HA-DHFR stability protein. Isn't it clearer to state that the GUS fusions require BIG function for their stability? I don't understand how it's possible to conclude that BIG mediates R- and F-GUS degradation? This whole section describing Fig 1C and 1D needs to be described more clearly. Are big mutant plants relatively sick and have pleiotropic effects eg Fig 2B? They don't look sick from for Fig 1E.

The conclusion that BIG mediates R- and F-GUS degradation is based on the observation that the intensity of the reporter proteins is increased in *prt6-1 big-2* relative to *prt6-1* (which is clear from the short exposure panel, also considering the stoichiometry with the stable reference). We agree that it is curious that the intact DHFR-Ub-R-GUS fusion is not detected in *big-2* but attribute this to the slightly different developmental stage of the mutant. Fig. 1E (now 1G in the revision) shows 6 d-old seedlings grown on plates containing sugar: this is the condition in which *big-2* most closely resembles WT and *prt6* in terms of development. However, the seedlings are still smaller, and it is indeed true that the mutant is pleiotropic, as widely reported.

L186. To comprehend Figure 1F a diagram of the Turbo ID components is needed in the Figure. The reader should not have to rifle through supplemental Figures for key information.

Thank you for this suggestion. We have created a new figure (Fig. 2 in the revised manuscript) and moved the relevant elements from Figure S1 to this main figure, which we trust has improved the clarity.

Figure 2, what do the A, B, C, D letters mean on the graphs with respect to significance levels?

This is now Fig. 3 in the revision. As noted in the legend, different letters indicate significant differences between conditions (P>0.05).

L205. Need to describe specifically the role of ERFVIIs in the hypoxia response.

This sentence now reads: "Consistent with the known role of ERFVIIs in inducing hypoxia responsive gene expression, the enhanced HRE2-HA protein abundance in *prt6-5 big-2* relative to *prt6-5* was accompanied by a much stronger induction of the core hypoxia genes *ALCOHOL DEHYDROGENASE* (*ADH*) and *PHYTOGLOBIN1* (*PGB1*) and the respective proteins (Fig. 32A, C)." The role of ERFVIIs is also covered in the Introduction.

Discussion

L350. The sentence starting "Notably..." should be made more accessible by describing the functions of ATE and PRT6 proteins, not the mutants. The general reader needs this information in front of them.

The sentence: "The PRT6 N-recognin influences the stability of Type 1 substrates such as those initiating with R that can be generated by the action of protein cleavage and/or arginyl t-RNA protein transferase enzymes (ATE) (Garzón et al., 2007; Graciet et al. 2009)." Has been inserted to address this point.

Use knowledge from other systems to understand the interactions of BIG family proteins and PRT6 eg do they interact?. In particular explain the phenotype of increased substrate turnover in big mutants, and reduced turnover in big prt6 mutants. In the absence of BIG function does PRT6 target the substrate protein for higher levels of degradation?

We have added further information about UBR4 to the Introduction and have modified the Discussion section to address this question. As outlined above, we are not convinced that there is increased substrate turnover in big single mutants.

Reviewing Editor comments:
Overall, some more experimentation and much more clear description are required to reach to necessary standard for Plant Cell.

We have tried to address all the points outlined above and trust that the manuscript is substantially improved.

Rebuttal references

Barnsby-Greer L, Mabbitt PD, Dery MA, Squair DR, Wood NT, Lamoliatte F, Lange SM, Virdee S. (2024) UBE2A and UBE2B are recruited by an atypical E3 ligase module in UBR4. Nat Struct Mol Biol. doi: 10.1038/s41594-023-01192-4.

Garzón M, Eifler K, Faust A, Scheel H, Hofmann K, Koncz C, Yephremov A, Bachmair A. (2007) PRT6/At5g02310 encodes an Arabidopsis ubiquitin ligase of the N-end rule pathway with arginine specificity and is not the *CER3* locus. FEBS Lett. 581: 3189-3196

Hunt LC, Schadeberg B, Stover J, Haugen B, Pagala V, Wang YD, Puglise J, Barton ER, Peng J, Demontis F. Antagonistic control of myofiber size and muscle protein quality control by the ubiquitin ligase UBR4 during aging. Nat Commun. 2021 Mar 3;12(1):1418.

Ivanova A, Law SR, Narsai R, Duncan O, Lee JH, Zhang B, Van Aken O, Radomiljac JD, van der Merwe M, Yi K, Whelan J. 2014 A Functional Antagonistic Relationship between Auxin and Mitochondrial Retrograde Signaling Regulates Alternative Oxidase1a Expression in Arabidopsis. Plant Physiol. 165: 1233-1254.

Kasajima I, Ohkama-Ohtsu N, Ide Y, Haysashi H, Yoneyama T, Suzuki Y, Naito S, Fujiwara T. (2007) The BIG gene is involved in regulation of sulfur deficiency-responsive genes in Arabidopsis thaliana. Physiol. Plant. 129: 351-363

Meinnel and Giglione, 2022 N-terminal modifications, the associated processing machinery, and their evolution in plastid-containing organisms. J Exp. Bot., 73, 6013–6033

Sasidharan R, Mustroph A. 2011 Plant oxygen sensing is mediated by the N-end rule pathway: a milestone in plant anaerobiosis. Plant Cell. 23(12):4173-83.

Wang K, Froehlich JE, Zienkiewicz A, Hersh HL, Benning C. A Plastid Phosphatidylglycerol Lipase Contributes to the Export of Acyl Groups from Plastids for Seed Oil Biosynthesis. Plant Cell. 2017 Jul;29(7):1678-1696

1st Revision - Editorial Decision 14-Mar-2024

Dear Dr. Frederica Theodoulou:

We have received reviews of your manuscript entitled "BIG enhances protein degradation through the Arg/N-degron pathway to regulate the hypoxia response and suberin deposition in Arabidopsis.." On the basis of the advice received, the board of reviewing editors would like to accept your manuscript for publication in The Plant Cell. This acceptance is contingent on revision based on the comments of our reviewers. In particular, please consider the following:

As you will see all three Reviewers consider that the manuscript has been improved and is now acceptable. However, I ask you to consider the few points suggested by Reviewer #1 for your final version.

Please highlight all changes and include a detailed annotation of changes of the text, with line numbers, and noting your responses to the comments.

To submit your revised manuscript, click:

Link Not Available

If you have any questions about the revision submission procedures, please contact the Editorial Office Staff (tpc-submissions@aspb.org). If you cannot return the revised manuscript within 30 days, please let us know. Otherwise, we will assume that you have elected not to revise the manuscript and withdraw it.

Thank you very much for the privilege of reviewing this work. I look forward to receiving the next version.

Sincerely,

The Plant Cell Editorial Board

Reviewer #1 (Comments for the Author):

I very much enjoyed the updated and much more detailed discussion. The manuscript was substantially improved by editing text, data representation and adding additional results from new experiments. As far as I can see, all asked questions were addressed and excellently answered.

All my concerns and comments were fully addressed, this is especially true for the questions about the used mutant big-2 allele and its complementation, and some concerning the usage of the protein stability reporter constructs in various mutant backgrounds. All this has very thoroughly been explained in the response to the referees. I think that it would be excellent, if some of these additional sentences could also show up in the published manuscript, see comments below.
Regarding the big-2 allele, the authors explained very carefully the state-of-the-art of the topic and included valuable information in the response to the reviewers' section ("As it has a severe morphological phenotype [...]"). It would be good if they could include this part in the main text as well, at least I couldn't find it when checking the revised manuscript. It would help to extend both to the question for potential residual function of the truncated version resulting from the T-DNA insertion as well as to the mentioned other mammalian homologous sequences (UBR4).
The authors addressed the concerns and remarks about the used big-2 allele by sequencing the stock. The results highlighted the importance and are a truly good example of proper characterizations of used reagents and stocks even if they were used for published work already. The authors should include information on the accomplished sequencing of the big-2 allele which importantly lead to the result of finding a second site insertion (plip1-3). They also should mention (like in the responses to the referees), that plip1 alleles have a rather mild phenotype which is in contrast to big-2 and properly reference to Wang et al. Plant Cell 2017.

The authors included new data such as the hypoxia assays and suberin quantifications, cuticle stainings were done as well highlighting the pleiotropic involvement of BIG function in plant physiology.

Fig. 7 mentions both UBC2 and 3 to work with PRT6. As far as I know, this has only been shown (in a heterologous assay in yeast) for UBC2. If there is no specific reason for also mentioning UBC3 here, it should be omitted. Also, the reference Kozlic et al. 2022 does not address UBC3. On the other hand, also interacting UBCs for PRT1 - in thevsame figure .- have been reported in in vitro studies (UBC8, Mot et al. 2018).

I also find the additional comments for l. 202 ff. very helpful but I am not sure if this can be somehow included in the manuscript.

It would certainly have value in order to judge the reliability of the used reagents and lines. In line with this, the authors should stress that, instead of utilising independent insertion events of the same single reporter construct, rather showed increased abundance of five different substrates in prt6 big-2 mutants (R-GUS, F-GUS, R-LUC, HRE2 and VRN2). I'd always prefer the latter, if I had the choice.

There is one sentence added as response to reviewer 3 comments: "The PRT6 N-recognin influences the stability of Type 1 substrates such as those initiating with R that can be generated by the action of protein cleavage and/or arginyl t-RNA protein transferase enzymes (ATE) (Garzón et al., 2007; Graciet et al. 2009)." The two references are somewhat classics in the field and describe the isolation of mutants of PRT6 (Garzón et al., 2007) and one of the two first phenotypic characterizations of prt6 and the ate1 ate2 double mutant. However, if the authors would like to refer to either "PRT6 N-recognin influences the stability of Type 1 substrates such as those initiating with R" or "action of protein cleavage and/or arginyl t-RNA protein transferase enzymes (ATE)", they should rather use more specific references (which they have already introduced elsewhere) that demonstrated exactly the discussed processes of destabilization and protein modification, e.g. Gibbs et al. 2011 and Licausi etal. 2011, both nature for the first, and White et al. 2017 Nat Comms for the latter.

I also checked the comments by the other two referees and they were more than addressed in my opinion. I also suggest to include some of the explanations of the big-2 phenotype made by the authors stated after Question 2 of Reviewer 2 (after "The claims made on the contribution of hypoxia survival") in the published paper. There are so many valuable discussion points in the responses to the referees, I would not want to have it excluded from the real paper. Some ideas are beyond the scope of this paper, although truly intriguing and worth following up such as cross-regulation of the cutin biosynthetic pathway...

I suggest to accept this MS asap.

Reviewer #2 (Comments for the Author):

After careful reading of its revised version, I found the manuscript by Zhang and colleagues fully informative on the few aspects that were raised in the first round of revision. All points on which I personally commented were addressed in the text and/or in the rebuttal, which I also enjoyed reading by itself.
Beyond the fact that some of the comments were accommodated in the revision, by expanded data discussion and clarification of the background information, I appreciate the effort the Authors made to include new data. Among them, the experiments supporting cuticle defects (Suppl. Fig. 3) and the evaluation of plant survival (Suppl. Fig. 7) in my opinion not only strengthen the manuscript, but also provide information that, along with its discussion, will be valued by the low oxygen stress community.

Reviewer #3 (Comments for the Author):

I think you have done a good job of clarifying your results section such that it has a reasonably clear narrative flow. I think the description of BIG as a possible new N-recognin will stimulate new interest in this hard-to-study protein.

I still find the Introduction to be a bit turgid, but that seems to be a preferred style that should not be a consideration for publication. But it might affect reader accessibility

Please also note the following:

-The Plant Cell now requires authors to complete and submit an author revisions checklist upon submission of a revised manuscript. The aim of the checklist is to aid authors in preparing a high-quality manuscript, facilitate the review and assessment of revised manuscripts, and help to ensure that journal standards are maintained across the board. If your manuscript is accepted, the completed checklist will be published as supplemental material attached to the article online. Please download a copy of the checklist (pdf fillable form) at this link, for submission with your revised manuscript: https://tpc.msubmit.net/html/Author_Revisions_Checklist.pdf.

-Supplemental materials should be restricted to large datasets and tables, presentation of replicates, and validation of reagents, methods, or genotypes. Any data that are used to support the major claims must be in the main manuscript. Supplemental figure legends must indicate what figure in the main manuscript is supported by the supplemental data presented. Please justify how each of the supplemental figures meet the criteria.

-Sampling methods and the nature of "biological replicates" should be described precisely (i.e. different plants, parts of plants,

pooled tissue, independent pools of tissue, sampled at different times, etc.), along with a clear description of and rationale for any statistical analyses conducted. The reader should know exactly what was sampled; what forms the basis of the calculation of any means and statistical parameters reported. This is also necessary to ensure that proper statistical analysis was conducted.

Response to referees
Reviewer #1 (Comments for the Author):

I very much enjoyed the updated and much more detailed discussion. The manuscript was substantially improved by editing text, data representation and adding additional results from new experiments. As far as I can see, all asked questions were addressed and excellently answered.

Thank you!

All my concerns and comments were fully addressed, this is especially true for the questions about the used mutant big-2 allele and its complementation, and some concerning the usage of the protein stability reporter constructs in various mutant backgrounds. All this has very thoroughly been explained in the response to the referees. I think that it would be excellent, if some of these additional sentences could also show up in the published manuscript, see comments below.

Regarding the big-2 allele, the authors explained very carefully the state-of-the-art of the topic and included valuable information in the response to the reviewers' section ("As it has a severe morphological phenotype [...]"). It would be good if they could include this part in the main text as well, at least I couldn't find it when checking the revised manuscript. It would help to extend both to the question for potential residual function of the truncated version resulting from the T-DNA insertion as well as to the mentioned other mammalian homologous sequences (UBR4).

Thank you for this suggestion. We have included a revised version of this text to the Discussion (lines 445-457).

The authors addressed the concerns and remarks about the used big-2 allele by sequencing the stock. The results highlighted the importance and are a truly good example of proper characterizations of used reagents and stocks even if they were used for published work already. The authors should include information on the accomplished sequencing of the big-2 allele which importantly lead to the result of finding a second site insertion (plip1-3).

The manuscript already contains a diagram of the *plip1-3* allele in Fig. S1E, and details of the sequencing are presented in the Methods section. However, we have indicated the genomic position of the T-DNA insertion in the Fig. S1 legend and have added the sentence: "The positions of T-DNAs were identified by a BLAST search using the pBIN-pROK2 insertion sequences (http://signal.salk.edu/cgi-bin/tdnaexpress) as query." to the methods section (lines 644-645).

They also should mention (like in the responses to the referees), that plip1 alleles have a rather mild phenotype which is in contrast to big-2 and properly reference to Wang et al. Plant Cell 2017.

We have added the sentence "We consider it unlikely that a lesion in *PLIP1* would lead to stabilisation of N-degron pathway substrates." to the discussion (lines 416-417). We prefer not to refer to the mild phenotype of *plip* mutants because the morphological phenotypes of *big* alleles may not be related to N-degron pathway functions.

The authors included new data such as the hypoxia assays and suberin quantifications, cuticle stainings were done as well highlighting the pleiotropic involvement of BIG function in plant physiology.

Fig. 7 mentions both UBC2 and 3 to work with PRT6. As far as I know, this has only been shown (in a heterologous assay in yeast) for UBC2. If there is no specific reason for also mentioning UBC3 here, it

should be omitted. Also, the reference Kozlic et al. 2022 does not address UBC3. On the other hand, also interacting UBCs for PRT1 - in the same figure .- have been reported in in vitro studies (UBC8, Mot et al. 2018).

Thank you for pointing this out. The figure and legend have been edited accordingly.

I also find the additional comments for l. 202 ff. very helpful but I am not sure if this can be somehow included in the manuscript. It would certainly have value in order to judge the reliability of the used reagents and lines. In line with this, the authors should stress that, instead of utilising independent insertion events of the same single reporter construct, rather showed increased abundance of five different substrates in prt6 big-2 mutants (R-GUS, F-GUS, R-LUC, HRE2 and VRN2). I'd always prefer the latter, if I had the choice.

We struggled to incorporate this in the manuscript without disrupting the flow of the text. We trust that these useful comments will be available in the published version of the response to referees.

There is one sentence added as response to reviewer 3 comments: "The PRT6 N-recognin influences the stability of Type 1 substrates such as those initiating with R that can be generated by the action of protein cleavage and/or arginyl t-RNA protein transferase enzymes (ATE) (Garzón et al., 2007; Graciet et al. 2009)." The two references are somewhat classics in the field and describe the isolation of mutants of PRT6 (Garzón et al., 2007) and one of the two first phenotypic characterizations of prt6 and the ate1 ate2 double mutant. However, if the authors would like to refer to either "PRT6 N-recognin influences the stability of Type 1 substrates such as those initiating with R" or "action of protein cleavage and/or arginyl t-RNA protein transferase enzymes (ATE)", they should rather use more specific references (which they have already introduced elsewhere) that demonstrated exactly the discussed processes of destabilization and protein modification, e.g. Gibbs et al. 2011 and Licausi et al. 2011, both nature for the first, and White et al. 2017 Nat Comms for the latter.

These references have been added as requested (line 434).

I also checked the comments by the other two referees and they were more than addressed in my opinion. I also suggest to include some of the explanations of the big-2 phenotype made by the authors stated after Question 2 of Reviewer 2 (after "The claims made on the contribution of hypoxia survival") in the published paper.

The Discussion section stated: "Additional approaches to explore low oxygen tolerance were challenging due to the pleiotropic phenotype of *big-2* plants, in which primary root elongation and lateral root development are severely impaired (Ruegger et al., 1997; López-Bucio et al. 2005; Yamaguchi et al. 2007; Guo et al., 2013).", which we felt addressed this point. However, we have replaced this with a clarified version: "Additional approaches to explore low oxygen tolerance were explored, however, it was challenging to correlate increased expression of hypoxia responsive genes with hypoxia tolerance against a backdrop of a pleiotropic mutant phenotype. Primary root elongation and lateral root development are severely impaired in *big-2* (Ruegger et al., 1997; López-Bucio et al. 2005; Yamaguchi et al. 2007; Guo et al., 2013), and we ascribe the waterlogging sensitivity of *big-2* and *prt6 big-2* (Supplemental Fig. 7B) to their highly reduced root systems." (lines 488-495).

There are so many valuable discussion points in the responses to the referees, I would not want to have it excluded from the real paper. Some ideas are beyond the scope of this paper, although truly intriguing and worth following up such as cross-regulation of the cutin biosynthetic pathway...

I suggest to accept this MS asap.

Dear Dr. Frederica Theodoulou:

We are pleased to inform you that your paper entitled "BIG enhances protein degradation through the Arg/N-degron pathway to regulate the hypoxia response and suberin deposition in Arabidopsis." has been accepted for publication in The Plant Cell, pending a final editorial review by a science editor. At this stage, your manuscript will be evaluated by a science editor with respect to its presentation of scientific content, compliance with journal policies, and presentation for a broad readership. The Plant Cell has contracted with Plant Editors (planteditors.com) to provide this service to our authors, and you will soon receive additional information on this process.

Please note that each author needs to link their ORCID identifier to their account in the system before your manuscript can be published. If any authors do not have an ORCID linked to their account, they will receive a message with a link to complete this task. Please ensure that ALL of your coauthors have completed this task as soon as possible.

ASPB offers an OPEN option that allows authors to have their online articles available for free to all users immediately upon publication. For more information about the ASPB OPEN option, refer to the Final Submission Checklist Form.

The Plant Cell and The Arabidopsis Information Resource (TAIR) are collaborating to collect functional annotation data about Arabidopsis genes from authors. This includes information about the gene's molecular function (e.g., kinase activity, ATP synthetase activity), the biological process/es it is involved in (e.g., endosperm development, threonine biosynthesis), its subcellular location (e.g., nucleus, ER), anatomical or developmental expression pattern (e.g., leaf, ovule, flower stage 10, seedling stage), or its partner in a protein-protein interaction (e.g., AT1G01010 interacts with AT1G01020).

If your paper contains results falling into one or more of these categories for Arabidopsis genes, we request that you now submit these data for inclusion in TAIR by filling in the form provided at the following URL: https://www.arabidopsis.org/doc/submit/functional_annotation/123. If you need further clarification on what types of data can be submitted please contact curator@arabidopsis.org.

Finally, we encourage your submission of artwork for the journal cover. Monthly issues will have an online cover image and selected covers will be used for posters, other promotional items, and "wallpaper" for mobile devices. For more information, refer to Cover Submission in the Instructions for Authors https://tpc.msubmit.net/cgi-bin/main.plex?form_type=display_auth_instructions.

We look forward to seeing your paper published.

Sincerely,

The Plant Cell Editorial Board

=====
IMPORTANT REMINDER: PEER REVIEW REPORTS
=====

If you opted to publish a peer review report along with your article during the original submission process, it will be prepared by the editorial staff and publicly posted with your manuscript, inside the zip file that contains any other supplemental material. As a reminder, the peer review report is a public record of all comments from editors and reviewers, as well as your prior responses, as you received them in the decision letters for each draft of your manuscript. If you agreed to publish this report and have changed your mind, or are not sure if you selected this option, please contact the editorial office as soon as possible before signing the license agreement from our publisher.

----FOR ASPB OFFICE USE ONLY (DO NOT EDIT)----
MSID: 35025
Corresponding Author: Prof. Theodoulou
